# Genomic analyses of an extensive collection of wild and cultivated accessions provide new insights into peach breeding history

**DOI:** 10.1186/s13059-019-1648-9

**Published:** 2019-02-21

**Authors:** Yong Li, Ke Cao, Gengrui Zhu, Weichao Fang, Changwen Chen, Xinwei Wang, Pei Zhao, Jian Guo, Tiyu Ding, Liping Guan, Qian Zhang, Wenwu Guo, Zhangjun Fei, Lirong Wang

**Affiliations:** 1 Zhengzhou Fruit Research Institute, Chinese Academy of Agricultural Sciences, Zhengzhou, China; 2 Boyce Thompson Institute for Plant Research, US Department of Agriculture (USDA) Robert W. Holley Center for Agriculture and Health, Cornell University, Ithaca, NY, USA; 3 Key Laboratory of Horticultural Plant Biology (Ministry of Education), College of Horticulture and Forestry Sciences, Huazhong Agricultural University, Wuhan, China

**Keywords:** Genomics, Whole genome resequencing, Domestication, Improvement, Human selection, Breeding history

## Abstract

**Background:**

Human selection has a long history of transforming crop genomes. Peach (*Prunus persica*) has undergone more than 5000 years of domestication that led to remarkable changes in a series of agronomically important traits, but genetic bases underlying these changes and the effects of artificial selection on genomic diversity are not well understood.

**Results:**

Here, we report a comprehensive analysis of peach evolution based on genome sequences of 480 wild and cultivated accessions. By focusing on a set of quantitative trait loci (QTLs), we provide evidence supporting that distinct phases of domestication and improvement have led to an increase in fruit size and taste and extended its geographic distribution. Fruit size was predominantly selected during domestication, and selection for large fruits has led to the loss of genetic diversity in several fruit weight QTLs. In contrast, fruit taste-related QTLs were successively selected for by domestication and improvement, with more QTLs selected for during improvement. Genome-wide association studies of 11 agronomic traits suggest a set of candidate genes controlling these traits and potential markers for molecular breeding. Candidate loci for genes that contributed to the adaption to low-chill regions were identified. Furthermore, the genomic bases of divergent selection for fruit texture and local breeding for different flavors between Asian and European/North American cultivars were also determined.

**Conclusions:**

Our results elucidate the genetic basis of peach evolution and provide new resources for future genomics-guided peach breeding.

**Electronic supplementary material:**

The online version of this article (10.1186/s13059-019-1648-9) contains supplementary material, which is available to authorized users.

## Background

Global food production and crop quality have been transformed over the last 10,000 years through domestication and extensive selection [1]. However, crop breeding still relies heavily on experience and manual observation, which are both limiting in terms of efficiency and time-consuming, especially for perennial fruit crops with long juvenile phases. Recently developed next-generation DNA sequencing technologies allow the tracking of genome-wide selection signatures and can be used to develop strategies for further crop improvement, as recently reported in rice (*Oryza sativa*) [2], maize (*Zea mays*) [3], cucumber (*Cucumis sativus*) [4], tomato (*Solanum lycopersicum*) [5], soybean (*Glycine max*) [6], peach (*Prunus persica*) [7], and apple (*Malus domestica*) [8]. Genome-wide association studies (GWAS) have been performed to identify loci or genes associated with important agronomic traits in a range of species [2, 5–8] and to facilitate targeted and precise genetic selection, involving marker-assisted selection (MAS) and molecular design breeding. MAS has been applied to woody perennial crops as well as annual crops, where it has accelerated breeding programs through early identification of fruit-related traits at the seedling or juvenile phases [9, 10].

Fruits represent an important component of the diet of humans and other animals. Peach (*Prunus persica* L.) is one of the most economically important fruit crops in temperate regions, with a global yield of 25.0 million tons in 2016 and a net value of over $12.4 billion in 2014 (FAOSTAT; http://faostat.fao.org). Peach is also considered to be a model system for molecular biology researches in the Rosaceae family and represents one of the fruit species most consumed worldwide. Following domestication in China, approximately 5000 years ago, peach has undergone a long period of selection by native farmers, and in recent decades, specific target-guided breeding has led to a remarkable increase in fruit quality. However, the selection signatures at the molecular level associated with domestication and improvement remain largely unclear. Understanding the genetic basis of selection is important for precise molecular breeding and for eliminating undesired costs, such as linkage drag during wild introgressions [5]. Although previous studies have attempted to address this issue, small population sizes and underrepresentation of the accessions in the germplasm collection limited the findings [7, 11].

**Fig. 1:**
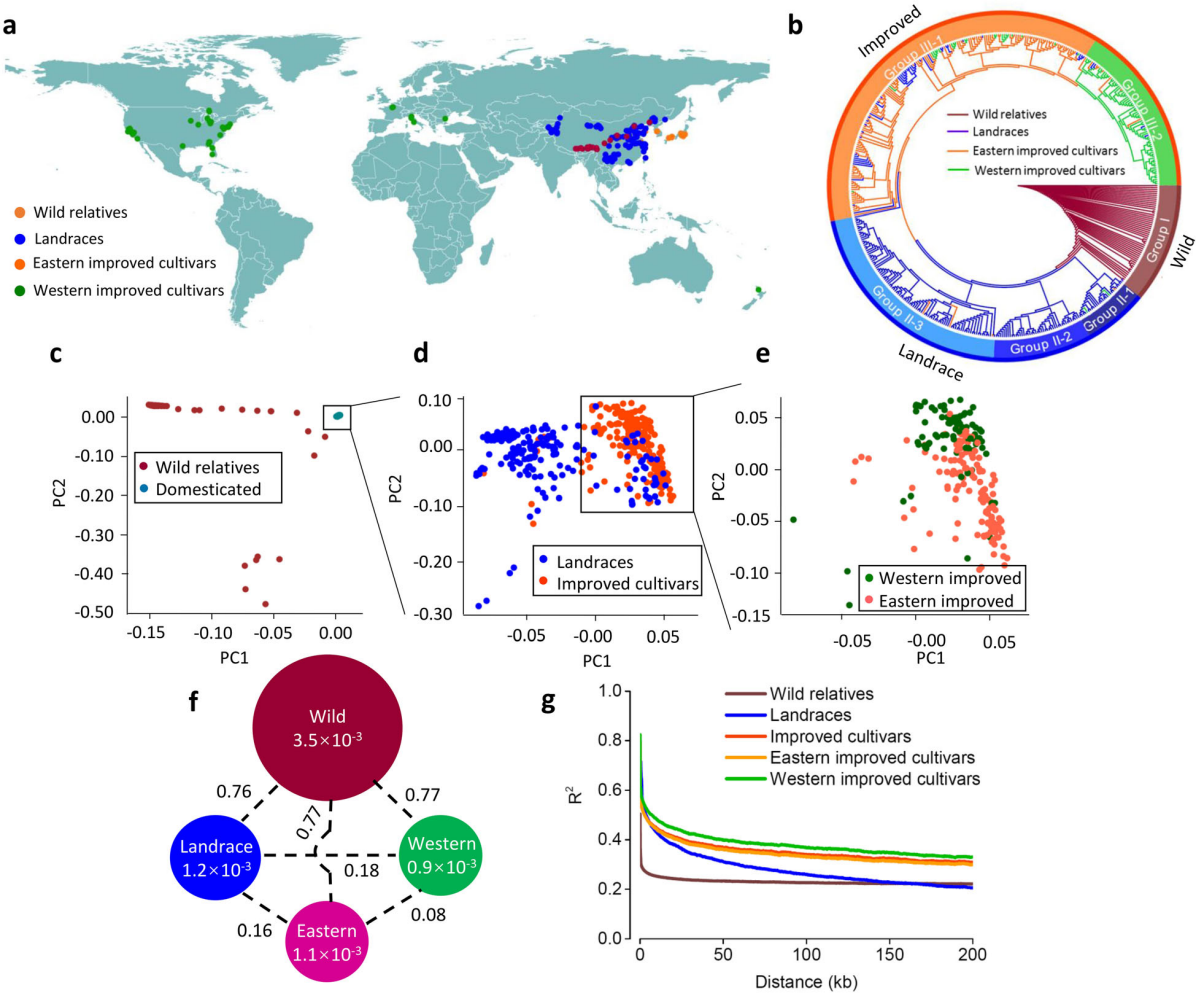
Phylogeny and population genetics of domesticated and wild peaches. **a** Geographic distribution of the 480 peach accessions, each of which is represented by a dot on the world map. **b** The neighbor-joining phylogenetic tree of 480 peach accessions constructed based on 4.98 million SNPs. **c**–**e** PCA plots of peach accessions. Six subgroups are indicated by different colors indicated at the bottom of each figure. The domesticated group includes the landraces and improved cultivars. The improved cultivars include the accessions from eastern and western countries. **f** Summary of nucleotide diversity (*π*) and population divergence (*F*_ST_) across the four groups. Value in each circle represents nucleotide diversity for the group, and values between pairs indicate population divergence (*F*_ST_). “Western” and “Eastern” indicated improved cultivars from western and eastern countries, respectively. **g** Decay of linkage disequilibrium (LD), measured by *r*^2^, in the five groups

To explore this question more comprehensively, we sequenced the genomes of a large peach collection, consisting of 480 diverse accessions from around the world, including 52 wild relatives, 213 landraces, and 215 improved cultivars (Fig. 1a, Additional file 1: Table S1). Among the improved cultivars, 126 were collected from eastern countries (Asia) and 89 were from western countries (North America and Europe). Using these sequencing data, here we elucidate the breeding history of peach by identifying loci and genes associated with important agronomic traits that have been under selection during domestication and improvement. Our data indicate that fruit size was mainly selected during domestication, while fruit taste was successively selected during both domestication and improvement. Our results provide a new resource for further molecular breeding and studies of peach biology.

## Results and discussion

### A high-density peach genomic variation map

Through sequencing of 480 peach accessions (Fig. 1a), we generated a total of 719 Gb base pairs of sequences, with an average depth of 6.4× and a coverage of 93.5% for each accession (Additional file 1: Table S1). Pair-end reads were mapped against the peach reference genome [12] (release version 2.0), and a final set of 4,980,259 high-quality single nucleotide polymorphisms (SNPs) were identified, resulting in an average of 21.9 SNPs per kilobase (Additional file 2: Tables S2 and S3, Additional file 3: Figure S1). A total of 886,033 SNPs were located in coding regions, including 457,988 nonsynonymous and 430,034 synonymous SNPs (Additional file 2: Table S3). We also found 8812 SNPs in 5724 genes that were predicted to have significant effects on gene functions, including 5784 nonsense SNPs in 4480 genes that resulted in start codon changes, the introduction of premature stop codons, or the production of elongated transcripts. The accuracies of original SNPs and SNPs following imputation were estimated to be 96.1% and 96.3%, respectively, based on genotyping using the Sequenom MassARRAY platform for 30 SNPs in 258 accessions (Additional file 1: Tables S1, Additional file 2: Table S4). In addition, we identified 1,026,375 small insertions and deletions (INDELs) (< 5 bp) and 159,330 large structural variations (SVs) (Additional file 3: Figure S1, Additional file 2: Table S2).

The high-density SNP data enabled GWAS analysis of seven agronomic traits: flesh color (white/yellow), fruit hairiness (peach/nectarine), fruit shape (round/flat), fruit texture (melting/non-melting), flesh adhesion (adhesion/freestone), pollen fertility (male sterility/fertility), and fruit skin color (red/non-red) (Additional file 3: Figure S2), in addition to the four (fruit weight, total phenolic content, fruit soluble solid content, and chilling requirement) that were described separately in the following sections. For quantitative traits, we identified a total of 20 novel quantitative trait loci (QTLs) using GWAS. For qualitative traits, the association signals were close to, or included, known loci or genes that have been identified previously using studies of recombinant populations [13–17] (Additional file 3: Figure S2, Additional file 2: Table S5). Specifically, these associations included a SNP (Pp01, 27,005,584 bp) located 392 kb from the *PpCCD4* gene for flesh color [13], a SNP (Pp06, 26,288,291 bp) within the *S* region for fruit shape [14], a SNP (Pp05, 16,633,286 bp) located 671 kb from *PpMYB25* for fruit hairiness [15], a SNP (Pp04, 19,909,362) located 860 kb from *PpendoPG* for fruit texture [16], a SNP (Pp06, 2,014,933) within the *Ps* region for male sterility [14], a SNP (Pp06, 19,070,801) located 11 kb from *PpendoPG* for flesh adhesion [16], and a SNP (Pp03, 18,103,021) within the *skc* region for fruit skin color [17] (Additional file 1: Table S5). We also carried out GWAS of these seven traits using genome-wide SVs and found that these results were highly consistent with those using the SNPs. Additionally, some candidate causative SVs were identified (Additional file 2: Table S5, Additional file 3: Figure S2), such as a 70.5-kb deletion associated with a non-melting phenotype in a gene cluster encoding endopolygalacturonase on chromosome 4 (Additional file 3: Figure S3), which was consistent with a previous study [16]. These associations will likely be useful for marker-based early selection and accelerated breeding for these seven commercially important fruit-related traits.

### Peach population structure and domestication bottleneck

To understand the genetic relationships among accessions, we constructed a neighbor-joining phylogenetic tree and performed a principal component analysis (PCA) using the 4,980,259 SNPs. The results of neighbor-joining tree and PCA largely supported the classification of the peach accessions into three major groups (Fig. 1b–e; Additional file 3: Figure S4). Group I included all wild relatives (wild group), while groups II and III were biased towards landraces (landrace group) and improved cultivars (improved group), respectively (Fig. 1a, b; Additional file 3: Figure S4). The domesticated accessions, including landraces and improved cultivars, formed a monophyletic lineage, indicating that all currently grown cultivated peach accessions originated from a single domestication event (Fig. 1b). Group II was further classified into three subclades, which showed strong geographic distribution patterns (Fig. 1a, b; Additional file 1: Table S1). Group II-1 contained ornamental accessions and landraces from northeastern and southern China. Group II-2 mainly consisted of landraces from the middle and lower reaches of the Yangtze river (Yangtze regions) and Yungui Plateau. Group II-3 mainly included landraces from northwestern and northern China. Similarly, accessions from group III were further clustered into two subclades, designated as the eastern improved (group III-1) and the western improved subgroups (group III-2), with group III-1 biased towards improved cultivars from Asia and group III-2 biased towards improved cultivars from North America and Europe (Fig. 1a, b; Additional file 1: Table S1). We interpret this geographic clustering pattern to indicate the introduction and development history of peach domestication, spread, and subsequent improvement. We also found that some cultivated peach accessions harbored admixed ancestry, suggesting that they might have experienced introgression or gene flow during breeding (Additional file 3: Figure S5).

The overall nucleotide diversity measured by the *π* value of the 480 accessions was 1.1 × 10^− 3^. The wild progenitors of cultivated peach showed a much higher nucleotide diversity (3.5 × 10^− 3^) than landraces (1.2 × 10^− 3^), suggesting a narrow domestication bottleneck (*π*_wild_/*π*_landrace_ = 2.92) (Fig. 1f), which was different from that reported in a previous study [18]. Nearly two thirds of the genetic diversity have been lost during peach domestication, indicating a major effect of artificial selection on the peach genomes. This pattern of domestication is different from other perennial fruit crops, such as grape [19] (*Vitis vinifera*) and apple [8], which have been reported to lack narrow domestication bottlenecks (Additional file 2: Table S6). We further verified the domestication bottleneck (*P* < 0.001) using the BOTTLENECK program which detects reductions in recent effective population size [20] (Additional file 2: Table S7). Moreover, the high genetic differentiation (*F*_ST_) between wild and domesticated peaches (~ 0.76) (Fig. 1f), positive mean Tajima’s *D* values, and slow linkage disequilibrium (LD) decay (half LD decay distance of ~ 35 kb) in domesticated peach are consistent with a narrow domestication bottleneck (Fig. 1g). In contrast, most of the genetic diversity was retained during peach improvement from landraces (1.2 × 10^− 3^) to improved cultivars (1.0 × 10^− 3^), suggesting a weaker improvement bottleneck (*π*_landrace_/*π*_improvement_ = 1.20) in peach (Fig. 1f).

To better understand the basis of the narrow bottleneck during domestication, we examined factors that might affect bottleneck intensity, such as effective population size of founders, length of juvenile phase, mating system, or single versus multiple domestication origins. The effective population size of the founders estimated by the δaδi program [21] was ~ 200 for peach at domestication, which is markedly lower than the corresponding estimates in maize [22] (~ 150,000) and rice [23] (~ 1300). Peach typically is self-compatible with a relative short juvenile phase of ~ 3 years, which is shorter than apple (> 5 years) and pear (> 5 years), and equal to that of grape. Multiple origins of cultivated crops may lead to a mild bottleneck, such as has been reported for grape [24], which contrasts with our previous finding that peach had a single origin and a linear evolutionary route [11]. In addition, the use of grafting promoted the formation of a marked bottleneck as it resulted in the propagation of a limited number of highly interesting genotypes. We conclude that the combined effects of these factors resulted in the narrow domestication bottleneck in peach.

We found that eastern (*π* = 1.1 × 10^− 3^) and western improved cultivars (*π* = 0.9 × 10^− 3^) harbored nearly the same low level of genetic diversity, with little genetic differentiation (*F*_ST_ = 0.08) (Fig. 1f), suggesting a narrow genetic background of current cultivated peach varieties. Therefore, trait improvement in peach may be constrained by the limited genetic diversity in commonly used breeding materials, suggesting the potential benefit of introgressing material from wild relatives in future breeding programs.

### Genome-wide selection signatures during domestication and improvement

**Fig. 2:**
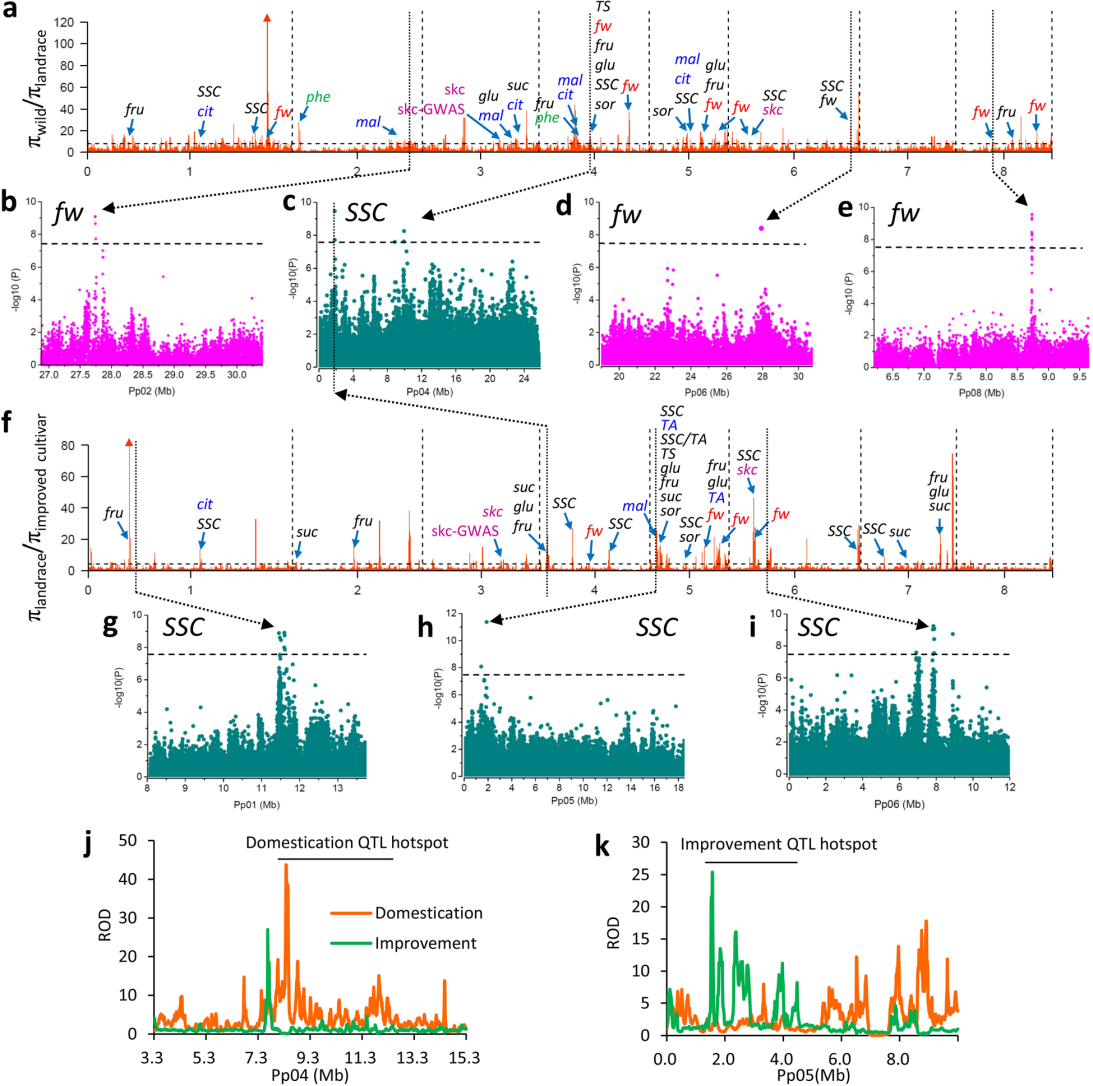
Genome-wide detection and functional annotations of selection sweeps during domestication and improvement. **a**, **f** The genome-wide selective signals associated with domestication (**a**) and improvement (**f**). Putative domestication sweeps (**a**) and improvement sweeps (**f**) are shown as orange bars above the black dashed horizontal threshold line. Blue arrows in (**a**) and (**f**) indicate previously reported QTLs located within the domestication and improvement sweeps. QTL names are listed above the corresponding blue arrows, and QTLs are shown for fruit weight (red), fruit acid (blue), fruit sugar (black), fruit skin color (pink), and total phenolics (green). *fw*, fruit weight; *SSC*, soluble solid content; *sor*, sorbitol content; *TS*, total sugar content; *suc*, sucrose content; *fru*, fructose content; *glu*, glucose content; *TA*, titratable acid content; *mal*, malic acid content; *cit*, citric acid content; *skc*, fruit kin color; *phe*, total levels of phenolic compounds. **b**, **d**, **e** Manhattan plots of GWAS association peaks related to fruit weight on chromosomes 2, 6, and 8 that overlapped with domestication sweeps. The gray horizontal dashed lines in each figure indicate the Bonferroni significance threshold of GWAS (*P* < 3.2 × 10^− 8^). **c**, **g**–**i** Manhattan plot of GWAS association peaks related to fruit SSC on chromosomes 1, 4, 5, and 6 that overlapped with domestication and improvement sweeps. The black horizontal dashed lines in each figure indicate the Bonferroni significance threshold of GWAS (*P* < 2.6 × 10^− 8^). **j** Fruit taste-related QTL hotspots in domestication sweeps. **k** Fruit taste-related QTL hotspots in improvement sweeps

To identify the potential selection signatures of peach domestication and improvement, we screened for genomic regions with a sharp reduction of nucleotide diversity (ROD) between wild and landrace groups (*π*_wild_/*π*_landrace_; domestication) as well as landrace and improved groups (*π*_landrace_/*π*_improvement_; improvement). Consistent with the narrow domestication bottleneck and weak improvement bottleneck, we observed a significantly higher ROD value during domestication (average *π*_wild_/*π*_landrace_ = 3.33) than improvement (average *π*_wild_/*π*_landrace_ = 1.76) (*P* < 0.01) (Fig. 2a, f). The top 5% of the genomic windows or regions with unusually high ROD values in each comparison were defined as selective sweeps. Finally, a total of 142 domestication sweeps and 104 improvement sweeps, covering 11.1% (25.2 Mb) and 9.4% (21.3 Mb) of the assembled genome, harboring 3683 and 3039 genes, respectively, were identified (Fig. 2a, f; Additional file 1: Tables S8-S11). Taken together, we concluded that approximately 18.6% (42.4 Mb) of the peach genome has been shaped by these two selection steps involving domestication from wild relatives. Notably, we found that 15.9% (4.1 Mb; 1.8% of the genome) of domestication sweeps, harboring 768 genes, overlapped with improvement sweeps, indicating that a subset of domestication loci may have undergone a second round of artificial selection for continued improvement of important agronomic traits (Additional file 2: Table S12).

### Fruit size was a major target of selection during domestication

**Fig. 3:**
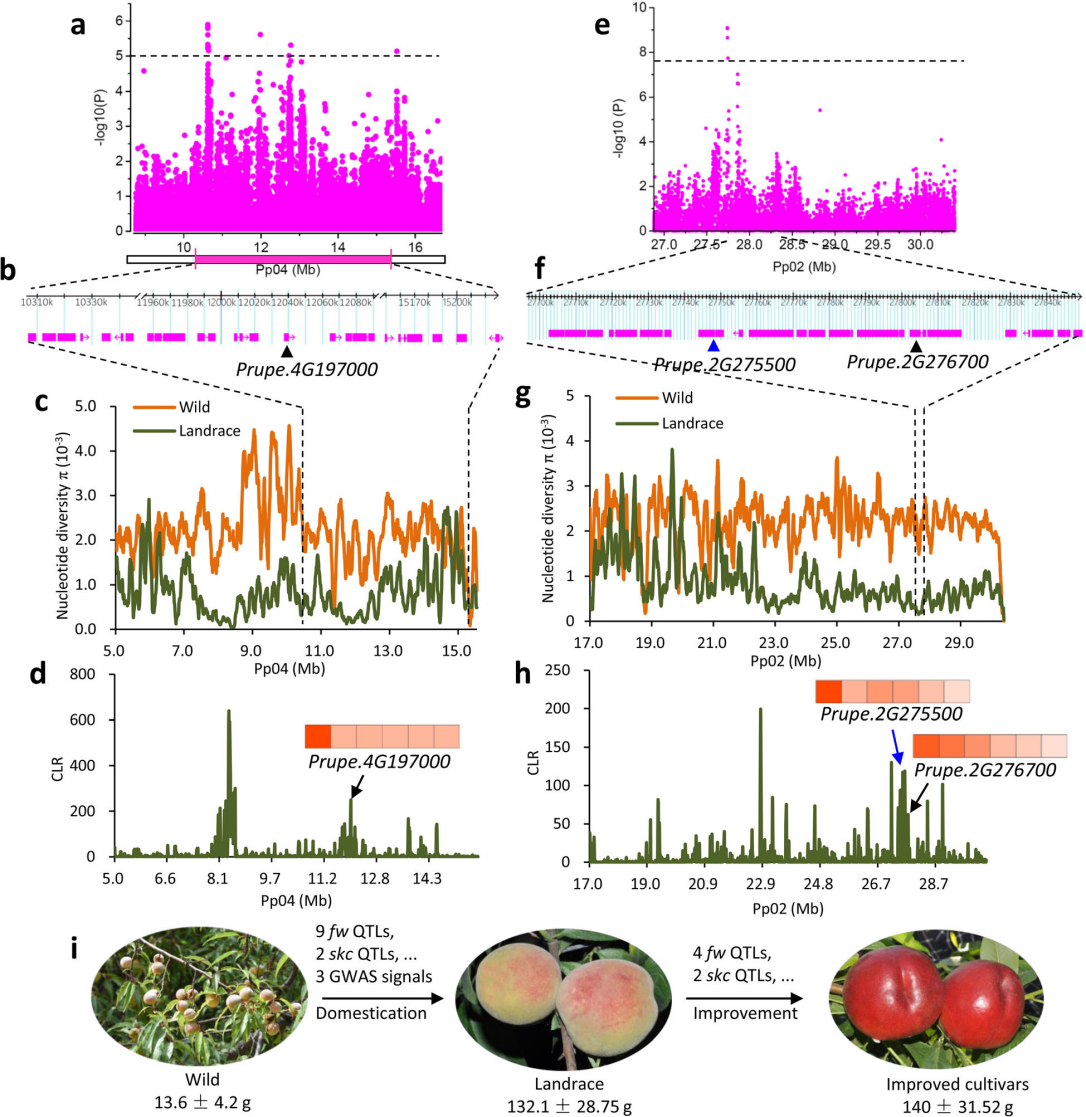
Evolution of fruit size during domestication and improvement in peach. **a**–**d** Selection on the *fw* QTL on chromosome 4 during peach domestication. **a** Regional Manhattan plot of GWAS for fruit size and the corresponding co-located QTL related to fruit size on chromosome 4. Although no signal exceeded the Bonferroni significance threshold of GWAS (*P* < 3.2 × 10^− 8^), a secondary GWAS signal was identified (*P* < 1× 10^− 5^). **b** Gene models in the fruit size related QTL on chromosome 4. The candidate gene, *Prupe.4G197000*, is labeled with a black triangle. **c** Nucleotide diversity (*π*) of the interval of the fruit size related QTL. **d** CLR value of the interval of the fruit size related QTL. A heat map of the expression level of *Prupe.4G197000* during different stages of fruit development (20, 40, 60, 80, 100, and 120 days after bloom from left to right) is shown. **e**–**h** Selection on the fruit weight GWAS signal on chromosome 2 during domestication. **e** Regional Manhattan plot of GWAS for fruit size on bottom of chromosome 2. The gray horizontal dashed line indicates the Bonferroni significance threshold of GWAS (*P* < 3.2 × 10^− 8^). **f** Gene models in the fruit size QTL region on chromosome 2. Candidate genes, *Prupe.4G275500* and *Prupe.2G276700*, are indicated with blue and black triangles, respectively. **g** Nucleotide diversity (*π*) of the fruit size QTL region. The selection for big fruit size has led to the hitchhiking of a nearly 10-Mb region on the bottom of chromosome 2. **h** CLR value of the fruit size QTL region. The heat map shows gene expression levels of *Prupe.4G275500* and *Prupe.2G276700*. **i** Schematic diagram of the two-step evolution of peach fruit size. The change of fruit appearance and related QTL (*skc*) is also shown. QTLs and GWAS signals that were putatively selected during domestication and improvement are indicated

Increase in fruit or seed size is a key target in the breeding history of most crop species. The peach fruit size has increased more than 10 times and undergone two rounds of selection, from 13.6 ± 4.2 g in the wild group to 132.1 ± 28.75 g in landraces and 140 ± 31.52 g in improved cultivars (Fig. 3i, Additional file 3: Figure S6). We hypothesized that fruit size may have been the predominant target of selection during domestication, with a mild selection during the subsequent phase of peach improvement. Previous studies have identified multiple quantitative trait loci (QTLs) associated with fruit size (*fw*) in peach [25–28], but the genetic basis of the increase of fruit size during domestication and improvement remains largely unknown.

We compared the selective sweeps with the locations of known QTLs. A total of nine known *fw* QTLs on chromosomes 1, 4, 6, and 8 fell within domestication sweeps, including a stable QTL on chromosome 4, identified in a multi-year mapping experiment [28] (Fig. 2a). Selections for these nine QTLs likely contributed to the enlargement of peach fruit size during the evolutionary transition from wild to landrace accessions (Fig. 3i). For instance, the stable *fw* QTL on chromosome 4 (10.3–15.2 Mb) showed sharp ROD and high composite likelihood ratio (CLR) values corresponding to a strong domestication sweep (Fig. 3a, c, d). Moreover, a SNP associated with fruit weight (*P* = 7.5 × 10^− 7^) within this QTL was also identified in the GWAS analysis (see below; Fig. 3a). Within this QTL, we found one gene (*Prupe.4G197000*) encoding a putative auxin-responsive GH3-like protein (Fig. 3b), which showed high expression at the early stage of fruit development (Fig. 3d). Notably, a homolog of this gene has been proposed as a candidate for the increase in fruit mass during tomato domestication [5]. Compared with domestication sweeps, only four *fw* QTLs, located on chromosomes 4, 5, and 6, overlapped with improvement sweeps (Fig. 2f), which may explain the relatively small increase in fruit size during improvement (Fig. 3i, Additional file 3: Figure S6). Collectively, the difference in the number of *fw* QTLs associated with the domestication and improvement sweeps (Fig. 2a, f) suggests a stronger selection for fruit size during domestication than improvement.

To further track the selection for fruit size during the two selection steps, we performed GWAS analysis of fruit weight (Additional file 3: Figure S7). A total of eight association peaks, on chromosomes 1, 2, 3, 4, 6, 7, and 8 (*P* < 3.2 × 10^− 8^), were identified, of which three overlapped with known QTLs and five were new (Additional file 2: Table S13, Additional file 3: Figure S7). Among the association peaks, three (chromosomes 2, 6, and 8) overlapped with domestication sweeps, while no peak was found in the improvement sweeps (Fig. 2a, b, d, e). Specifically, we detected 52 SNPs significantly associated with fruit weight, of which 26 underwent selection during domestication, while only two showed selection signals during improvement (Additional file 2: Table S13). This finding is congruent with stronger selection for fruit size during domestication than improvement. Notably, we found a strong association on chromosome 2 overlapping with a domestication sweep, supported by high ROD and CLR values (Fig. 3e, g, h). Two candidate genes within this region, *Prupe.2G275500* and *Prupe.2G276700*, are related to cell division, with high expression at the cell division stage of fruit development (20 days after full bloom) (Fig. 3f, h). The latter encodes a PLAC 8 family protein and is homologous to *fw2.2* from tomato, which controls fruit size through regulating the cell number [29] (Fig. 3f). Selection on this QTL may contribute to the increase in fruit size during domestication and have resulted in extremely low genetic diversity of a nearly 10-Mb region on the bottom of chromosome 2 (Fig. 3g, h) (*π* = 0.573 × 10^− 3^ in the region versus 1.2 × 10^− 3^ in the whole genome), reflecting hitchhiking effects (LD decay was ~ 90 kb in this region versus ~ 35 kb in the whole genome) (Additional file 3: Figure S8).

### Loss of bitterness and improvement in fruit taste during domestication and improvement

Fruit taste is another important target for artificial selection in fruit crops. Wild peaches taste bitter, landraces less so, while improved cultivars have no bitterness and a high sugar content, suggesting that fruit taste was successively selected for during both domestication and improvement. Fruit taste is mainly related to sugar content, acid content, and the balance between them. Significant changes in the content of these carbohydrates during peach evolution have been observed [30] (Additional file 3: Figures S6 and S9) and, accordingly, a series of fruit sugar- and acid-related QTLs [25–28, 31, 32] were found to be co-located with domestication and/or improvement sweeps (Fig. 2a, f). Moreover, some of these QTLs were situated in genomic regions that have undergone two rounds of selection (Additional file 2: Table S12), further suggesting successive selection for fruit taste during domestication and subsequent improvement.

**Fig. 4:**
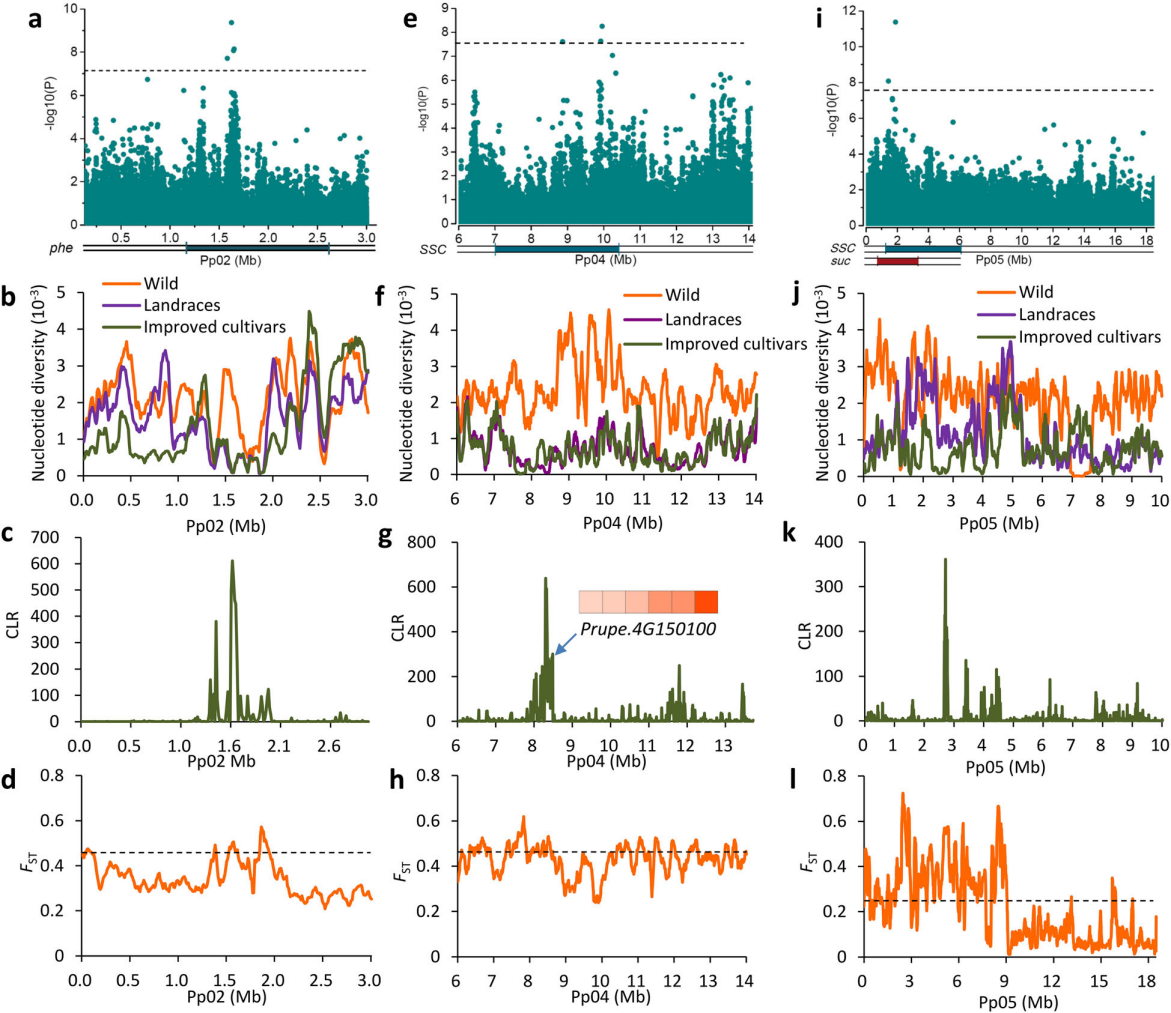
Selective sweeps underlying the changes in fruit taste during domestication and improvement. **a**–**d** Selection on the *phe* QTL during peach domestication. **a** Regional Manhattan plot of an association peak for levels of total phenolic compounds on chromosome 2. The gray horizontal dashed line indicates the Bonferroni significance threshold of GWAS (*P* < 6.5 × 10^− 8^). Previous reported *phe* QTL is highlighted under the Manhattan plot. The selective signals on the genomic regions harboring the *phe* QTL are supported by sharp ROD (**b**), high CLR (**c**), and high *F*_ST_ (**d**). **e**–**h** Selection on a *SSC* QTL on chromosome 4 during domestication. **e** Regional Manhattan plot of an association peak for SSC. The gray horizontal dashed line indicates the Bonferroni significance threshold of GWAS (*P* < 2.6 × 10^− 8^). Previous reported *SSC* QTL is highlighted. The selective signals on the genomic regions harboring *SSC* QTL are supported by sharp ROD (**f**), high CLR (**g**), and high *F*_ST_ (**h**). A heat map of the expression level of a candidate gene, *Prupe.4G197000*, during different stages of fruit development (20, 40, 60, 80, 100, and 120 days after bloom from left to right) is shown in **g**. **i**–**l** Selection on a *SSC* QTL on chromosome 5 during improvement. **i** Regional Manhattan plot of an association peak for SSC. The gray horizontal dashed line indicates the Bonferroni significance threshold of GWAS (*P* < 2.6 × 10^− 8^). Previously mapped *SSC* and *suc* QTLs are highlighted. The selective signals on the genomic regions harboring *SSC* QTL are supported by sharp ROD (**j**), high CLR (**k**), and high *F*_ST_ (**l**). The horizontal dashed lines in (**d**), (**h**), and (**l**) indicates the 5% cut off of *F*_ST_

A total of 27 fruit taste-related QTLs were found in the domestication sweeps (Fig. 2a). A previous study reported that peach fruit bitterness is conferred by levels of phenolic compounds [33], and we found that fruit produced by wild peach accessions had a significantly (*P* < 0.01) higher total phenolic content (681.8 ± 102.4 mg/kg) than fruit of the landraces (141.8 ± 118.1 mg/kg) and improved cultivars (96.0 ± 93.7 mg/kg). Previous studies identified several QTLs for total phenolic levels (*phe*) [28], of which two on chromosomes 2 and 4 showed strong domestication signals (Fig. 2a and Fig. 4a–d). We propose that selection for these two QTLs underlies the loss of flesh bitterness during peach domestication (Fig. 2a and Fig. 4a–d). We also identified a GWAS signal for total phenolic content within the *phe* QTL on chromosome 2 (Fig. 4a; Additional file 3: Figure S10). Moreover, the most significant signal (*P* = 4.31 × 10^− 10^) in this association peak showed considerable differentiation between wild and landrace accessions (*F*_ST_ = 0.98) and was almost fixed in landraces (98.5%), suggesting that the genotype conferring bitter fruit was swept during domestication, consistent with the phenotype.

We also identified 31 QTLs within the improvement sweeps that may have contributed to the second-round improvement of fruit taste (Fig. 2f). One of these, *Prupe.4G037800*, encoding a sugar transporter within a *suc* QTL [26], overlapped with the GWAS signal of fruit soluble solid content (SSC) on chromosome 4 (Fig. 2c, f). This gene was differentially expressed during fruit development with high expression at the mature stage (Additional file 3: Figure S11), suggesting its contributions to the increase of fruit taste during improvement. Notably, we found that the improvement sweeps were associated with a greater number of sugar-related QTLs (22 QTLs) than the domestication sweeps (18 QTLs) (Fig. 2a, f), suggesting more specific breeding for better taste and high sweetness in improvement than in domestication. In contrast, more fruit acid-related QTLs (8) were found in the domestication sweeps than in the improvement sweeps (4) (Fig. 2a, f), indicating a selection for reduced fruit acidity during domestication, which is typical of domesticated peach (Additional file 3: Figure S9).

To further characterize the selection for fruit taste, we performed GWAS analysis of SSC (Additional file 3: Figure S7). A total of 35 association SNPs and 10 association peaks were identified (*P* < 2.6 × 10^− 8^), of which three overlapped with known QTLs and seven were novel (Additional file 2: Table S13, Additional file 3: Figure S7). Among the association peaks, one on chromosome 4 fell within the domestication sweeps and four (chromosomes 1, 4, 5, and 6) were located within the improvement sweeps (Fig. 2a, c, f–i). Moreover, many more SSC associations were identified in the improvement sweeps than in the domestication sweeps, consistent with stronger selection for fruit taste during improvement than domestication.

The GWAS signal on chromosome 4 co-located with a previously reported SSC QTL [28] (Fig. 4e) and the genomic region harboring this QTL showed a strong domestication signal, supported by a sharp ROD value, a high CLR value, and a high *F*_ST_ between wild and landrace accessions (Fig. 4f–h). Through combining the transcriptome and biochemical analyses, we identified a candidate gene, *Prupe.4G150100*, for SSC within this QTL (Fig. 4g, Additional file 3: Figure S12). This gene, encoding a putative nine-*cis*-epoxycarotenoid dioxygenase 3 protein (NCED3) and involved in the abscisic acid (ABA) signaling pathway that play essential roles in fruit ripening [34], showed a large increase in expression at the ripening stage (~ 200 times), and its expression profiles during fruit development were strongly correlated with both SSC and ABA contents (Fig. 4g, Additional file 3: Figure S12). We found that the genomic region of the *NCED3* gene showed very low nucleotide diversity in the landrace group (*π* = 0.688 × 10^− 3^ in this region versus 1.2 × 10^− 3^ in the whole genome), and that the selection for high SSC was likely resulted from the hitchhiking of an approximately 5-Mb genomic region (LD decay was ~ 150 kb in this region versus ~ 35 kb in the whole genome) (Fig. 4f) (Additional file 3: Figure S12). Another strong SSC association was identified on the top of chromosome 5, located within the overlap between *SSC* and *suc* QTLs [28] (Fig. 4i). The high ROD and CLR values from the comparison of landraces and improved cultivars suggested a strong improvement signal in this QTL (Fig. 4j, k). Moreover, an extremely high *F*_ST_ value of the 9-Mb region at the top of chromosome 5 further supported a strong selection for this QTL during improvement (Fig. 4l).

**Fig. 5:**
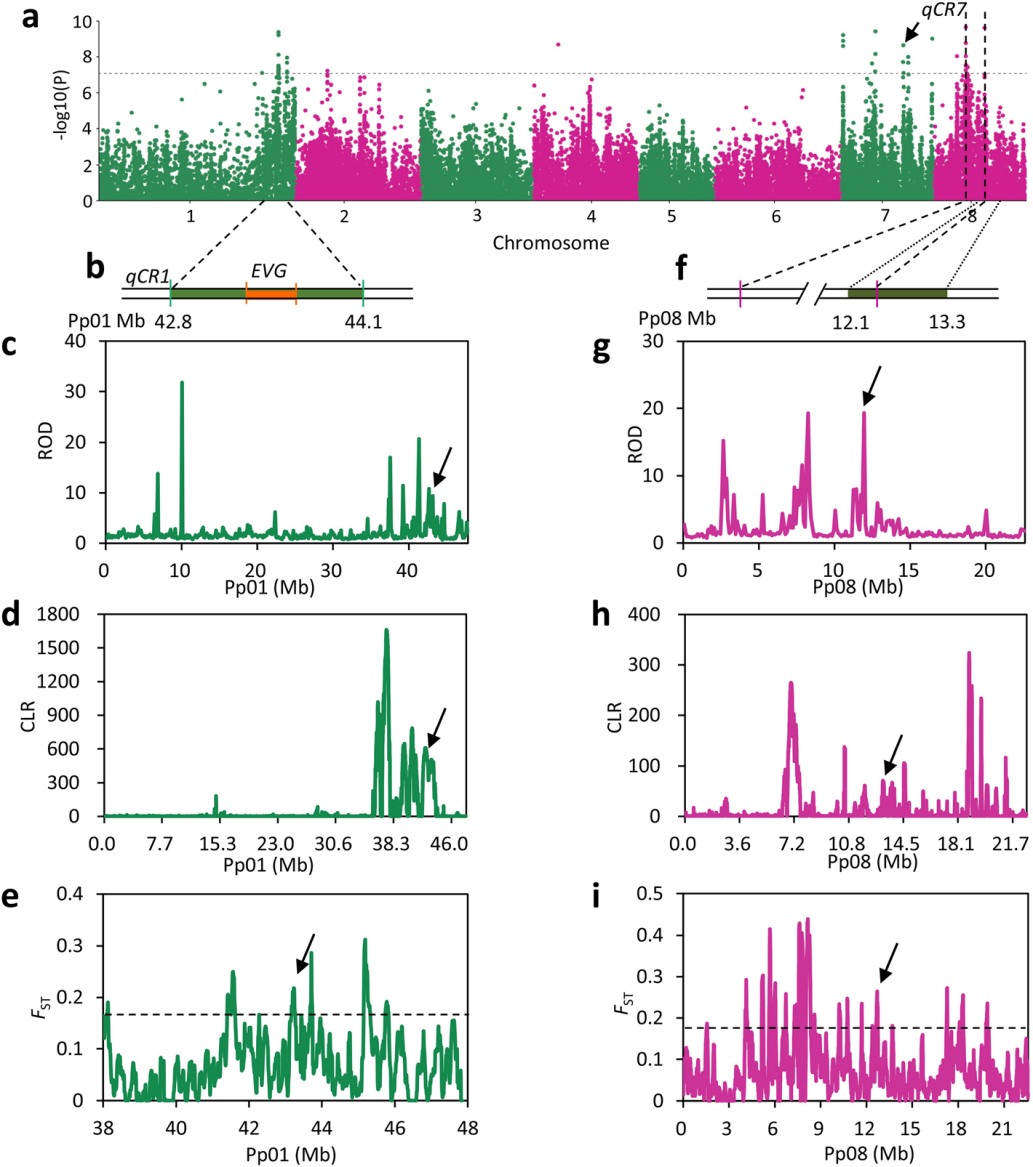
Selection targets for low CR breeding. **a** Manhattan plot of GWAS for CR. The gray horizontal dashed line indicates the Bonferroni significance threshold of GWAS (*P* < 4.1 × 10^− 8^). **b** Physical location of CR QTL and the *EVG* locus on chromosome 1. The green and orange rectangles represent CR QTL and the *EVG* locus, respectively. **c**–**d** Distribution of ROD (**c**) and CLR (**d**) values across chromosome 1 using 100-kb non-overlapping sliding windows. The CR QTLs are pointed by black arrows. **e** Distribution of *F*_ST_ values between landrace and low CR improved cultivars in the *qCR1* genomic region. The black horizontal dashed line indicates the top 5% of genomic regions with highest *F*_ST_. **f** Physical location of the CR QTL on chromosome 8. The green rectangle represents previous CR QTL. **g**–**h** Distribution of ROD (**g**) and CLR (**h**) values across chromosome 8 using 100-kb non-overlapping sliding windows. The CR QTLs are pointed by black arrows. **i** Distribution of *F*_ST_ values between landrace and low CR improved cultivars in the *qCR8* genomic region. The gray horizontal dashed line indicates the top 5% of genomic regions with highest *F*_ST_

We found that QTL hotspots [35] related to fruit taste differed in the domestication and improvement phases and were located on chromosome 4 (7.5–13.0 Mb) (Fig. 2j) and 5 (1.5–4.5 Mb) (Fig. 2k), respectively. We also observed that 11 scattered QTLs were shared by domestication and improvement sweeps (Fig. 2a, f). In addition, two QTLs [17, 32] and one GWAS signal related to fruit skin color (*skc*) fell within both the domestication and improvement sweeps, suggesting a successive selection for fruit appearance during the two selection steps (Fig. 2a, f and Fig. 3i).

### Selected regions relevant to low chilling requirement breeding

In addition to fruit size and taste, selection for low chilling requirement (CR) is a unique breeding objective for peach, and contributes to extension of the harvest season and adaptation to low-chill zones. Moreover, global warming has already led to a significant decrease in winter chill, encouraging breeders to select low CR cultivars that are likely to perform well in future climates [36]. The continuing efforts by breeders to select for lower CR have changed the pattern of CR distribution, as evidenced by the fact that 90% of peach cultivars required more than 800 chilling hours to break dormancy 60 years ago, whereas now only 20% of cultivars require this much chilling [37]. Although a few previous studies have identified multiple QTLs associated with peach CR [38, 39], the genome-wide genetic basis underlying low CR breeding has not yet been determined. To address this, we performed GWAS for CR (Additional file 3: Figure S13) and identified seven association peaks, located on chromosomes 1, 3, 7, and 8 (−log(*P*) > 7.59) (Fig. 5a, Additional file 2: Table S14). Of these, three were within or close to previously reported CR QTLs [38, 39], while the other four were new (Fig. 5a, b, f). Notably, the strong association peak (*P* = 3.14 × 10^− 14^) on chromosome 1 overlapped with a known major CR QTL (*qCR1a*) that explained up to 44.8% of the phenotypic variance [38] (Fig. 5a, b). Moreover, this strong association co-localized with the *EVG* locus underlying the *evergrowing* peach dormancy mutation (*evg*) [40, 41] (Fig. 5b). Six tandemly arranged *Dormancy Associated MADS-box* transcription factors (*DAM1-6*; *Prupe.1G531100*, *Prupe.1G531300*, *Prupe.1G531400*, *Prupe.1G531500*, *Prupe.1G531600*, *Prupe.1G531700*) have been identified in *qCR1a*, with a 41.7-kb deletion spanning all or part of *DAM1-4*, conferring a non-dormancy (or CR = 0) phenotype in *evg* peach [41].

Next, we identified a total of 112 targets of low CR breeding selection by screening for genomic regions with high ROD and CLR values, using a 5% cutoff (Additional file 1: Table S15). Two association peaks from the CR GWAS that overlapped with previously reported QTLs [40, 41] on chromosomes 1 and 8 fell within the selection targets (Fig. 5c, d, g, h), suggesting that these two QTLs may underlie the signatures of breeding for low CR cultivars. High *F*_ST_ values further supported the selection for these QTLs (Fig. 5e, i). Furthermore, we also obtained strong association signals within these two QTLs on chromosomes 1 and 8 by performing another GWAS through classification of accessions into low CR and non-low CR (Additional file 3: Figure S14, Additional file 1: Table S16). Selection for these two QTLs could have contributed to the extension of a suitable planting area for peach to low-altitude zones. A total of seven *SHORT VEGETATIVE PHASE* (*SVP*) subfamily genes related to flowering time was identified within these two QTLs, including the six *DAM* genes in the *EVG* locus and one (*Prupe.8G069300*) in *qCR8* on chromosome 8. Interestingly, the CR QTL on chromosome 1 also harbored the loci associated with bloom date and heat requirement [38]. Moreover, overexpression of peach *SVP* gene (*Prupe.8G069300*) within the interval of the GWAS signal on chromosome 8 in a model plant, *Arabidopsis thaliana*, led to the strong vegetative growth and delayed flowering time (Additional file 3: Figure S15), providing strong evidence for candidate genes for CR in peach. These results will be valuable for the development of markers for low CR breeding and the identification of genes responsible for the CR. A PCR-based marker with an accuracy of 92.2% has been developed for low CR peach identification (Additional file 3: Figure S16).

### Divergent selection and local breeding

**Fig. 6:**
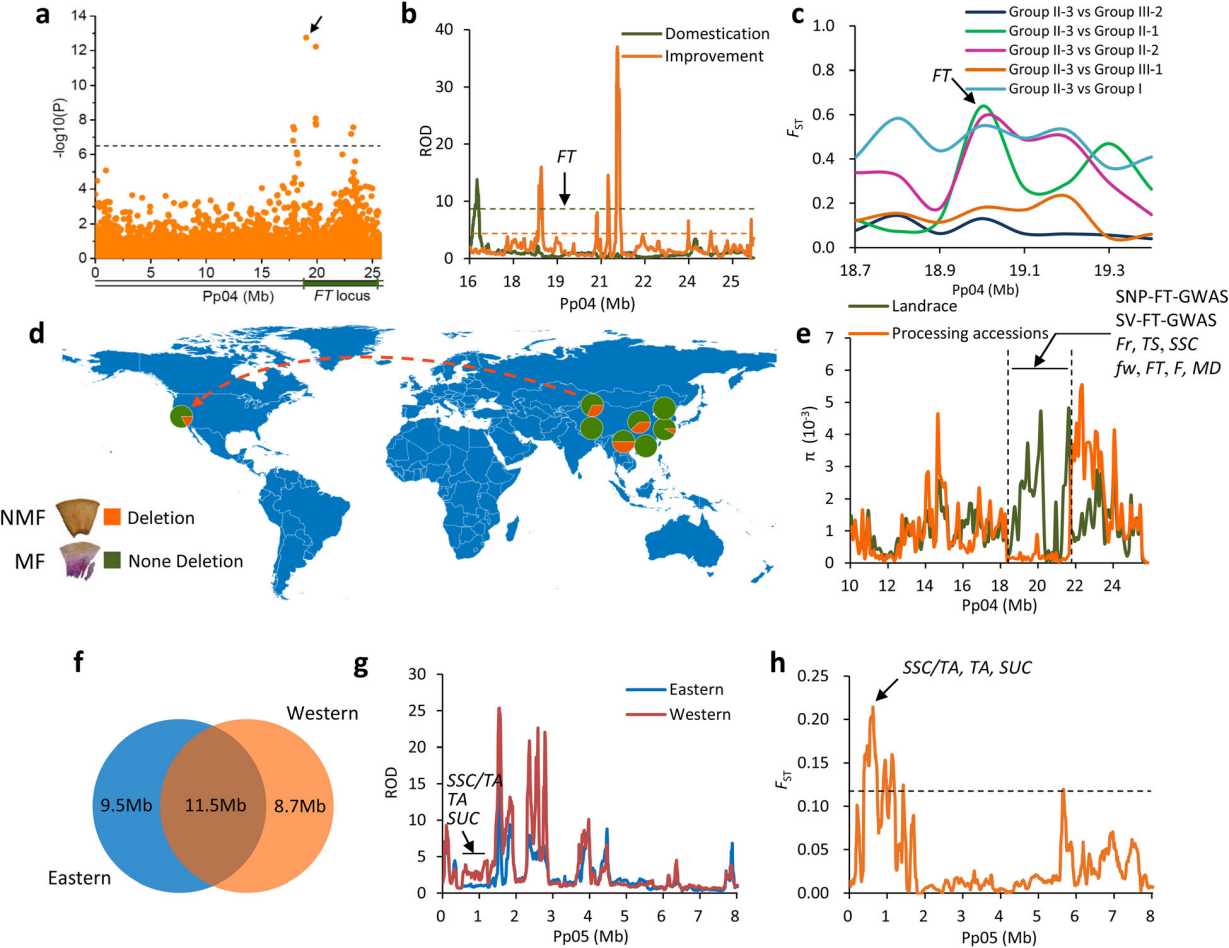
Divergent selection on flesh texture and local breeding. **a** Regional Manhattan plots of GWAS for flesh texture on chromosome 4. The top signal of GWAS was highlighted using a black arrow. **b** Distribution of ROD values during domestication and improvement in the association region. The green and orange horizontal dashed lines indicate 5% cut off of ROD value during domestication and improvement, respectively. **c** Distribution of *F*_ST_ values between different groups in the association region. The *FT* QTL was highlighted using a black arrow. **d** Geographical pattern of the allele frequencies at the causal polymorphisms (top signal of GWAS). The orange dashed line with arrow represents the putative spread route of peaches with non-melting fruit flesh. MF and NMF represent melting and non-melting flesh texture, respectively. **e** Comparison of nucleotide diversity of between landraces and processing peach cultivars. A nearly 4-Mb strong selective sweep (between two dashed vertical bars) was highlighted and annotated. The QTLs and GWAS signals in this region are indicated. *Fr*, fruit firmness; *FT*, flesh texture; *F*, flesh adhesion; *MD*, maturity date. **f** Venn diagram showing sizes of shared and unique genome regions under selection in eastern and western improved cultivars. **g** Distribution of ROD values of eastern and western improved cultivars at the top of chromosome 5. Three QTLs, including *SSC/TA*, *TA*, and *suc*, in western-specific selective sweeps are labeled. **h** Distribution of *F*_ST_ between eastern and western improved cultivars at the top of chromosome 5. Three QTLs, including *SSC/TA*, *TA*, and *suc*, in western-specific selective sweeps are labeled. The horizontal dashed line indicates the 5% cut off

Fruit texture is an important agronomic trait for peach that is directly related to mouth-feel, transportation tolerance, and processing quality. We found that fruit texture showed geographic distribution patterns, with all wild accessions showing a melting fruit texture, while landraces and improved cultivars have both melting and non-melting phenotypes. Most of the landraces from northeastern and southern China, as well as the middle and lower reaches of the Yangtse river, showed a melting fruit texture, while most of the landraces harboring a non-melting phenotype were from northwestern China, the Yungui Plateau, and the northern Plain of China. Western and eastern improved cultivars showed both melting and non-melting fruit textures. We therefore concluded that genes underlying fruit texture underwent divergent selection during geographic differentiation or local breeding. Using GWAS, we identified a 70.5-kb SV (Pp04: 19,026,186) associated with fruit texture within the previously mapped *FT* locus [14, 16] (Fig. 6a, Additional file 3: Figure S2 and S3). Although a non-melting fruit texture may have arisen during domestication or post-domestication, we only identified weak domestication and improvement signals at the association region (Fig. 6b). However, using *F*_ST_ analysis of pairwise population differentiation, we detected a strong signal at the *FT* locus in accessions from northwestern China versus the northeastern, southern, and Yangtze regions (Fig. 6c). Further investigation of allele frequencies suggested that the mutant allele was mainly distributed in northwestern China, the Yungui Plateau, the North Plain, and in western improved cultivars, which is consistent with the fruit texture distribution (Fig. 6d). These data, combined with the phylogenetic analysis, suggest that the non-melting alleles in western improved cultivars may be derived from northwestern China through the ancient Silk road and subsequently spread to other western countries (Fig. 1b and Fig. 6d).

In addition to breeding for fresh consumption, breeding for processing into peach can or juice is an important objective during improvement, and the non-melting fruit texture is an essential selection target for breeding of processing peach [42]. A total of 15 processing peach cultivars were sequenced in this study (Additional file 1: Table S1). Through scanning for genomic regions with high ROD in comparison of landraces and these 15 processing peach cultivars, we found a nearly 4-Mb selective sweep around the fruit texture GWAS signal on chromosome 4 (Fig. 6e). Intriguingly, this selection signal also harbored QTLs related to SSC, fruit firmness, stone adhesion, total sugar, maturity date [32], and fruit weight (Fig. 6e). Some of these traits (non-melting, high fruit firmness, and clingstone) are fundamental selection goals for processing peaches [42], and this 4-Mb segment may therefore represent a major genomic signature for selection of processing peaches.

Peach fruits with both high sugar and titratable acidity (TA) are widely consumed in western countries, while those with only high sugar are popular in eastern countries, suggesting that breeding targets between eastern and western countries may differ. To test this hypothesis, we determined improvement sweeps for eastern and western improved groups, respectively, and identified a total of 102 and 87 selective sweeps (Additional file 1: Tables S17 and S18, Additional file 3: Figure S17). Only approximately half of the improvement sweeps in the eastern group fell within the top 5% of the ROD regions in the western group (Fig. 6f), suggesting a limited number of shared breeding targets between eastern and western accessions, which may underlie the difference in fruit taste between different areas (Additional file 1: Tables S17 and S18, Additional file 2: Table S19-S21). One example is the SSC:TA ratio, which is essential for fruit taste, for which we found a major 820-kb QTL [26] overlapping with a western-specific sweep on chromosome 5 (Fig. 6g), which could have contributed to the selection for high SSC and TA in western cultivars. The local selection for this QTL was also supported by a high *F*_ST_ value, when western and eastern accessions were compared (Fig. 6h). We conclude that differential selection for these regions conferred the richer flavor in western cultivars than eastern cultivars. Flavor selection involves multiple factors and it is notable that the increase of sugar content is not always accompanied by a parallel increase in consumer preference and flavor [10, 43]. An alternative explanation for different selection targets is that the western and eastern peach accessions have undergone different selection pressures resulting from the distinct environment.

## Conclusions

In this study, we generated a large genome-variation dataset for peach by sequencing 480 widely collected wild and cultivated accessions from around the world. Using this resource, we tracked the genetic changes and selection targets during domestication and breeding, providing insights into the history of peach evolution. We found that fruit size was predominantly selected during domestication and that selection for large fruits has led to the loss of genetic diversity in *fw* QTLs. Fruit taste-related QTLs were successively selected for by two successive selection steps, and many more fruit taste-related QTLs were found to be associated with improvement than with domestication, suggesting a sharp decline of genetic diversity at loci related to fruit taste. Although peach fruit taste has improved in modern cultivars compared with their wild progenitors, successive selection for sugar and acid may lead to a homogenization and deterioration in flavor quality, similar to the case of commercial tomato varieties [43]. In this regard, the use of landrace accessions as parents, especially heirloom varieties, has considerable potential in introgressing valuable genetic diversity.

Our variation dataset provides a valuable resource for future peach improvement using novel breeding technologies and strategies, such as genomic selection, molecular design breeding, and introgression of novel alleles from landraces and wild relatives [44]. Associations for 11 agronomic traits also provide valuable information to accelerate genetic gains for key traits to improve yield and edible quality in peach.

## Methods

### Plant material and sequencing

We sampled a total of 480 peach accessions from the National Peach Germplasm Repository of China (NPGRC) (Zhengzhou) (N 34.71°, E 113.70°, A.S.L. 74 m), including 52 wild relatives (45 of *Prunus mira* Koehne, four of *P. davidiana* (Carr.) Franch., two of *P. kansuensis* Rehd., a single sample of *P. potaninii* Batal), 213 landrace accessions (*P. persica* L.), and 215 improvement accessions (*P. persica* L.) (Additional file 1: Table S1). Genomic DNA was extracted from young leaves using the cetyltrimethylammonium bromide (CTAB) method [45] and at least 4 μg of genomic DNA from each accession was used to construct a sequencing library, following the manufacturer’s instructions (Illumina Inc.). Paired-end sequencing libraries with an insert size of approximately 300 bp or 500 bp were sequenced using an Illumina GAII or Hiseq 2500 sequencer, with read lengths of 49 bp, 90 bp, or 125 bp. In total, more than 1 Gb of sequence data was generated for each accession (Additional file 1: Table S1).

### Alignment and variation calling

#### Mapping of short reads

Paired-end sequencing reads from each accession were mapped to the peach “Lovell” genome [12] (release version 2.0_a2.1) using BWA [46] (version 0.7.12), with the following parameters: bwa mem -t 4 -M -R. Mapped reads in SAM format were converted into BAM format, sorted according to mapping coordinates, and processed for PCR duplicate removal using the Picard package (http://broadinstitute.github.io/picard/, version 1.136) with default parameters. The coverage and depth of sequence alignments were computed using the DepthOfCoverage program in the Genome Analysis Toolkit [47] (GATK, version 3.4-46) and the genomencov program in BEDtools [48] (version 2.24.0), respectively. The coverage and depth of each accession are provided in Additional file 1: Table S1.

#### SNP calling

SNP calling followed GATK Best-Practices (https://software.broadinstitute.org/gatk/best-practices/bp_3step.php?case=GermShortWGS). To obtain accurate SNPs, we filtered low-quality alignments with a mapping quality < 20 using SAMtools [49] (V1.3.1). SNP detection was performed using the GATK HaplotypeCaller [50], which calls SNPs via local de novo assembly of haplotypes in an active region. The detailed processes were as follows: (1) after filtering out the low-quality alignments, the reads around the INDELs were realigned in two steps, including identifying regions where realignment was needed using the GATK RealignerTargetCreator package and realigning the regions found in the first step using the GATK IndelRealigner package. Next, a realigned BAM file for each accession, which was used for SNP detection, was generated using the GATK PrintReads packages. (2) SNPs were detected at a population level using the realigned BAM file with GATK HaplotypeCaller. To reduce false positives, we set a high SNP confidence score with the following parameters: -stand_call_conf 30 -stand_emit_conf 40. (3) To ensure the quality of variant calling, we supplied a hard filter for the raw SNPs with SNP quality > 40 and more than two supporting reads using GTAK VariantFiltration with the following parameters: QUAL < 40, QD < 2.0, FS > 60.0, MQ < 40.0, MQRankSum < − 12.5, ReadPosRankSum < − 8.0. To further reduce false positives, the SNP number per 10 bp was limited to three using the following settings: --clusterWindowSize 10, --clusterSize 3.

#### INDEL calling

INDEL calling was performed using the same pipeline as SNP calling since GATK is capable of calling SNPs and INDELs simultaneously. To reduce false positives, we also supplied a harder filter for raw INDELs using GTAK VariantFiltration with the following parameters: QD < 2.0, FS > 200.0, ReadPosRankSum < − 20.0. Insertions and deletions ≤ 5 bp were defined as small INDELs.

#### SV calling

To reduce the false positives, SV calling was performed using three programs, SpeedSeq (version 0.1.2) [51], DELLY (version 0.7.8) [52], and manta (version 1.4.0) [53]. For SpeedSeq, paired-end reads were mapped to the reference genome using the aln module in SpeedSeq with the following parameters: speedseq align -R -t 4. Three BAM files were generated, including a full, duplicate-marked, sorted BAM, a BAM file containing split reads, and a BAM file containing discordant read-pairs. SVs were identified using the sv module in SpeedSeq using the following settings: sv -o -x -t 25 -R -B -D -S -g -P. Finally, the SV file in VCF format was generated. For DELLY, mapped pair-end reads in BAM format after sorting and marking PCR duplicates were used as input. SVs were identified by DELLY with default parameters. SV files in VCF format for all of 480 samples were merged into a population-level VCF file using bcftools. For manta, the same BAM files with DELLY were used to detect SVs using configManta.py with default parameters. SV files for 480 accessions were then merged using SURIVER and genotyping for the accessions were called using SVtyper [51] with default parameters. The shared and private SVs for three callers were detected using the merge module in SURIVAR [54] (Additional file 3: Figure S18). Finally, SVs identified by at least two callers were kept and used for downstream analyses.

#### SNP annotation

SNP annotation was performed based on genomic locations and predicted coding effects according to the peach genome annotation [12] (release annotation version a2.1) using snpEff (version 4.1g) [55]. The final SNPs were categorized in exonic regions, intronic regions, splicing sites, 5′ and 3′ untranslated regions (UTRs), upstream and downstream regions, and intergenic regions. SNPs in the coding sequence were further grouped into synonymous SNPs (which did not cause amino acid changes) and nonsynonymous SNPs (which caused amino acid changes) (Additional file 2: Tables S2 and S3).

### SNP genotype imputation

We noted that 13 accessions exhibited a genotyping miss rate > 30% (Additional file 3: Figure S19). In these cases, we inferred missing genotype data using the hidden Markov model (HMM) in the Beagle software [56] (version 4.0) with the following default parameter settings: unphased and non-reference; iterations = 10 window = 50,000 nthreads = 10.

### Population genetics analysis

#### Phylogeny

A neighbor-joining tree was constructed with all 4,980,259 SNPs using the PHYLIP software (version 3.696) with 100 bootstrap replicates [57], and visualized using the FigTree software (http://tree.bio.ed.ac.uk/software/figtree/, version 1.4.2) and evolview (v2) [58].

#### PCA

We performed the PCA using the GCTA software (version 1.26.0) with default settings [59]. The first three eigenvectors were retained to create a plot in two or three dimensions.

#### Population structure

The population structure was also investigated with the STRUCTURE software [60] (version 1.3.0), which is based on a likelihood model. We ran 10,000 iterations, and the number of clusters (K) was set from 2 to 7, representing the assumed groups of the simulated ancestry population. The best *K* was inferred based on the delta K method using the Structure Harvester program [61].

#### LD analysis

LD was calculated on the basis of SNPs with MAF greater than 0.05 using PLINK software (Version 1.07) [62] with following: --file --*r*2 --ld-window 99999 --ld-window-kb 200 --out. Then, values for the *r*2 statistics were obtained. LD decay was calculated based on *r*2 between two SNPs and the distance between the two SNPs.

### Population size estimation

The effective population size at domestication was estimated using the δaδi program (Version 1.7.0) [21]. We fitted a two-population model with landrace and improved groups mixed together and compared to the wild group (Additional file 3: Figure S20 and Additional file 2: Table S22). The simulation was carried out 10 times, and each time we randomly selected 500,000 SNPs and estimated 95% confidence intervals on the basis of the best fitting parameters. The parameters inferred by δaδi were scaled by 2*N*_e_, with *N*_e_ being the ancestral population size. The ancestral population size was estimated using the formula 4*N*_e_ × *μ* × *L* = *θ*, where *μ* is the mutation rate, *L* is the generation time, and *θ* is the genetic diversity. We used *θ*_wild_ to estimate *θ* (∼ 3.5 × 10^− 3^). A mutation rate of 7.77 × 10^− 9^ (the mutation rate per generation) [63] and a generation time of 3 were used for *μ* and *L*. Therefore, 2*N*_e_ was estimated to be 7.50 × 10^4^. All the parameters were then scaled by 2*N*_e_ to estimate time in years and the population size in number of individuals.

Domestication bottleneck was further verified using the BOTTLENECK program (version 1.2.02) which tests historical bottleneck by detecting the reductions of recent effective population size based on the comparison of heterozygosity [64]. A total of 1000 randomly selected SNPs were used in BOTTLENECK analysis. The tests were performed under the stepwise-mutation model (SMM), infinite allele model (IAM), and the two-phase model (TPM) allowing for 30% multistep changes. The iteration was set to 1000. We used the Wilcoxon signed rank tests to determine whether a population had a significant number of loci with excess genetic diversity.

### Genetic diversity and population differentiation

The nucleotide diversity (*π*) was calculated for each group with VCFtools [65] (version 0.1.12) using a 100-kb window and a step size of 10 kb. Genetic differentiation (*F*_ST_) was determined among different groups with VCFtools using a 100-kb window with a step size of 10 kb. Tajima’s *D* value was calculated with VariScan [66] (version 2.0.3) using a 100-kb window and a step size of 10 kb.

### Detection of selective sweeps

Genomic regions under selection often showed a decrease in genetic diversity. We identified genomic regions selected by domestication and improvement by comparing the ROD using a 100-kb window with a step size of 10 kb. For domestication, *π*_wild_/*π*_landrace_ was calculated, and the top 5% of windows or regions with highest ROD values were defined as domestication sweeps (*π*_wild_/*π*_landrace_ > 8.21). For improvement, *π*_landrace_/*π*_improved cultivars_ was calculated, and the top 5% of windows or regions with highest ROD values were defined as improvement sweeps (*π*_landrace_/*π*_improved cultivars_ > 4.28).

To confirm the selection sweeps identified by ROD, we also detected the selection signals using a likelihood-based method in the SweeD program (Version 3.1) [67]. The composite likelihood ratio (CLR) was calculated in domestication and improvement candidate regions using a grid of 2000. We found that more than 83% of the selective sweeps identified by ROD also showed selection signals by the CLR test (Additional file 3: Figure S21), indicating that most of the selection regions can be identified by both methods, which are thus reliable.

### Phenotyping

Seven qualitative traits, including flesh color (white/yellow), fruit hairiness (peach/nectarine), fruit shape (round/flat), fruit texture (melting/non-melting), flesh adhesion (adhesion/freestone), pollen fertility (male sterility/fertility), and fruit skin color (red/non-red skin) were measured based on previously published plant genetic resources evaluation criteria [68] in two successive years, from 2011 and 2012. The SSC was determined with a hand refractometer in 2007, 2010, and 2014 following the same protocol described in Frett et al. [69]. Ten peach fruits were sampled and homogenized to form a juice for each accession to determine the SSC. The fruit weight was determined as the average value of 10 fruits for each accession in 2007 and in 2010. The phenotypic value used for GWAS of these two quantitative traits was the mean of different measurements from different years. The frequency distribution of these two quantitative traits (SSC and fruit weight) is shown in Additional file 3: Figure S7. Total phenolics were extracted from 5 g fresh flesh combining five peach fruits using an extraction solution of 0.5 mol/L HCL in 800 mL/L methanol. The total phenolics were further evaluated with colorimetric methods and measured using a spectrophotometer (TU-1901, PERSEE, Beijing, China) as previously described [70]. Total phenolics was evaluated in 2015 and 2016, and the mean values used in GWAS.

For CR, two different kinds of phenotyping analyses were performed in 2011 and 2012. First, CR was considered a quantitative trait and determined following a protocol as previously described [38]*.* Average temperatures in 30-min intervals were continuously recorded using a temperature automatic recorder (LOGGER 95-4, http://www.hzjly.com) placed in the canopies of the experimental trees, starting in the middle of October. A 0–7.2 °C model was chosen to evaluate CR that was demonstrated to be suitable at Zhengzhou [38, 71]. The number of hours in 0–7.2 °C (chilling hours, CHs) was counted, starting when the daily average air temperature dropped to below 7.2 °C. Starting at 50 CHs, the branches of each accession were cut every 50 CHs until 1300 CHs. For each accession, two clones were sampled, and three branches longer than 40 cm with floral buds were taken from each clone. Branch cuttings were placed in water in a greenhouse at 25 °C and a 16 h/8 h photoperiod to force floral bud break. The frequency of floral bud break was evaluated after 2 weeks. The CR of an accession was defined as being sufficient at a specific sampling time if 50% of floral buds on the branch cuttings opened (Additional file 3: Figure S13). Secondly, CR was defined as a qualitative trait and evaluated using a similar method. Branches of each accession were cut only at 400 CHs. The frequency of floral bud break was evaluated after 2 weeks at forcing temperature. An accession was considered to have “low CR” if 50% of floral buds on the branch cuttings opened; otherwise, the accession was defined as a “non-low CR” accession. The results of the second measurement are shown in Additional file 1: Table S16.

### Genome-wide association studies

We used 374, 207, 415, 343, 343, 343, 165, 271, 361, 355, and 362 peach accessions to perform GWAS for flesh color, fruit hairiness, fruit shape, fruit texture, flesh adhesion, pollen fertility, total levels of phenolic compounds, skin color, fruit weight, fruit SSC, and CR, respectively. To improve the statistical power of the analysis, we filtered SNPs with minor allele frequency (MAF) < 0.05 or MAF < 0.01 after genotype imputation for each GWAS. A mixed linear model (MLM) program, Efficient Mixed-Model Association eXpedited (EMMAX) [72] (version beta), was used to carry out the GWAS analyses. To minimize false positives, population structure was taken into account using a kinship matrix that was estimated with the EMMAX emmax-kin program [72]. We defined the whole-genome significance cutoff as the Bonferroni test threshold, which was set as 0.05/total SNPs (−log_10_(*P*) = 7.19, −log_10_(*P*) = 7.62, −log_10_(*P*) = 7.19, −log_10_(*P*) = 7.62, −log_10_(*P*) = 7.62, −log_10_(*P*) = 7.62, −log_10_(*P*) = 7.18, −log_10_(*P*) = 7.12, −log_10_(*P*) = 7.49, −log_10_(*P*) = 7.59, −log_10_(*P*) = 7.38 for flesh color, fruit hairiness, fruit shape, fruit texture, flesh adhesion, pollen fertility, total levels of phenolic compounds, skin color, fruit weight, fruit SSC, and CR, respectively). Genome-wide SVs were also used to perform GWAS with the same accessions. The GWAS using SVs followed the same protocols of GWAS using SNPs.

### RNA-Seq analysis

Fruit samples were taken at six stages of fruit development from peach cultivar “Taijin Shui Mi”, including 20, 40, 60, 80, 100, and 120 days after bloom (DAB). Three biological replicates were collected for each stage. Fruit flesh was immediately frozen in liquid nitrogen and then ground to fine powder. Total RNA was extracted using a quick extraction kit (Aidlab, Beijing, China). First- and second-strand complementary DNA (cDNA) was synthesized using a cDNA Synthesis System kit (TOYOBO, Osaka, Japan), following the manufacturer’s protocol. Then double-strand cDNAs were purified and adapters were ligated to the short fragments. The constructed RNA-Seq libraries were sequenced using the Illumina HiSeq 2000 platform in paired-end 150-bp mode. Low-quality reads were filtered from the raw reads using Trimmomatic [73]. Data analysis followed the protocol proposed by Pertea et al. [74]. Cleaned reads were mapped to the peach reference genome using HISAT2 (Version 2.0.5) [75] using default parameters. Transcript abundances were estimated, and transcript assembly was performed using the Stringtie program [76]. DEG analysis was carried out using the R package ballgown [77].

### Overexpression of *SVP* gene in *Arabidopsis*

The full-length open reading frames of peach *SVP* gene (*Prupe.8G069300*) were amplified through PCR using cDNAs synthesized from RNA that were isolated from young leaves of a low CR landrace, “Nanshan Tian Tao” (CR = 200 h). The PCR products were further cloned into the pBI121 vector driven by the cauliflower mosaic virus (CaMV) 35S promoter at Sangon Biotech (Sangon, Shanghai, China). The resulting constructs were further transformed into *Arabidopsis thaliana* Columbia type by *Agrobacterium tumefaciens* GV3101 and selected with kanamycin. The rosetteleaf numbers of 10 transgenic lines were used to evaluate the flowering time. The primers used for gene cloning are listed in Additional file 2: Table S23.

### Expression analysis using qRT-PCR

Total RNA were extracted from the fruit flesh of 10 accessions at maturity, including “Kashi 2,” “Kashi 3,” “Kashi 4,” “Xinjiang Huang Rou,” “Zao Shu Huang Gan,” “Lin Huang 1,” “Reddomun,” “Gua Tao,” “Shaji 2,” “Hang Zhou Zao Shui Mi,” using an extraction kit (Aidlab, Beijing, China). First-strand cDNA synthesis was carried out using 1 mg RNA and the Transcriptor First Strand cDNA synthesis kit (Takara, Dalian, China), according to the manufacturer’s protocol. Sequence of candidate gene for SSC, *Prupe.4G150100*, was downloaded from the Genome Database for Rosaceae (GDR; www.rosaceae.org). Gene-specific primers were designed using the Primer-BLAST software (National Center for Biotechnology Information, Maryland, USA). We performed qRT-PCR using the LightCycler System (Roche LightCycler 480; Roche Diagnostics), following the manufacturer’s protocol. Relative expression levels were estimated by the 2^−ΔΔCT^ method. The primers were listed in Additional file 2: Table S23.

## Additional files

Additional file 1: **Table S1.** Sequencing summary of 480 peach accessions. **Table S8.** Putative domestication sweeps. **Table S9.** Putative improvement sweeps. **Table S10.** Genes in putative domestication sweeps. **Table S11.** Genes in putative improvement sweeps. **Table S15.** Putative selective sweeps for low CR breeding. **Table S16.** Chilling requirement test by classifying accessions into low (1) and non-low CR (0). **Table S17.** Putative improvement sweeps in western improved group. **Table S18.** Putative improvement sweeps in eastern improved group. (XLSX 576 kb)

Additional file 2: **Table S2.** Distribution and summary of genome-wide variants. **Table S3.** Summary of SNP annotations. **Table S4.** SNP loci (30) selected for validation by Sequenom MassARRAY. **Table S5.** GWAS results for six traits using SNPs and SVs. **Table S6.** Estimation of domestication bottlenecks in peach and other crop and fruit species. **Table S7.** Domestication bottleneck verified using BOTTLENECK. **Table S12.** Genomic regions continuously selected by both domestication and improvement. **Table S13.** Summary of SNPs associated with SSC and fruit weight. **Table S14.** SNPs associated with chilling requirement. **Table S19.** Shared selective sweeps between western and eastern improved groups. **Table S20.** Eastern specific improvement sweeps. **Table S21.** Western group specific improvement sweeps. **Table S22.** Best fitting parameters for the two-population model for cultivated and wild peach groups in the demographic analysis. **Table S23.** Primers used in this study. (DOC 486 kb)

Additional file 3: **Figure S1.** Genetic diversity of the 480 peach accessions. **Figure S2.** Manhattan plot and QQ plot of genome-wide association studies on seven important agronomic traits. **Figure S3.** A candidate 70.5-kb deletion underlying fruit texture. **Figure S4.** Phenetic neighbor-joining tree of 480 peach accessions constructed using PHYLIP with 100 bootstrap replicates. **Figure S5.** Population structure of the 480 peach accessions. **Figure S6.** Phenotypes and nucleotide diversity changes during peach improvement and domestication. **Figure S7.** Genome-wide association studies (GWAS) of SSC and fruit weight. **Figure S8.** Regional LD decay measured by *R*^2^ at the 20.0–30.0-Mb region of chromosome 2. **Figure S9.** Total organic acids in domesticated peaches and wild relatives. **Figure S10.** Genome-wide association study of total phenolic content for peach. **Figure S11.** Expression profile of a sugar transport gene, *Prupe.4G037800*, during fruit development in peach. **Figure S12.** A candidate gene for SSC associated with increase of fruit taste during domestication. **Figure S13.** Distribution of chilling requirement in 371 peach accessions. **Figure S14.** Manhattan plot (left) and QQ plot (right) of GWAS for chilling requirement (CR) by classifying accessions into low CR and non-low CR. **Figure S15.** Comparison of flowering times between *Arabidopsis* overexpressing of peach *SVP* gene (OE) and the wild type (WT) *Arabidopsis*. **Figure S16.** A PCR-based marker for low CR peach identification based on the results of CR GWAS. **Figure S17.** Comparison of improvement sweeps between eastern and western improved groups. **Figure S18.** Shared and private SVs among three callers. **Figure S19.** Distribution of genotype missing rate for 480 peach accessions. **Figure S20.** Demographic model for peach domestication and spread. **Figure S21.** Shared domestication and improvement sweeps identified by ROD and XP-CLR methods. (PDF 2514 kb)

Additional file 4: Review history. (DOCX 197 kb)

## References

[1] Borlaug NE (1983). Contributions of conventional plant breeding to food production. Science.

[2] Huang X, Wei X, Sang T, Zhao Q, Feng Q, Zhao Y (2010). Genome-wide association studies of 14 agronomic traits in rice landraces. Nat Genet.

[3] Jiao Y, Zhao H, Ren L, Song W, Zeng B, Guo J (2012). Genome-wide genetic changes during modern breeding of maize. Nat Genet.

[4] Qi J, Liu X, Shen D, Miao H, Xie B, Li X (2013). A genomic variation map provides insights into the genetic basis of cucumber domestication and diversity. Nat Genet.

[5] Lin T, Zhu G, Zhang J, Xu X, Yu Q, Zheng Z (2014). Genomic analyses provide insights into the history of tomato breeding. Nat Genet.

[6] Zhou Z, Jiang Y, Wang Z, Gou Z, Lyu J, Li W (2015). Resequencing 302 wild and cultivated accessions identifies genes related to domestication and improvement in soybean. Nat Biotechnol.

[7] Cao K, Zhou Z, Wang Q, Guo J, Zhao P, Zhu G (2016). Genome-wide association study of 12 agronomic traits in peach. Nat Commun.

[8] Duan N, Bai Y, Sun H, Wang N, Ma Y, Li M (2017). Genome re-sequencing reveals the history of apple and supports a two-stage model for fruit enlargement. Nat Commun.

[9] Dirlewanger E, Graziano E, Joobeur T, Garriga-Calderé F, Cosson P, Howad W (2004). Comparative mapping and marker-assisted selection in Rosaceae fruit crops. Proc Natl Acad Sci U S A.

[10] Cirilli M, Bassi D, Ciacciulli A (2016). Sugars in peach fruit: a breeding perspective. Hortic Res.

[11] Cao K, Zheng Z, Wang L, Liu X, Zhu G, Fang W (2014). Comparative population genomics reveals the domestication history of the peach, *Prunus persica*, and human influences on perennial fruit crops. Genome Biol.

[12] Verde I, Abbott AG, Scalabrin S, Jung S, Shu S, Marroni F (2013). The high-quality draft genome of peach (*Prunus persica*) identifies unique patterns of genetic diversity, domestication and genome evolution. Nat Genet.

[13] Falchi R, Vendramin E, Zanon L, Scalabrin S, Cipriani G, Verde I (2013). Three distinct mutational mechanisms acting on a single gene underpin the origin of yellow flesh in peach. Plant J.

[14] Dirlewanger E, Cosson P, Boudehri K, Renaud C, Capdeville G, Tauzin Y (2006). Development of a second-generation genetic linkage map for peach [*Prunus persica* (L.) Batsch] and characterization of morphological traits affecting flower and fruit. Tree Genet Genomes..

[15] Vendramin E, Pea G, Dondini L, Pacheco I, Dettori MT, Gazza L (2014). A unique mutation in a *MYB* gene cosegregates with the nectarine phenotype in peach. PLoS One.

[16] Gu C, Wang L, Wang W, Zhou H, Ma B, Zheng H (2016). Copy number variation of a gene cluster encoding endopolygalacturonase mediates flesh texture and stone adhesion in peach. J Exp Bot.

[17] Yamamoto T, Shimada T, Imai T, Yaegaki H, Haji T, Matsuta N (2001). Characterization of morphological traits based on a genetic linkage map in peach. Breeding Sci.

[18] Velasco D, Hough J, Aradhya M, Ross-Ibarra J (2016). Evolutionary genomics of peach and almond domestication. G3-Genes Genom Genet.

[19] Zhou Y, Massonnet M, Sanjak JS, Cantu D, Guat B (2017). Evolutionary genomics of grape (*Vitis viniferassp*. Vinifera) domestication. Proc Natl Acad Sci U S A.

[20] Cornuet JM, Luikart G (1996). Description and power analysis of two tests for detecting recent population bottlenecks from allele frequency data. Genetics.

[21] Gutenkunst RN, Hernandez RD, Williamson SH, Bustamante CD (2009). Inferring the joint demographic history of multiple populations from multidimensional SNP frequency data. PLoS Genet.

[22] Ross-Ibarra J, Tenaillon M, Gaut BS (2009). Historical divergence and gene flow in the genus *Zea*. Genetics.

[23] Caicedo AL, Williamson SH, Hernandez RD, Boyko A, Fledel-Alon A, York TL (2007). Genome-wide patterns of nucleotide polymorphism in domesticated rice. PLoS Genet.

[24] Arroyo-García R, Ruizgarcía L, Bolling L, Ocete R, López MA, Arnold C (2006). Multiple origins of cultivated grapevine (*Vitis vinifera* L. ssp. sativa) based on chloroplast DNA polymorphisms. Mol Ecol.

[25] Dirlewanger E, Moing A, Rothan C, Svanella L, Pronier V, Guye A (1999). Mapping QTLs controlling fruit quality in peach (*Prunus persica* (L.) Batsch). Theor Appl Genet.

[26] Quilot B, Wu B, Kervella J, Génard M, Foulongne M, Moreau K (2004). QTL analysis of quality traits in an advanced backcross between *Prunus persica*, cultivars and the wild relative species *P. davidiana*. Theor Appl Genet.

[27] Martínez-García PJ, Parfitt DE, Ogundiwin EA, Fass J, Chan HM, Ahmad R (2013). High density SNP mapping and QTL analysis for fruit quality characteristics in peach (*Prunus persica* L.). Tree Genet & Genomes..

[28] Zeballos JL, Abidi W, Giménez R, Monforte AJ, Moreno MA, Gogorcena Y (2016). Mapping QTLs associated with fruit quality traits in peach [*Prunus persica* (L.) Batsch] using SNP maps. Tree Genet & Genomes.

[29] Frary A, Nesbitt TC, Grandillo S, Knaap E, Cong B, Liu J (2000). *fw2.2*: a quantitative trait locus key to the evolution of tomato fruit size. Science.

[30] Wang LR, Zhu GR, Fang WC (2012). Peach genetics resources in China.

[31] Etienne C, Rothan C, Moing A, Plomion C, Bodénès C, Svanella-Dumas L (2002). Candidate genes and QTLs for sugar and organic acid content in peach [*Prunus persica* (L.) Batsch]. Theor Appl Genet.

[32] Eduardo I, Pacheco I, Chietera G, Bassi D, Pozzi C, Vecchietti A (2011). QTL analysis of fruit quality traits in two peach intraspecific populations and importance of maturity date pleiotropic effect. Tree Genet Genomes..

[33] Predieri S, Ragazzini P, Rondelli R (2006). Sensory evaluation and peach fruit quality. Acta Hort.

[34] Jia HF, Chai YM, Li CL, Lu D, Luo JJ, Qin L (2011). Abscisic acid plays an important role in the regulation of strawberry fruit ripening. Plant Physiol.

[35] Wang M, Tu L, Lin M, Lin Z, Wang P, Yang Q, Ye Z (2017). Asymmetric subgenome selection and *cis*-regulatory divergence during cotton domestication. Nat Genet.

[36] Li Y, Wang L, Zhu G, Fang W, Cao K, Chen C (2016). Phenological response of peach to climate change exhibits a relatively dramatic trend in China, 1983-2012. Sci Hortic-Amsterdam.

[37] Sansavini S, Gamberini A, Bassi D (2006). Peach breeding, genetics and new cultivar trends. Acta Hort.

[38] Fan S, Bielenberg DG, Zhebentyayeva TN, Reighard GL, Okie WR, Holland D (2010). Mapping quantitative trait loci associated with chilling requirement, heat requirement and bloom date in peach (*Prunus persica*). New Phytol.

[39] Bielenberg DG, Rauh B, Fan S, Gasic K, Abbott AG, Reighard GL (2015). Genotyping by sequencing for SNP-based linkage map construction and QTL analysis of chilling requirement and bloom date in peach [*Prunus persica* (L.) Batsch]. PLoS One.

[40] Bielenberg DG, Wang Y, Li Z, Zhebentyayeva T, Fan S, Reighard GL (2008). Sequencing and annotation of the evergrowing locus in peach [*Prunus persica* (L.) Batsch] reveals a cluster of six MADS-box transcription factors as candidate genes for regulation of terminal bud formation. Tree Genet Genomes..

[41] Bielenberg DG, Wang Y, Fan S, Reighard GL, Scorza R, Abbott AG (2004). A deletion affecting several gene candidates is present in the *evergrowing* peach mutant. J Hered..

[42] Gradziel TM, Mccaa JP. Processing peach cultivar development. In: Layne D, Bassi D, editors. The peach: botany, production and uses. Oxfordshire: CABI; 2008. p. 175–92.

[43] Tieman D, Zhu G, Resende MF, Lin T, Nguyen C, Bies D (2017). A chemical genetic roadmap to improved tomato flavor. Science.

[44] Tester M, Langridge P (2010). Breeding technologies to increase crop production in a changing world. Science.

[45] Murray MG, Thompson WF (1980). Rapid isolation of high molecular weight plant DNA. Nucleic Acids Res.

[46] Li H, Durbin R (2009). Fast and accurate short read alignment with Burrows-Wheeler transform. Bioinformatics.

[47] McKenna A, Hanna M, Banks E, Sivachenko A, Cibulskis K, Kernytsky A (2010). The genome analysis toolkit: a MapReduce framework for analyzing next-generation DNA sequencing data. Genome Res.

[48] Quinlan AR, Hall IM (2010). BEDTools: a flexible suite of utilities for comparing genomic features. Bioinformatics.

[49] Li H, Handsaker B, Wysoker A, Fennell T, Ruan J, Homer N (2009). The sequence alignment/map format and SAMtools. Bioinformatics.

[50] DePristo MA, Banks E, Poplin R, Garimella KV, Maguire JR, Hartl C (2011). A framework for variation discovery and genotyping using next-generation DNA sequencing data. Nat Genet.

[51] Chiang C, Layer RM, Faust GG, Lindberg MR, Rose DB, Garrison EP (2015). SpeedSeq: ultra-fast personal genome analysis and interpretation. Nat Methods.

[52] Tobias R, Thomas Z, Andreas S, Adrian MS, Vladimir B, Jan OK (2012). Delly: structural variant discovery by integrated paired-end and split-read analysis. Bioinformatics.

[53] Chen X, Schulz-Trieglaff O, Shaw R, Barnes B, Schlesinger F, Källberg M (2016). Manta: rapid detection of structural variants and indels for germline and cancer sequencing applications. Bioinformatics.

[54] Jeffares DC, Jolly C, Hoti M, Speed D, Shaw L, Rallis C (2017). Transient structural variations have strong effects on quantitative traits and reproductive isolation in fission yeast. Nat Commun.

[55] Cingolani P, Platts A, Wang Le L, Coon M, Nguyen T, Wang L (2012). A program for annotating and predicting the effects of single nucleotide polymorphisms, SnpEff. Fly.

[56] Browning BL, Browning SR (2016). Genotype imputation with millions of reference samples. Am J Hum Genet.

[57] Felsenstein J (1989). PHYLIP-phylogeny inference package (version 3.2). Cladistics.

[58] He Z, Zhang H, Gao S, Lercher MJ, Chen WH, Hu S (2016). Evolview v2: an online visualization and management tool for customized and annotated phylogenetic trees. Nucleic Acids Res.

[59] Yang J, Lee SH, Goddard ME, Visscher PM (2011). GCTA: a tool for genome-wide complex trait analysis. Am J Hum Genet.

[60] Falush D, Stephens M, Pritchard JK (2013). Inference of population structure using multilocus genotype data: linked loci and correlated allele frequencies. Genetics.

[61] Earl DA, Vonholdt BM (2012). STRUCTURE HARVESTER: a website and program for visualizing STRUCTURE output and implementing the Evanno method. Conserv Genet Resour.

[62] Purcell S, Neale B, Todd-Brown K, Thomas L, Ferreira MA, Bender D (2007). PLINK: a tool set for whole-genome association and population-based linkage analyses. Am J Hum Genet.

[63] Xie Z, Wang L, Wang L, Wang Z, Lu Z, Tian D (2016). Mutation rate analysis via parent-progeny sequencing of the perennial peach I A low rate in woody perennials and a higher mutagenicity in hybrids. P Roy Soc B-Biol Sci.

[64] Piry S, Luikart G, Cornuet JM (1999). BOTTLENECK: a computer program for detecting recent reductions in the effective population size using allele frequency data. J Hered.

[65] Danecek P, Auton A, Abecasis G, Albers CA, Banks E, DePristo MA (2011). The variant call format and VCFtools. Bioinformatics.

[66] Vilella AJ, Blanco-Garcia A, Hutter S, Rozas J (2005). VariScan: analysis of evolutionary patterns from large-scale DNA sequence polymorphism data. Bioinformatics.

[67] Pavlidis P, Živković D, Stamatakis A, Alachiotis N (2013). SweeD: likelihood-based detection of selective sweeps in thousands of genomes. Mol Biol & Evol.

[68] Wang L, Zhu G (2005). Descripters and data standard for peach.

[69] Frett TJ, Gasic K, Clark JR, Byrne D, Gradziel T, Crisosto C (2012). Standardized phenotyping for fruit quality in peach [*Prunus persica* (L.) Batsch]. J Am Pomol Soc.

[70] Abidi W, Cantín CM, Jiménez S, Giménez R, Moreno MA, Gogorcena Y (2015). Effect of antioxidant compounds and total sugars and genetic background on the chilling injury susceptibility of a non-melting peach [*Prunus persica* (L.) Batsch] progeny. J Sci Food Agric.

[71] Wang LR, Zhu GR, Fang WC, Zuo QY (2003). Estimating models of the chilling requirement for peach. Acta Hortic Sin.

[72] Kang HM, Sul JH, Zaitlen NA, Kong SY, Freimer NB, Service SK (2010). Variance component model to account for sample structure in genome-wide association studies. Nat Genet.

[73] Bolger AM, Lohse M, Usadel B (2014). Trimmomatic: a flexible trimmer for Illumina sequence data. Bioinformatics.

[74] Pertea M, Kim D, Pertea G, Leek JT, Salzberg SL (2016). Transcript-level expression analysis of RNA-seq experiments with HISAT, StringTie and Ballgown. Nat Protoc.

[75] Kim D, Langmead B, Salzberg SL (2015). HISAT: a fast spliced aligner with low memory requirements. Nat Methods.

[76] Pertea M, Pertea GM, Antonescu CM, Chang TC, Mendell JT, Salzberg SL (2015). StringTie enables improved reconstruction of a transcriptome from RNA-seq reads. Nat Biotechnol.

[77] Frazee AC, Pertea G, Jaffe AE, Langmead B, Salzberg SL, Leek JT (2015). Ballgown bridges the gap between transcriptome assembly and expression analysis. Nat Biotechnol.

[78] Li Y, Cao K, Zhu G, Fang W, Chen C, Wang X, et al. Genomic analyses of an extensive collection of wild and cultivated accessions provide new insights into peach breeding history. Sequence Read Archive: PRJNA388029. https://www.ncbi.nlm.nih.gov/bioproject/PRJNA388029. Accessed 2017. 10.1186/s13059-019-1648-9 PMC6383288 30791928

[79] Li Y, Cao K, Zhu G, Fang W, Chen C, Wang X, et al. Genomic analyses of an extensive collection of wild and cultivated accessions provide new insights into peach breeding history. Figshare. 10.6084/m9.figshare.5008988.v4. Accessed 2017. 10.1186/s13059-019-1648-9 PMC6383288 30791928

[80] Li Y, Cao K, Zhu G, Fang W, Chen C, Wang X, et al. Genomic analyses of an extensive collection of wild and cultivated accessions provide new insights into peach breeding history. Figshare. https://figshare.com/articles/Peach_SVs/7238186. Accessed 2018. 10.1186/s13059-019-1648-9 PMC6383288 30791928

