# Supplementary material for: Genomic analyses of an extensive collection of wild and cultivated accessions provide new insights into peach breeding history
Source: Genome Biol. 2019 Feb 21;20:36. doi: 10.1186/s13059-019-1648-9 (PMC6383288; doi:10.1186/s13059-019-1648-9)
Supplement: Supplementary file 2 — Table S2. Distribution and summary of genome-wide variants. Table S3. Summary of SNP annotations. Table S4. SNP loci (30) selected for validation by Sequenom MassARRAY. Table S5. GWAS results for six traits using SNPs and SVs. Table S6. Estimation of domestication bottlenecks in peach and other crop and fruit species. Table S7. Domestication bottleneck verified using BOTTLENECK. Table S12. Genomic regions continuously selected by both domestication and improvement. Table S13. Summary of SNPs associated with SSC and fruit weight. Table S14. SNPs associated with chilling requirement. Table S19. Shared selective sweeps between western and eastern improved groups. Table S20. Eastern specific improvement sweeps. Table S21. Western group specific improvement sweeps. Table S22. Best fitting parameters for the two-population model for cultivated and wild peach groups in the demographic analysis. Table S23. Primers used in this study. (DOC 486 kb) [file 13059_2019_1648_MOESM2_ESM.doc]

**Table S2 Distribution and summary of genome-wide variants.**

| **Chr.** | **Length** | **No. SNPs** | **No. INDELs^b^** | **No. SVs** | **No. SNPs per kb** | **Total No. Variants^c^** | **No. variants per kb^d^** |
| --- | --- | --- | --- | --- | --- | --- | --- |
| Pp01 | 47,851,208 | 1,044,277 | 209,982 | 24,997 | 21.8 | 1,279,256 | 26.7 |
| Pp02 | 30,405,870 | 667,612 | 145,731 | 22,963 | 22.0 | 836,306 | 27.5 |
| Pp03 | 27,368,013 | 600,395 | 121,469 | 18,521 | 21.9 | 740,385 | 27.1 |
| Pp04 | 25,843,236 | 557,764 | 120,131 | 19,536 | 21.6 | 697,431 | 27.0 |
| Pp05 | 18,496,696 | 403,530 | 80,760 | 14,198 | 21.8 | 498,488 | 27.0 |
| Pp06 | 30,767,194 | 680,428 | 138,992 | 21,382 | 22.1 | 840,802 | 27.3 |
| Pp07 | 22,388,614 | 507,680 | 102,480 | 17,839 | 22.7 | 627,999 | 28.0 |
| Pp08 | 22,573,980 | 500,858 | 102,750 | 19,894 | 22.2 | 623,502 | 27.6 |
| Scaffolds^a^ | 1,668,921 | 17,715 | 4,080 | N.A. | N.A. | 21,795 | N.A. |
| Total | 227,363,732 | 4,980,259 | 1,026,375 | 159,330 | 21.9 | 6,165,964 | 27.3 |

^a^SNote: 00000000000000000000000000000000000000000000000000000000000000000000000000000000000000000000000000000000000000000000000000SsSSSSSS

caffolds indicate the sequences that could not be anchored on the eight peach chromosomes.

^b^INDELs indicate the insertions and deletions shorter than 5 bp.

^c^Total variants include SNPs, small INDELs, and SVs.

^d^Variants per kb was calculated based on SNPs, INDELs, and SVs.

**Table S3** Summary of SNP annotations. Note: Gene models were based on genome annotation version 2.1.

|  | | |
| --- | --- | --- |
| **Type (alphabetical order)** | **Count** | **Percent** |
| 3_prime_UTR_variant | 421,249 | 2.39% |
| 5_prime_UTR_premature_start_codon_gain_variant | 40,684 | 0.23% |
| 5_prime_UTR_variant | 268,550 | 1.53% |
| downstream_gene_variant | 5,265,096 | 29.89% |
| initiator_codon_variant | 119 | 0.00% |
| initiator_codon_variant+non_canonical_start_codon | 1 | 0% |
| intergenic_region | 3,127,764 | 17.76% |
| intron_variant | 2,002,832 | 11.37% |
| missense_variant | 448,602 | 2.55% |
| missense_variant+splice_region_variant | 7,552 | 0.04% |
| missense_variant+splice_region_variant+splice_region_variant | 1 | 0% |
| splice_acceptor_variant+intron_variant | 2,086 | 0.01% |
| splice_acceptor_variant+splice_donor_variant+intron_variant | 1 | 0% |
| splice_acceptor_variant+splice_region_variant+intron_variant | 7 | 0% |
| splice_donor_variant+intron_variant | 1,724 | 0.01% |
| splice_donor_variant+splice_region_variant+intron_variant | 4 | 0% |
| splice_region_variant | 5,721 | 0.03% |
| splice_region_variant+intron_variant | 57,825 | 0.33% |
| splice_region_variant+splice_region_variant+intron_variant | 12 | 0% |
| splice_region_variant+stop_retained_variant | 105 | 0.00% |
| splice_region_variant+synonymous_variant | 7,437 | 0.04% |
| start_lost | 800 | 0.01% |
| start_lost+splice_region_variant | 7 | 0% |
| stop_gained | 6,158 | 0.04% |
| stop_gained+splice_region_variant | 109 | 0.00% |
| stop_lost | 673 | 0.00% |
| stop_lost+splice_region_variant | 234 | 0.00% |
| stop_retained_variant | 582 | 0.00% |
| synonymous_variant | 422,122 | 2.40% |
| upstream_gene_variant | 5,525,700 | 31.37% |

**Table S4** SNP loci (30) selected for validation by Sequenom MassARRAY.

| Chr. | Position | Missing data | Consistent | Consistent after imputation | Inconsistent | Inconsistent after imputation | Accuracy | Accuracy after imputation |
| --- | --- | --- | --- | --- | --- | --- | --- | --- |
| Pp01 | 13090027 | 17 | 225 | 242 | 16 | 16 | 93.36% | 93.80% |
| Pp01 | 25801464 | 8 | 234 | 242 | 16 | 16 | 93.60% | 93.80% |
| Pp01 | 33292876 | 13 | 229 | 242 | 16 | 16 | 93.47% | 93.80% |
| Pp01 | 36645183 | 23 | 235 | 258 | 0 | 0 | 100.00% | 100.00% |
| Pp02 | 927910 | 7 | 235 | 242 | 16 | 16 | 93.63% | 93.80% |
| Pp02 | 17911562 | 17 | 236 | 253 | 5 | 5 | 97.93% | 98.06% |
| Pp02 | 21512032 | 9 | 241 | 249 | 8 | 9 | 96.79% | 96.51% |
| Pp02 | 24627681 | 4 | 254 | 258 | 0 | 0 | 100.00% | 100.00% |
| Pp02 | 25055364 | 76 | 175 | 251 | 7 | 7 | 96.15% | 97.29% |
| Pp02 | 25257979 | 11 | 240 | 251 | 7 | 7 | 97.17% | 97.29% |
| Pp04 | 2040124 | 7 | 236 | 243 | 15 | 15 | 94.02% | 94.19% |
| Pp04 | 8368298 | 52 | 206 | 258 | 0 | 0 | 100.00% | 100.00% |
| Pp04 | 10096595 | 7 | 250 | 257 | 1 | 1 | 99.60% | 99.61% |
| Pp04 | 14047723 | 6 | 251 | 257 | 1 | 1 | 99.60% | 99.61% |
| Pp04 | 19072499 | 129 | 124 | 253 | 5 | 5 | 96.12% | 98.06% |
| Pp05 | 541076 | 11 | 232 | 240 | 15 | 18 | 93.93% | 93.02% |
| Pp05 | 693216 | 19 | 223 | 242 | 16 | 16 | 93.31% | 93.80% |
| Pp05 | 1503088 | 19 | 226 | 245 | 13 | 13 | 94.56% | 94.96% |
| Pp05 | 17571575 | 3 | 250 | 253 | 5 | 5 | 98.04% | 98.06% |
| Pp05 | 18232588 | 8 | 240 | 248 | 10 | 10 | 96.00% | 96.12% |
| Pp06 | 3024748 | 17 | 232 | 245 | 9 | 13 | 96.27% | 94.96% |
| Pp06 | 7232688 | 9 | 238 | 247 | 11 | 11 | 95.58% | 95.74% |
| Pp06 | 24128826 | 9 | 231 | 240 | 18 | 18 | 92.77% | 93.02% |
| Pp06 | 28973642 | 6 | 237 | 243 | 15 | 15 | 94.05% | 94.19% |
| Pp06 | 29262453 | 10 | 239 | 247 | 9 | 11 | 96.37% | 95.74% |
| Pp06 | 29888797 | 9 | 247 | 256 | 2 | 2 | 99.20% | 99.22% |
| Pp08 | 14171211 | 19 | 233 | 252 | 6 | 6 | 97.49% | 97.67% |
| Pp08 | 15920832 | 19 | 229 | 248 | 10 | 10 | 95.82% | 96.12% |
| Pp08 | 17472711 | 11 | 231 | 241 | 16 | 17 | 93.52% | 93.41% |
| Pp08 | 19873864 | 66 | 181 | 247 | 11 | 11 | 94.27% | 95.74% |

|  |  | |  | | | | | | |  |
| --- | --- | --- | --- | --- | --- | --- | --- | --- | --- | --- |
| **Trait** | | **Marker Type** | | **Chr.** | **Pos.** | **SV**  **type** | **SV**  **length** | ***P* value** | **Known QTL or genes** | |
| Flesh color | | SNP | | Pp01 | 27,005,584 | NA | NA | 8.40e-11 | *PpCCD4,* ref. 13 | |
| Fruit hairiness | | SNP | | Pp05 | 16,633,286 | NA | NA | 4.26e-28 | *PpMYB25,* ref. 15 | |
| Fruit shape | | SNP | | Pp06 | 26,288,291 | NA | NA | 4.65e-22 | *S,* ref. 14 | |
| Fruit texture | | SNP | | Pp04 | 19,909,362 | NA | NA | 6.53e-13 | *FT,* ref. 16 | |
| Flesh adhesion | | SNP | | Pp04 | 19,070,801 | NA | NA | 8.31e-08 | *FT,* ref. 16 | |
| Pollen fertility | | SNP | | Pp06 | 2,272,667 | NA | NA | 5.11e-17 | *Ps,* ref. 14 | |
| Fruit skin color | | SNP | | Pp03 | 18,103,021 | NA | NA | 8.34e-10 | *Skc, MYB10.1,* ref. 17 | |
| Flesh color | | SV | | Pp01 | 27,024,088 | deletion | 317 | 3.10e-10 | *PpCCD4,* ref. 13 | |
| Fruit hairiness | | SV | | Pp05 | 16,715,836 | deletion | 421 | 1.05e-35 | *PpMYB25,* ref. 15 | |
| Fruit shape | | SV | | Pp06 | 26,847,156 | inversion | 1,669,466 | 4.11e-52 | *S,* ref. 14 | |
| Fruit texture | | SV | | Pp04 | 19,026,186 | deletion | 70,494 | 1.39e-11 | *FT,* ref. 16 | |
| Flesh adhesion | | SV | | Pp04 | 19,026,186 | deletion | 70,494 | 7.72e-06 | *FT,* ref. 16 | |
| Pollen fertility | | SV | | Pp06 | 2,014,933 | deletion | 3,362 | 3.46e-09 | *Ps,* ref. 14 | |
| Fruit skin color | | SV | | Pp03 | 18,458,506 | deletion | 6,420 | 1.53e-10 | *Skc, MYB10.1,* ref.17 | |

**Table S5** GWAS results for six traits using SNPs and SVs. Only top signals of each GWAS were listed.

**Table S6** Estimation of domestication bottlenecks in peach and other crop and fruit species.

| Species | Population | No. accessions | Diversity | Bottleneck (π_wild_/π_landrace_) | Reference |
| --- | --- | --- | --- | --- | --- |
| Peach | Wild | 52 | 0.0035 | 2.92 | This study |
|  | Landrace | 215 | 0.0012 |  |  |
| Maize | Wild | 17 | 0.0059 | 1.20 | ref. 22 |
|  | Landrace | 23 | 0.0048 |  |  |
| Rice | Wild | 446 | 0.003 | 1.25 | ref. 23 |
|  | Landrace | 1083 | 0.0024 |  |  |
| Soybean | Wild | 62 | 0.0029 | 2.07 | ref. 6 |
|  | Landrace | 130 | 0.0014 |  |  |
| Apple | Wild | 15 | 0.00235 | 1.07 | ref. 8 |
|  | Landrace | 35 | 0.0022 |  |  |
| Grape | Wild | 9 | 0.0147 | 1.06 | ref. 19 |
|  | Landrace | 14 | 0.0139 |  |  |

**Table S7** Domestication bottleneck verified using BOTTLENECK. I.A.M, infinite allele model; T.P.M., the two-phase model; S.M.M., the stepwise-mutation model.

| Assumptions | I.A.M. | T.P.M. | S.M.M. |
| --- | --- | --- | --- |
| Probability (one tail)  Probability (two tails) | 0.00003  0.00005 | 0.00016  0.00032 | 0.00051  0.00012 |

| **Table S12** Genomic regions continuously selected by both domestication and improvement. | | | |
| --- | --- | --- | --- |
| **Chromosome** | **Start (bp)** | **End (bp)** | **Known QTL or gene** |
| Pp01 | 5760001 | 5790000 |  |
| Pp01 | 8510001 | 8610000 |  |
| Pp01 | 9550001 | 9650000 | *fru*, ref. 28 |
| Pp01 | 26190001 | 26290000 | *SSC*, *cit,* ref. 28 and ref. 32 |
| Pp01 | 39240001 | 39380000 |  |
| Pp01 | 44440001 | 44540000 |  |
| Pp02 | 24900001 | 24980000 |  |
| Pp02 | 27140001 | 27270000 |  |
| Pp02 | 27400001 | 27710000 |  |
| Pp03 | 18260001 | 18350000 | *skc,* ref. 17 |
| Pp03 | 21810001 | 21930000 |  |
| Pp03 | 24330001 | 24410000 |  |
| Pp03 | 24450001 | 24550000 |  |
| Pp04 | 7590001 | 7670000 |  |
| Pp04 | 7700001 | 7850000 |  |
| Pp05 | 7880001 | 7950000 | *sor*, *SSC*, ref. 28 and ref. 25 |
| Pp05 | 12210001 | 12330000 |  |
| Pp05 | 12680001 | 12700000 | *glu, fru*, ref, 28 |
| Pp05 | 15230001 | 15350000 | *fw,* ref. 28 |
| Pp05 | 15480001 | 15620000 |  |
| Pp05 | 15650001 | 15670000 |  |
| Pp05 | 16040001 | 16190000 |  |
| Pp05 | 16350001 | 16480000 |  |
| Pp05 | 17500001 | 17510000 |  |
| Pp05 | 17640001 | 17690000 |  |
| Pp05 | 17880001 | 17910000 |  |
| Pp05 | 17720001 | 17860000 |  |
| Pp05 | 17940001 | 18110000 |  |
| Pp05 | 18240001 | 18380000 | *fw,* ref. 26 and ref. 28 |
| Pp06 | 4930001 | 4970000 | *SSC,* ref. 28 |
| Pp06 | 30110001 | 30260000 | *SSC,* ref. 28 |
| Pp06 | 30400001 | 30740000 |  |
| Pp07 | 20260001 | 20420000 |  |
| Pp07 | 21570001 | 21730000 |  |
| Pp08 | 22170001 | 22260000 | *Prupe.8G264300,* sucrose synthase |
| Pp08 | 22370001 | 22520000 |  |

**Table S13** Summary of SNPs associated with SSC and fruit weight.

| Trait | Chr. | Positions | Major allele | Major allele frequency | Minor allele | Minor allele frequency | P value | known loci |
| --- | --- | --- | --- | --- | --- | --- | --- | --- |
| Fruit weight | Pp01 | 8241258 | G | 0.991398 | A | 0.00860215 | 3.18E-09 |  |
| Fruit weight | Pp01 | 13805853 | T | 0.917031 | C | 0.0829694 | 5.74E-09 |  |
| Fruit weight | Pp01 | 21723330 | G | 0.991587 | A | 0.00841346 | 5.74E-09 |  |
| Fruit weight | Pp01 | 27399500 | G | 0.911184 | A | 0.0888158 | 4.77E-09 | *fw*, ref. 28 |
| Fruit weight | Pp01 | 35862721 | A | 0.946835 | G | 0.0531646 | 5.74E-09 |  |
| Fruit weight | Pp01 | 35985907 | A | 0.919248 | G | 0.0807522 | 4.57E-09 |  |
| Fruit weight | Pp02 | 3355248 | T | 0.975824 | C | 0.0241758 | 1.90E-09 |  |
| Fruit weight | Pp02 | 20659027 | G | 0.951482 | C | 0.0485175 | 5.74E-09 |  |
| Fruit weight | Pp02 | 21689536 | T | 0.989899 | C | 0.010101 | 1.22E-08 |  |
| Fruit weight | Pp02 | 27740171 | G | 0.90583 | A | 0.0941704 | 2.95E-10 |  |
| Fruit weight | Pp02 | 27740499 | C | 0.90393 | T | 0.0960699 | 3.21E-10 |  |
| Fruit weight | Pp02 | 27741374 | C | 0.901361 | G | 0.0986395 | 9.02E-10 |  |
| Fruit weight | Pp02 | 27748184 | C | 0.906532 | T | 0.0934685 | 9.36E-09 |  |
| Fruit weight | Pp03 | 2812488 | A | 0.906874 | T | 0.0931264 | 9.11E-11 |  |
| Fruit weight | Pp03 | 2812491 | C | 0.908389 | T | 0.0916115 | 4.77E-09 |  |
| Fruit weight | Pp03 | 6194371 | T | 0.983796 | A | 0.0162037 | 4.57E-09 |  |
| Fruit weight | Pp03 | 7717077 | T | 0.988166 | A | 0.0118343 | 5.74E-09 |  |
| Fruit weight | Pp04 | 7906448 | T | 0.902985 | C | 0.0970149 | 5.74E-09 |  |
| Fruit weight | Pp04 | 7911525 | T | 0.913717 | C | 0.0862832 | 2.74E-08 |  |
| Fruit weight | Pp06 | 10995243 | C | 0.918919 | T | 0.0810811 | 4.83E-09 |  |
| Fruit weight | Pp06 | 27930715 | T | 0.884615 | G | 0.115385 | 4.08E-09 | *fw*, ref. 28 |
| Fruit weight | Pp07 | 9043163 | C | 0.988938 | T | 0.0110619 | 2.49E-08 |  |
| Fruit weight | Pp07 | 10173187 | G | 0.979616 | A | 0.0203837 | 2.60E-08 |  |
| Fruit weight | Pp07 | 10193627 | T | 0.897603 | C | 0.102397 | 3.52E-09 |  |
| Fruit weight | Pp07 | 10205251 | T | 0.894565 | C | 0.105435 | 9.11E-11 |  |
| Fruit weight | Pp07 | 10206371 | A | 0.887689 | G | 0.112311 | 8.72E-11 |  |
| Fruit weight | Pp07 | 10206448 | G | 0.901515 | A | 0.0984848 | 4.43E-10 |  |
| Fruit weight | Pp07 | 10332521 | T | 0.991202 | A | 0.00879765 | 5.74E-09 |  |
| Fruit weight | Pp07 | 11238927 | C | 0.891858 | A | 0.108142 | 8.72E-11 |  |
| Fruit weight | Pp08 | 1070082 | G | 0.896991 | A | 0.103009 | 4.57E-09 |  |
| Fruit weight | Pp08 | 1879783 | C | 0.989024 | T | 0.0109756 | 2.89E-09 |  |
| Fruit weight | Pp08 | 8731918 | A | 0.901496 | G | 0.0985037 | 1.50E-09 |  |
| Fruit weight | Pp08 | 8732984 | C | 0.914141 | T | 0.0858586 | 4.57E-09 |  |
| Fruit weight | Pp08 | 8733182 | T | 0.900491 | C | 0.0995086 | 4.57E-09 |  |
| Fruit weight | Pp08 | 8733205 | A | 0.901985 | G | 0.0980149 | 8.72E-11 |  |
| Fruit weight | Pp08 | 8733206 | T | 0.903226 | C | 0.0967742 | 2.01E-10 |  |
| Fruit weight | Pp08 | 8733390 | A | 0.893204 | G | 0.106796 | 8.72E-11 |  |
| Fruit weight | Pp08 | 8733508 | A | 0.91906 | G | 0.0809399 | 1.46E-08 |  |
| Fruit weight | Pp08 | 8733676 | C | 0.908629 | A | 0.0913706 | 4.57E-09 |  |
| Fruit weight | Pp08 | 8733905 | C | 0.895778 | G | 0.104222 | 8.72E-11 | *fw*, ref. 28 |
| Fruit weight | Pp08 | 8734046 | C | 0.902375 | G | 0.0976253 | 2.41E-09 |  |
| Fruit weight | Pp08 | 8734197 | A | 0.915365 | G | 0.0846354 | 8.72E-11 |  |
| Fruit weight | Pp08 | 8734327 | T | 0.904822 | C | 0.0951777 | 1.90E-09 |  |
| Fruit weight | Pp08 | 8734525 | T | 0.90736 | C | 0.0926396 | 4.57E-09 |  |
| Fruit weight | Pp08 | 8734616 | C | 0.909439 | G | 0.0905612 | 1.54E-10 |  |
| Fruit weight | Pp08 | 8734619 | A | 0.907828 | G | 0.0921717 | 2.66E-08 |  |
| Fruit weight | Pp08 | 8734963 | T | 0.905371 | C | 0.0946292 | 4.57E-09 |  |
| Fruit weight | Pp08 | 8735128 | T | 0.89467 | C | 0.10533 | 1.88E-09 |  |
| Fruit weight | Pp08 | 8735243 | A | 0.909429 | G | 0.0905707 | 8.72E-11 |  |
| Fruit weight | Pp08 | 8735309 | T | 0.901496 | C | 0.0985037 | 9.11E-11 |  |
| Fruit weight | Pp08 | 8735544 | T | 0.914787 | C | 0.085213 | 9.11E-11 |  |
| Fruit weight | Pp08 | 20537546 | T | 0.942935 | C | 0.0570652 | 5.74E-09 |  |
| Soluble solid content | Pp01 | 11459002 | A | 0.923077 | T | 0.0769231 | 1.30E-09 |  |
| Soluble solid content | Pp01 | 11482853 | C | 0.946319 | T | 0.053681 | 2.48E-09 |  |
| Soluble solid content | Pp01 | 11511656 | T | 0.975535 | C | 0.0244648 | 3.46E-09 |  |
| Soluble solid content | Pp01 | 11597100 | T | 0.962963 | A | 0.037037 | 1.01E-08 |  |
| Soluble solid content | Pp01 | 11599686 | A | 0.961095 | C | 0.0389049 | 1.21E-09 |  |
| Soluble solid content | Pp01 | 11606442 | G | 0.960411 | T | 0.0395894 | 1.79E-09 |  |
| Soluble solid content | Pp01 | 11613651 | G | 0.962099 | C | 0.0379009 | 1.37E-08 |  |
| Soluble solid content | Pp01 | 15076679 | G | 0.98368 | A | 0.0163205 | 2.58E-09 |  |
| Soluble solid content | Pp01 | 15715662 | G | 0.987032 | A | 0.0129683 | 6.08E-09 |  |
| Soluble solid content | Pp01 | 33203045 | T | 0.987633 | A | 0.0123675 | 1.58E-08 |  |
| Soluble solid content | Pp02 | 14008803 | G | 0.987382 | A | 0.0126183 | 2.83E-09 |  |
| Soluble solid content | Pp02 | 29042803 | A | 0.986607 | G | 0.0133929 | 2.38E-08 |  |
| Soluble solid content | Pp03 | 3919221 | G | 0.97929 | C | 0.0207101 | 3.84E-12 |  |
| Soluble solid content | Pp03 | 13470874 | G | 0.986842 | A | 0.0131579 | 2.21E-08 |  |
| Soluble solid content | Pp04 | 1851070 | C | 0.989706 | T | 0.0102941 | 3.44E-10 |  |
| Soluble solid content | Pp04 | 1851100 | A | 0.988372 | G | 0.0116279 | 1.89E-08 | *suc, glu, fru*, ref. 26 |
| Soluble solid content | Pp04 | 8866627 | T | 0.979365 | A | 0.0206349 | 2.47E-08 |  |
| Soluble solid content | Pp04 | 9917371 | G | 0.987952 | A | 0.0120482 | 2.35E-08 |  |
| Soluble solid content | Pp04 | 9956708 | A | 0.987578 | C | 0.0124224 | 5.63E-09 | *SSC*, ref. 28 |
| Soluble solid content | Pp05 | 1436302 | G | 0.986322 | A | 0.0136778 | 8.27E-09 |  |
| Soluble solid content | Pp05 | 1893432 | A | 0.988889 | G | 0.0111111 | 4.26E-12 | *SSC*, ref. 27 |
| Soluble solid content | Pp06 | 7824892 | A | 0.981544 | T | 0.0184564 | 9.27E-10 |  |
| Soluble solid content | Pp06 | 7850440 | T | 0.987055 | A | 0.012945 | 5.67E-10 |  |
| Soluble solid content | Pp06 | 7888813 | T | 0.975146 | C | 0.0248538 | 3.78E-09 |  |
| Soluble solid content | Pp06 | 7888826 | G | 0.973294 | A | 0.0267062 | 8.71E-10 |  |
| Soluble solid content | Pp06 | 7888830 | G | 0.973294 | T | 0.0267062 | 7.94E-10 |  |
| Soluble solid content | Pp06 | 8899827 | C | 0.988679 | T | 0.0113208 | 1.79E-09 |  |
| Soluble solid content | Pp06 | 8899828 | T | 0.988679 | C | 0.0113208 | 1.79E-09 |  |
| Soluble solid content | Pp06 | 16252217 | C | 0.988959 | T | 0.011041 | 7.51E-10 |  |
| Soluble solid content | Pp06 | 23352235 | G | 0.989583 | A | 0.0104167 | 1.70E-10 |  |
| Soluble solid content | Pp07 | 13671272 | A | 0.98773 | G | 0.0122699 | 3.65E-09 |  |
| Soluble solid content | Pp08 | 3419737 | C | 0.98505 | T | 0.0149502 | 1.51E-08 |  |
| Soluble solid content | Pp08 | 6821483 | C | 0.989264 | T | 0.0107362 | 4.47E-11 |  |
| Soluble solid content | Pp08 | 7734847 | C | 0.988806 | G | 0.011194 | 2.64E-09 |  |
| Soluble solid content | Pp08 | 11566080 | C | 0.984894 | T | 0.0151057 | 4.90E-09 |  |

**Table S14** SNPs associated with chilling requirement.

| Trait | Chr. | Position | Major allele | Major allele frequency | Minor allele | Minor allele frequency | *P* value | Known loci |
| --- | --- | --- | --- | --- | --- | --- | --- | --- |
| Chilling requirement | Pp01 | 43711400 | A | 0.986188 | G | 0.0138122 | 3.59E-08 | *qCR1*, ref. 38 and 39 |
| Chilling requirement | Pp01 | 43713878 | C | 0.982044 | G | 0.0179558 | 4.19E-10 |  |
| Chilling requirement | Pp01 | 43714809 | C | 0.980663 | G | 0.019337 | 6.56E-10 |  |
| Chilling requirement | Pp01 | 43717948 | C | 0.977901 | T | 0.0220994 | 3.23E-08 |  |
| Chilling requirement | Pp01 | 43731422 | G | 0.976519 | A | 0.0234807 | 2.80E-08 |  |
| Chilling requirement | Pp01 | 43733491 | T | 0.982044 | G | 0.0179558 | 8.48E-09 |  |
| Chilling requirement | Pp01 | 43736851 | G | 0.976519 | A | 0.0234807 | 2.80E-08 |  |
| Chilling requirement | Pp01 | 43741862 | G | 0.977901 | T | 0.0220994 | 3.23E-08 |  |
| Chilling requirement | Pp01 | 43742220 | G | 0.977901 | T | 0.0220994 | 3.23E-08 |  |
| Chilling requirement | Pp01 | 45230856 | T | 0.984807 | C | 0.0151934 | 1.32E-08 |  |
| Chilling requirement | Pp01 | 45838821 | C | 0.983425 | A | 0.0165746 | 2.42E-08 |  |
| Chilling requirement | Pp01 | 45838823 | T | 0.983425 | C | 0.0165746 | 2.42E-08 |  |
| Chilling requirement | Pp01 | 45838982 | A | 0.984807 | T | 0.0151934 | 1.32E-08 |  |
| Chilling requirement | Pp01 | 45839188 | G | 0.983425 | C | 0.0165746 | 2.42E-08 |  |
| Chilling requirement | Pp01 | 45839338 | C | 0.983425 | G | 0.0165746 | 2.42E-08 |  |
| Chilling requirement | Pp04 | 6137547 | G | 0.984807 | A | 0.0151934 | 2.52E-09 |  |
| Chilling requirement | Pp07 | 366416 | G | 0.976519 | A | 0.0234807 | 2.22E-09 |  |
| Chilling requirement | Pp07 | 370886 | T | 0.984807 | C | 0.0151934 | 5.56E-10 |  |
| Chilling requirement | Pp07 | 370900 | A | 0.984807 | G | 0.0151934 | 5.56E-10 |  |
| Chilling requirement | Pp07 | 370936 | C | 0.984807 | A | 0.0151934 | 1.24E-09 |  |
| Chilling requirement | Pp07 | 7410223 | T | 0.959945 | G | 0.0400552 | 1.98E-08 |  |
| Chilling requirement | Pp07 | 8216840 | T | 0.969613 | A | 0.0303867 | 3.70E-10 |  |
| Chilling requirement | Pp07 | 8217300 | A | 0.972376 | G | 0.0276243 | 7.24E-09 |  |
| Chilling requirement | Pp07 | 14956855 | A | 0.980663 | G | 0.019337 | 1.82E-08 |  |
| Chilling requirement | Pp07 | 14956867 | T | 0.980663 | G | 0.019337 | 1.82E-08 |  |
| Chilling requirement | Pp07 | 15001268 | G | 0.979282 | C | 0.0207182 | 2.00E-09 |  |
| Chilling requirement | Pp07 | 16197391 | G | 0.984807 | A | 0.0151934 | 1.90E-08 | *qCR7*, ref. 38 |
| Chilling requirement | Pp07 | 16203974 | C | 0.987569 | G | 0.0124309 | 8.71E-09 |  |
| Chilling requirement | Pp07 | 16229078 | C | 0.987569 | A | 0.0124309 | 8.71E-09 |  |
| Chilling requirement | Pp07 | 16231443 | G | 0.984807 | A | 0.0151934 | 1.90E-08 |  |
| Chilling requirement | Pp07 | 16234656 | G | 0.984807 | A | 0.0151934 | 1.90E-08 |  |
| Chilling requirement | Pp07 | 22058699 | G | 0.986188 | C | 0.0138122 | 9.01E-10 |  |
| Chilling requirement | Pp08 | 5674968 | A | 0.941989 | T | 0.058011 | 8.91E-09 |  |
| Chilling requirement | Pp08 | 5674971 | C | 0.941989 | T | 0.058011 | 8.91E-09 |  |
| Chilling requirement | Pp08 | 7789510 | A | 0.925414 | G | 0.0745856 | 6.10E-09 |  |
| Chilling requirement | Pp08 | 7814722 | G | 0.924033 | A | 0.0759669 | 3.24E-08 |  |
| Chilling requirement | Pp08 | 7827493 | T | 0.95442 | C | 0.0455801 | 2.03E-08 |  |
| Chilling requirement | Pp08 | 7837960 | A | 0.987569 | G | 0.0124309 | 2.20E-10 |  |
| Chilling requirement | Pp08 | 7837965 | C | 0.987569 | T | 0.0124309 | 2.20E-10 |  |
| Chilling requirement | Pp08 | 7837974 | A | 0.987569 | G | 0.0124309 | 2.20E-10 |  |
| Chilling requirement | Pp08 | 7837992 | T | 0.986188 | C | 0.0138122 | 1.76E-09 |  |
| Chilling requirement | Pp08 | 7847187 | T | 0.924033 | C | 0.0759669 | 1.82E-08 |  |
| Chilling requirement | Pp08 | 8279279 | G | 0.959945 | A | 0.0400552 | 3.83E-08 | *qCR8*, ref. 38 |
| Chilling requirement | Pp08 | 12381657 | G | 0.964088 | A | 0.0359116 | 2.26E-10 |  |

**Table S19** Shared selective sweeps between western and eastern improved groups.

| **Chromosome** | **Start (bp)** | **End (bp)** |
| --- | --- | --- |
| Pp01 | 670001 | 870000 |
| Pp01 | 8510001 | 8630000 |
| Pp01 | 9550001 | 9680000 |
| Pp01 | 9850001 | 9980000 |
| Pp01 | 11400001 | 11510000 |
| Pp01 | 15500001 | 15710000 |
| Pp01 | 26300001 | 26310000 |
| Pp01 | 26150001 | 26300000 |
| Pp01 | 39100001 | 39360000 |
| Pp02 | 14280001 | 14480000 |
| Pp02 | 17880001 | 18000000 |
| Pp02 | 20170001 | 20240000 |
| Pp02 | 20250001 | 20550000 |
| Pp02 | 27140001 | 27460000 |
| Pp02 | 27460001 | 27670000 |
| Pp03 | 11020001 | 11120000 |
| Pp03 | 11880001 | 11990000 |
| Pp03 | 13920001 | 14240000 |
| Pp03 | 24240001 | 24410000 |
| Pp04 | 1620001 | 1990000 |
| Pp04 | 2090001 | 2270000 |
| Pp04 | 7590001 | 7870000 |
| Pp04 | 16170001 | 16430000 |
| Pp05 | 100001 | 240000 |
| Pp05 | 1500001 | 1700000 |
| Pp05 | 1740001 | 2000000 |
| Pp05 | 2300001 | 2720000 |
| Pp05 | 2740001 | 2930000 |
| Pp05 | 3790001 | 4080000 |
| Pp05 | 10730001 | 10860000 |
| Pp05 | 12690001 | 12790000 |
| Pp05 | 14950001 | 15100000 |
| Pp05 | 15110001 | 15160000 |
| Pp05 | 15500001 | 15620000 |
| Pp05 | 15660001 | 15830000 |
| Pp05 | 15850001 | 16480000 |
| Pp05 | 17500001 | 17670000 |
| Pp05 | 17720001 | 17860000 |
| Pp05 | 18010001 | 18070000 |
| Pp05 | 17880001 | 18000000 |
| Pp05 | 18250001 | 18370000 |
| Pp06 | 5600001 | 5950000 |
| Pp06 | 6000001 | 6240000 |
| Pp06 | 6830001 | 6980000 |
| Pp06 | 9060001 | 9200000 |
| Pp06 | 9540001 | 9910000 |
| Pp06 | 18170001 | 18310000 |
| Pp06 | 30110001 | 30340000 |
| Pp06 | 30400001 | 30750000 |
| Pp07 | 5500001 | 5610000 |
| Pp07 | 13300001 | 13490000 |
| Pp07 | 14580001 | 14680000 |
| Pp07 | 18540001 | 18810000 |
| Pp07 | 20200001 | 20430000 |
| Pp07 | 21400001 | 21860000 |
| Pp08 | 20001 | 130000 |
| Pp08 | 21840001 | 22160000 |

**Table S20 Eastern specific improvement sweeps.**

| **Chromosome** | **Start (bp)** | **End (bp)** |
| --- | --- | --- |
| Pp01 | 5310001 | 5430000 |
| Pp01 | 5490001 | 5590000 |
| Pp01 | 6400001 | 6570000 |
| Pp01 | 6710001 | 6810000 |
| Pp01 | 6830001 | 6950000 |
| Pp01 | 26310000 | 26420000 |
| Pp01 | 26430001 | 26620000 |
| Pp01 | 42050001 | 42170000 |
| Pp01 | 44430001 | 44540000 |
| Pp01 | 46560001 | 46940000 |
| Pp01 | 47010001 | 47110000 |
| Pp02 | 640001 | 750000 |
| Pp02 | 750001 | 1000000 |
| Pp02 | 19600001 | 19710000 |
| Pp02 | 20240000 | 20250001 |
| Pp02 | 27460000 | 27460001 |
| Pp02 | 27670000 | 27720000 |
| Pp03 | 2340001 | 2470000 |
| Pp03 | 5910001 | 6220000 |
| Pp03 | 11300001 | 11440000 |
| Pp03 | 11990000 | 12000000 |
| Pp03 | 12520001 | 12670000 |
| Pp03 | 13910001 | 13920001 |
| Pp03 | 18270001 | 18540000 |
| Pp03 | 18810001 | 19060000 |
| Pp03 | 19670001 | 19940000 |
| Pp03 | 20020001 | 20170000 |
| Pp03 | 21810001 | 21930000 |
| Pp03 | 24010001 | 24160000 |
| Pp03 | 24420001 | 24550000 |
| Pp03 | 24630001 | 24820000 |
| Pp03 | 26500001 | 26640000 |
| Pp04 | 50001 | 400000 |
| Pp04 | 1550001 | 1620001 |
| Pp04 | 2060001 | 2090001 |
| Pp04 | 2270000 | 2310000 |
| Pp04 | 3250001 | 3440000 |
| Pp04 | 7870000 | 7930000 |
| Pp04 | 11480001 | 11590000 |
| Pp05 | 50001 | 100001 |
| Pp05 | 240000 | 260000 |
| Pp05 | 310001 | 440000 |
| Pp05 | 2000000 | 2040000 |
| Pp05 | 2290001 | 2300001 |
| Pp05 | 2720000 | 2740001 |
| Pp05 | 2930000 | 2940000 |
| Pp05 | 4080000 | 4110000 |
| Pp05 | 4320001 | 4600000 |
| Pp05 | 7850001 | 7980000 |
| Pp05 | 12680001 | 12690001 |
| Pp05 | 12790000 | 12800000 |
| Pp05 | 13100001 | 13230000 |
| Pp05 | 14940001 | 14950001 |
| Pp05 | 15160000 | 15210000 |
| Pp05 | 15220001 | 15370000 |
| Pp05 | 15470001 | 15500001 |
| Pp05 | 17670000 | 17690000 |
| Pp05 | 17860000 | 17880001 |
| Pp05 | 18070000 | 18110000 |
| Pp05 | 18230001 | 18250001 |
| Pp05 | 18370000 | 18380000 |
| Pp06 | 4810001 | 4980000 |
| Pp06 | 5590001 | 5600001 |
| Pp06 | 8880001 | 9060001 |
| Pp06 | 9200000 | 9210000 |
| Pp06 | 10730001 | 10880000 |
| Pp06 | 18050001 | 18150000 |
| Pp06 | 30340000 | 30400001 |
| Pp06 | 30750000 | 30760000 |
| Pp07 | 1610001 | 1760000 |
| Pp07 | 5480001 | 5500001 |
| Pp07 | 5610000 | 5640000 |
| Pp07 | 9320001 | 9470000 |
| Pp07 | 10780001 | 10950000 |
| Pp07 | 13290001 | 13300001 |
| Pp07 | 13490000 | 13540000 |
| Pp07 | 14190001 | 14300000 |
| Pp07 | 14570001 | 14580001 |
| Pp07 | 14680000 | 14710000 |
| Pp07 | 18470001 | 18540001 |
| Pp07 | 18810000 | 18820000 |
| Pp08 | 1820001 | 2020000 |
| Pp08 | 2520001 | 2720000 |
| Pp08 | 2720001 | 2830000 |
| Pp08 | 2830001 | 2980000 |
| Pp08 | 3270001 | 3450000 |
| Pp08 | 3930001 | 4110000 |
| Pp08 | 5110001 | 5280000 |
| Pp08 | 6650001 | 6760000 |
| Pp08 | 20890001 | 21000000 |

**Table S21** Western group specific improvement sweeps.

| **Chromosome** | **Start (bp)** | **End (bp)** |
| --- | --- | --- |
| Pp01 | 5600001 | 5810000 |
| Pp01 | 8490001 | 8510001 |
| Pp01 | 9080001 | 9180000 |
| Pp01 | 9990001 | 10160000 |
| Pp01 | 11170001 | 11340000 |
| Pp01 | 11370001 | 11400001 |
| Pp01 | 11510000 | 11520000 |
| Pp01 | 14250001 | 14680000 |
| Pp01 | 15450001 | 15500001 |
| Pp01 | 15710000 | 15840000 |
| Pp01 | 17650001 | 17750000 |
| Pp01 | 18660001 | 18860000 |
| Pp01 | 18890001 | 19330000 |
| Pp01 | 19380001 | 19480000 |
| Pp01 | 22170001 | 22590000 |
| Pp01 | 22600001 | 22700000 |
| Pp01 | 24200001 | 24300000 |
| Pp01 | 24650001 | 24850000 |
| Pp01 | 24900001 | 25020000 |
| Pp01 | 25270001 | 25490000 |
| Pp01 | 25580001 | 25710000 |
| Pp01 | 26030001 | 26150001 |
| Pp01 | 26300000 | 26300001 |
| Pp01 | 32520001 | 32630000 |
| Pp01 | 39360000 | 39370000 |
| Pp01 | 42250001 | 42350000 |
| Pp02 | 11210001 | 11320000 |
| Pp02 | 14480000 | 14490000 |
| Pp02 | 18000000 | 18310000 |
| Pp02 | 18450001 | 18620000 |
| Pp02 | 20090001 | 20170001 |
| Pp02 | 20550000 | 20560000 |
| Pp02 | 22540001 | 23310000 |
| Pp02 | 23370001 | 23690000 |
| Pp02 | 24180001 | 24350000 |
| Pp02 | 24890001 | 25020000 |
| Pp02 | 25270001 | 25460000 |
| Pp02 | 25980001 | 26090000 |
| Pp02 | 26760001 | 27140001 |
| Pp02 | 29400001 | 30040000 |
| Pp03 | 11120000 | 11140000 |
| Pp03 | 24220001 | 24240001 |
| Pp04 | 16430000 | 16460000 |
| Pp05 | 1440001 | 1500001 |
| Pp05 | 1700000 | 1740001 |
| Pp05 | 10720001 | 10730001 |
| Pp05 | 10860000 | 10870000 |
| Pp05 | 12210001 | 12330000 |
| Pp05 | 15100000 | 15110001 |
| Pp05 | 15620000 | 15640000 |
| Pp05 | 15840001 | 15850001 |
| Pp05 | 16480000 | 16490000 |
| Pp05 | 18000000 | 18010001 |
| Pp06 | 4220001 | 4340000 |
| Pp06 | 5180001 | 5350000 |
| Pp06 | 5950000 | 5970000 |
| Pp06 | 6740001 | 6830001 |
| Pp06 | 9220001 | 9400000 |
| Pp06 | 9520001 | 9540001 |
| Pp06 | 21690001 | 21790000 |
| Pp07 | 17570001 | 17750000 |
| Pp08 | 10001 | 20001 |
| Pp08 | 130000 | 140000 |
| Pp08 | 21770001 | 21840001 |
| Pp08 | 22160000 | 22270000 |
| Pp08 | 22370001 | 22480000 |

**Table S22** Best fitting parameters for the two-population model for cultivated and wild peach groups in the demographic analysis. We estimated the ancient population size by the formula 4*N*e ×*μ*× *L*=*θ*, where *μ* is the mutation rate (7.77×10^-9^), *L* is the generation time (3) and*θ* is the genetic diversity (set to 3.5×10^-3^).

|  | Model | Parameters | Scaled^a^ |
| --- | --- | --- | --- |
| Population size at domestication | nub | 0.0028 | 210 |
| Final size of population | nuf | 0.1749 | 13,130 |
| Duration of bottleneck (year) | Tb | 0.00156 | 117 |
| Time since bottleneck to final population (year) | Tf | 0.1148 | 8,618 |
| Final size of ancestor | nu1 | 0.1053 | 7,905 |

^a^Values are calculated by multiplying 2*N*e (7.5075×10^4^) and the best fitting parameters

**Table S23** Primers used in this study.

| **Primer ID** | **Sequences** |
| --- | --- |
| SVP-F-CLONE | ATGACGAGGAGGAAAATCCAGA |
| SVP-R-CLONE | GGCAAGCTTTTCCTAACGGGATATGA |
| SVP-F-qRT-PCR | GATTGACAACACAACGGCGA |
| SVP-R-qRT-PCR | AAGTGTCAGAGCTGTCGTGG |
| actin-F | GTTATTCTTCATCGGCGTCTTCG |
| actin-R | CTTCACCATTCCAGTTCCATTGTC |
| Prupe.4G150100-F-qRT-PCR | CCTCAAAGCCAGTACCCCAG |
| Prupe.4G150100-R-qRT-PCR | TTTGGGAAGTGGGTGTTGCT |
