# Supplementary material for: Genomic analyses of an extensive collection of wild and cultivated accessions provide new insights into peach breeding history
Source: Genome Biol. 2019 Feb 21;20:36. doi: 10.1186/s13059-019-1648-9 (PMC6383288; doi:10.1186/s13059-019-1648-9)
Supplement: Supplementary file 3 — Figure S1. Genetic diversity of the 480 peach accessions. Figure S2. Manhattan plot and QQ plot of genome-wide association studies on seven important agronomic traits. Figure S3. A candidate 70.5-kb deletion underlying fruit texture. Figure S4. Phenetic neighbor-joining tree of 480 peach accessions constructed using PHYLIP with 100 bootstrap replicates. Figure S5. Population structure of the 480 peach accessions. Figure S6. Phenotypes and nucleotide diversity changes during peach improvement and domestication. Figure S7. Genome-wide association studies (GWAS) of SSC and fruit weight. Figure S8. Regional LD decay measured by R2 at the 20.0–30.0-Mb region of chromosome 2. Figure S9. Total organic acids in domesticated peaches and wild relatives. Figure S10. Genome-wide association study of total phenolic content for peach. Figure S11. Expression profile of a sugar transport gene, Prupe.4G037800, during fruit development in peach. Figure S12. A candidate gene for SSC associated with increase of fruit taste during domestication. Figure S13. Distribution of chilling requirement in 371 peach accessions. Figure S14. Manhattan plot (left) and QQ plot (right) of GWAS for chilling requirement (CR) by classifying accessions into low CR and non-low CR. Figure S15. Comparison of flowering times between Arabidopsis overexpressing of peach SVP gene (OE) and the wild type (WT) Arabidopsis. Figure S16. A PCR-based marker for low CR peach identification based on the results of CR GWAS. Figure S17. Comparison of improvement sweeps between eastern and western improved groups. Figure S18. Shared and private SVs among three callers. Figure S19. Distribution of genotype missing rate for 480 peach accessions. Figure S20. Demographic model for peach domestication and spread. Figure S21. Shared domestication and improvement sweeps identified by ROD and XP-CLR methods. (PDF 2514 kb) [file 13059_2019_1648_MOESM3_ESM.pdf]

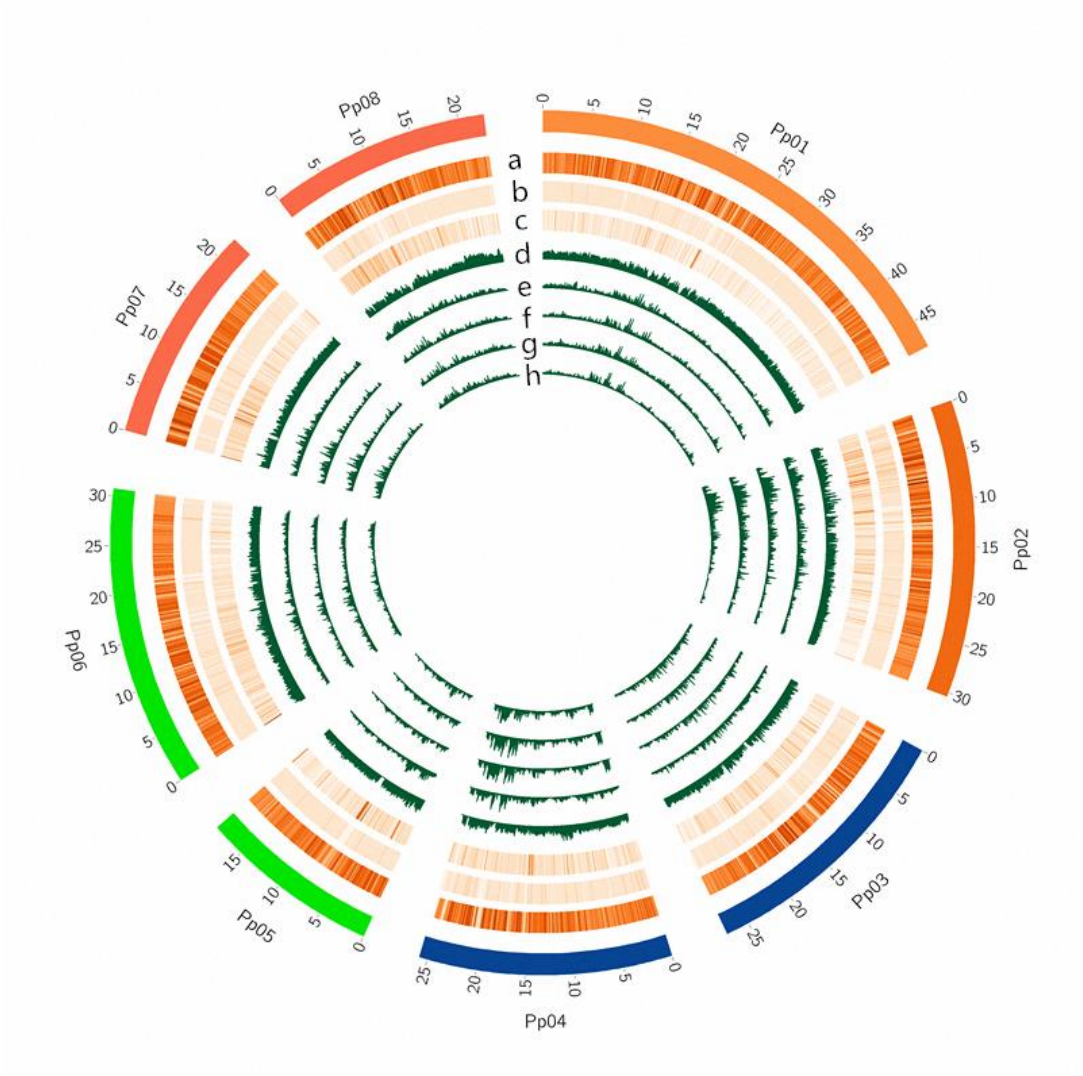

Figure S1 Genetic diversity of the 480 peach accessions. The outer ring indicates the eight peach chromosomes. The inner eight rings show the variation density (**a**-**c**) and nucleotide diversity for each group (**d**-**h**). SNP, INDEL, and SV densities were counted in 100-kb non-overlapping windows. The nucleotide diversity (π) was calculated using 100-kb sliding windows with a step size of 10 kb. (**a**) SNP density. (**b**) INDEL density. (**c**) SV density. (**d**) Nucleotide diversity of the wild group. (**e**) Nucleotide diversity of the landrace group. (**f**) Nucleotide diversity of the improved group. (**g**) Nucleotide diversity of the eastern improved group. (**h**) Nucleotide diversity of the western improved group.

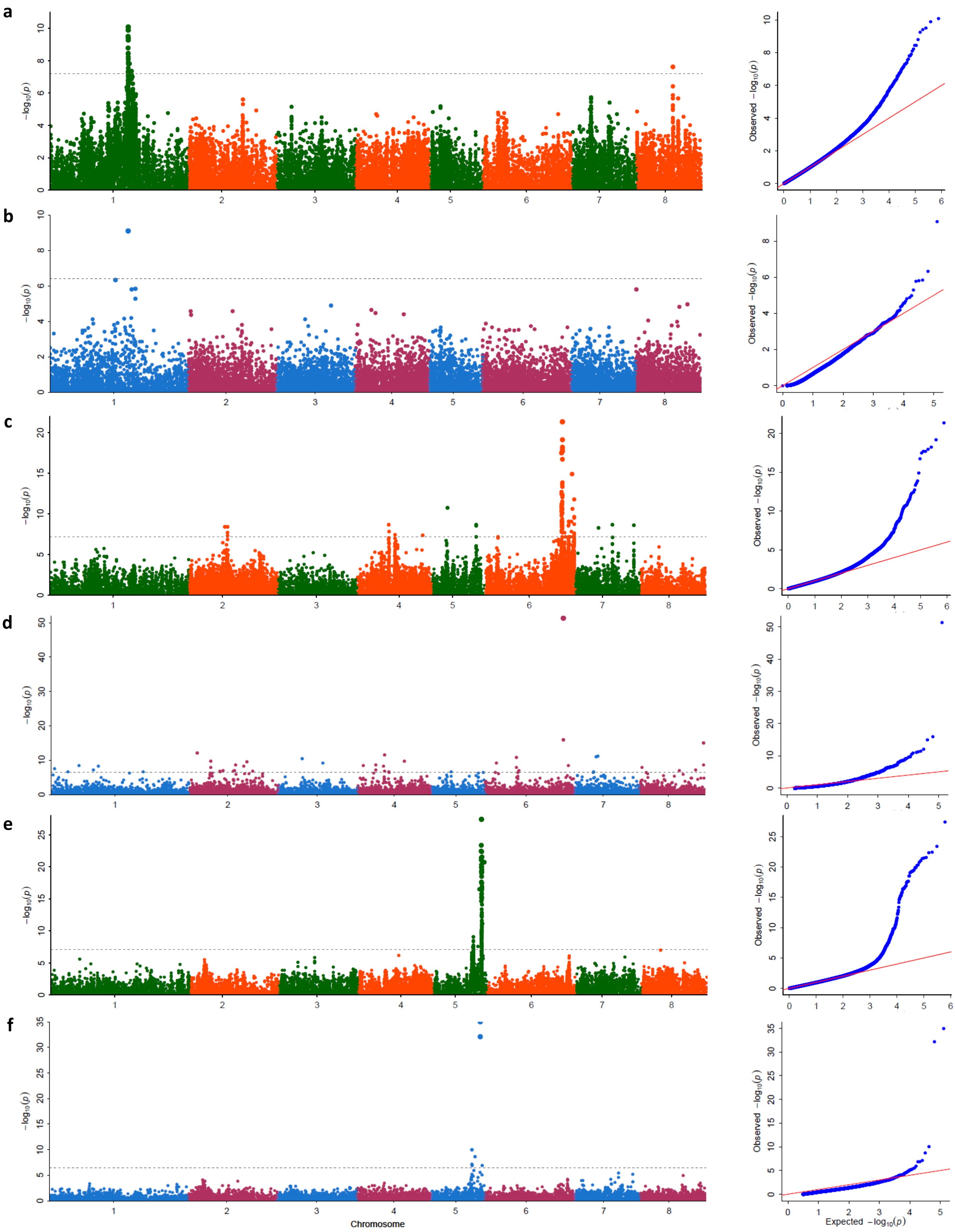
a
b
c
d
e
f
Chromosome
Expected −log10(p)
Observed −log10(p)
−log10(p)

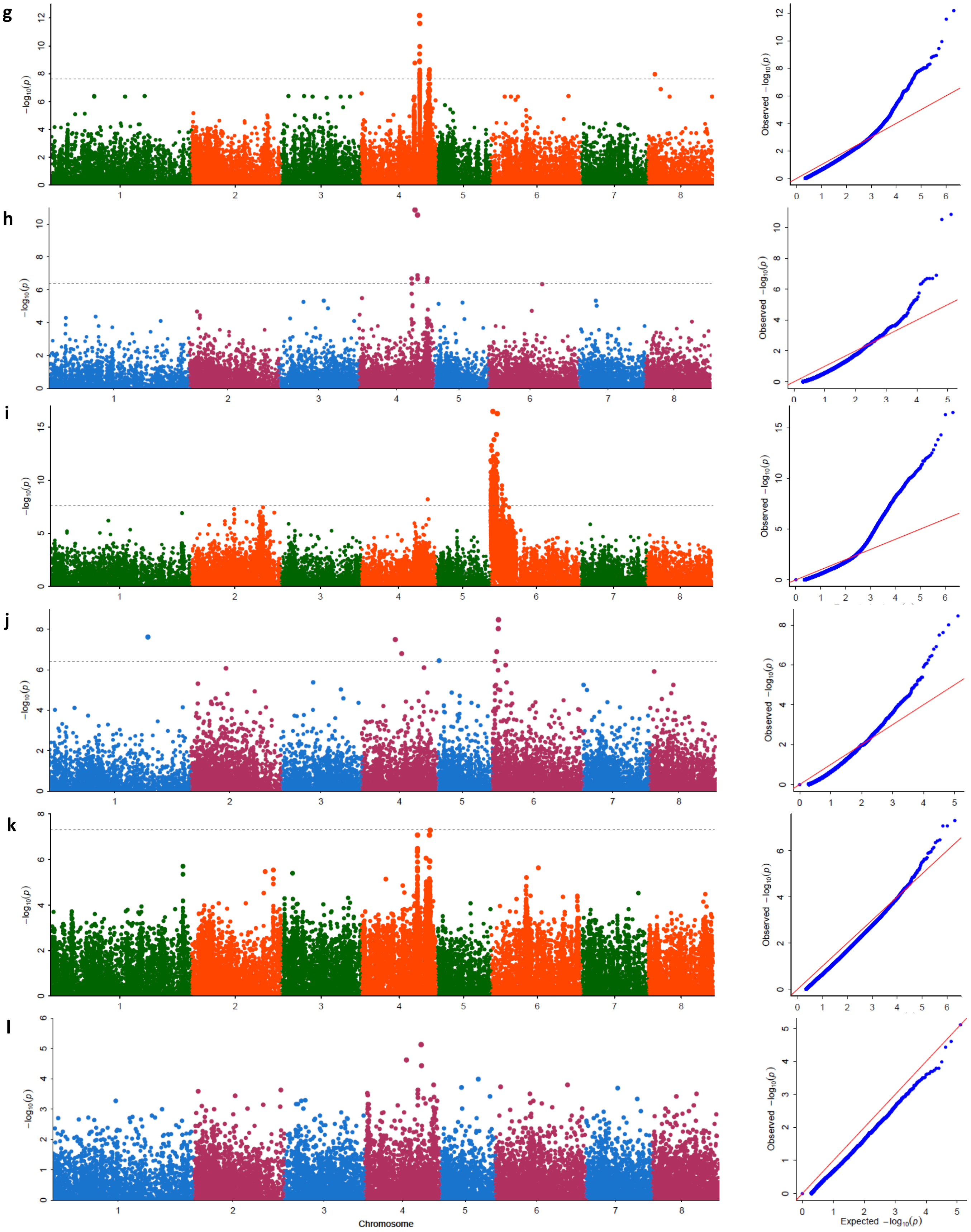
g
h
i
j
k
l
−log10(p)
Chromosome
Observed −log10(p)
Expected −log10(p)

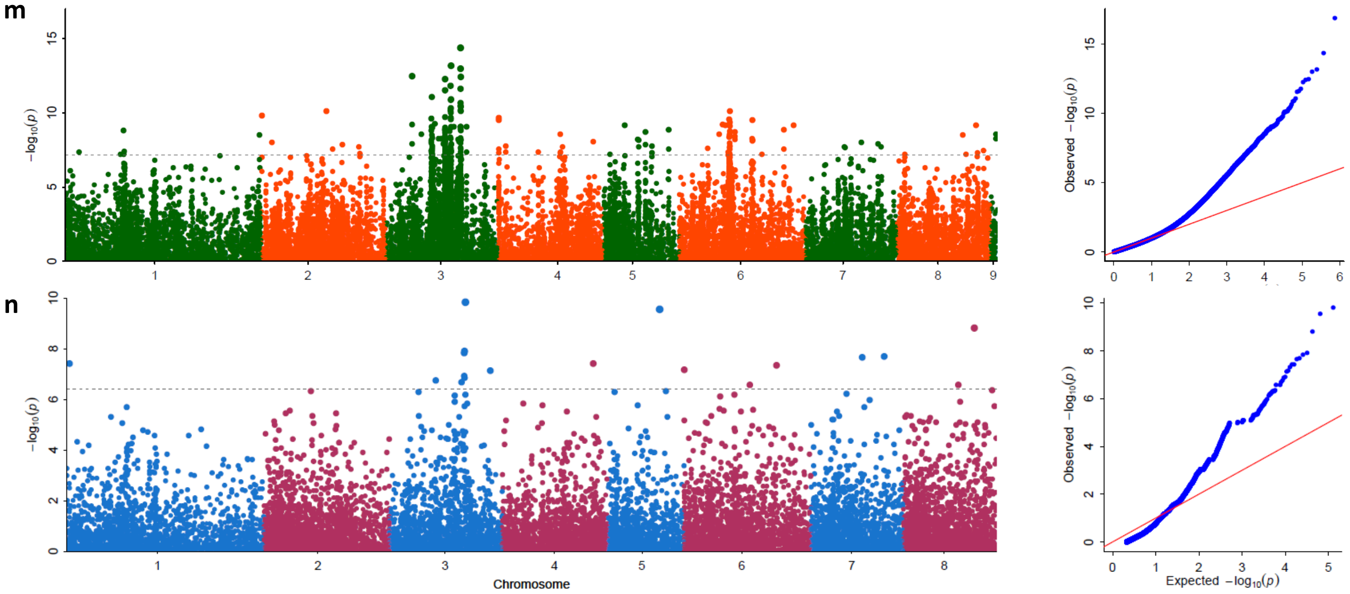

Figure S2 Manhattan plot and QQ plot of genome-wide association studies on seven important agronomic traits. (**a** and **b**) Flesh color (yellow/white). (**c** and **d**) Fruit shape (flat/round). (**e** and **f**) Nectarine (fruit hairness). (**g** and **h**) Fruit texture (melting/non-melting). (**i** and **j**) Male sterility (male sterility /male fertility). (**k** and **l**) Flesh adhesion (cling/free stone). (**m** and **n**) Fruit skin color (red/non-red skin). The horizontal dashed lines indicate the Bonferroni-corrected significance threshold of GWAS (0.05). GWAS in **a**, **c**, **e**, **g**, **i**, **k**, and **m** were performed using SNPs. GWAS in **b**, **d**, **f**, **h**, **j**, **l**, and **n** were performed using SVs.

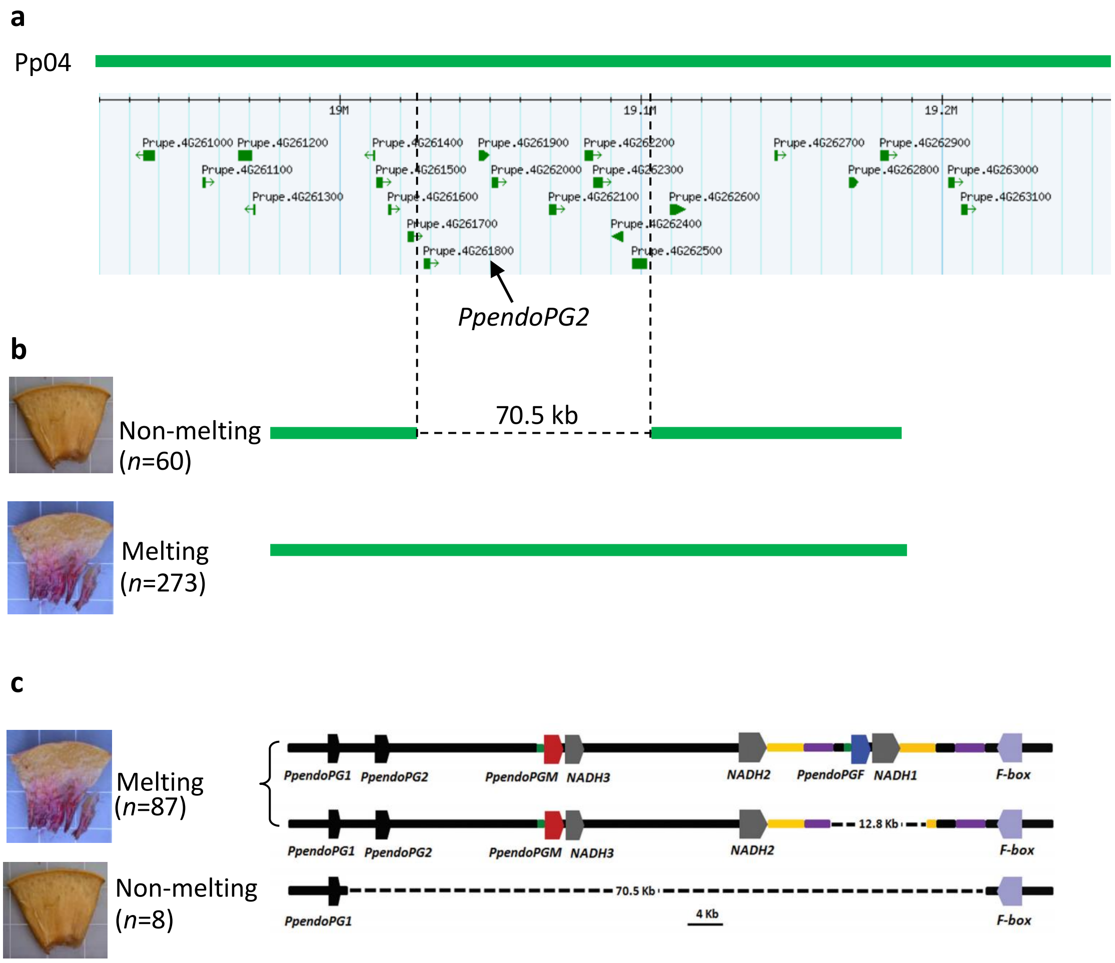

Figure S3 A candidate 70.5-kb deletion underlying fruit texture. (**a**) Gene model at 18.92-19.25 Mb on chromosome 4. (**b**) A 70.5-kb deletion associated with fruit texture and genotypes for non-melting and melting peaches. (**c**) Genetic model for fruit texture in a previous study (Gu et al, 2016; ref. 16). The numbers in bracket in (**b**) and (**c**) indicate the sample numbers.

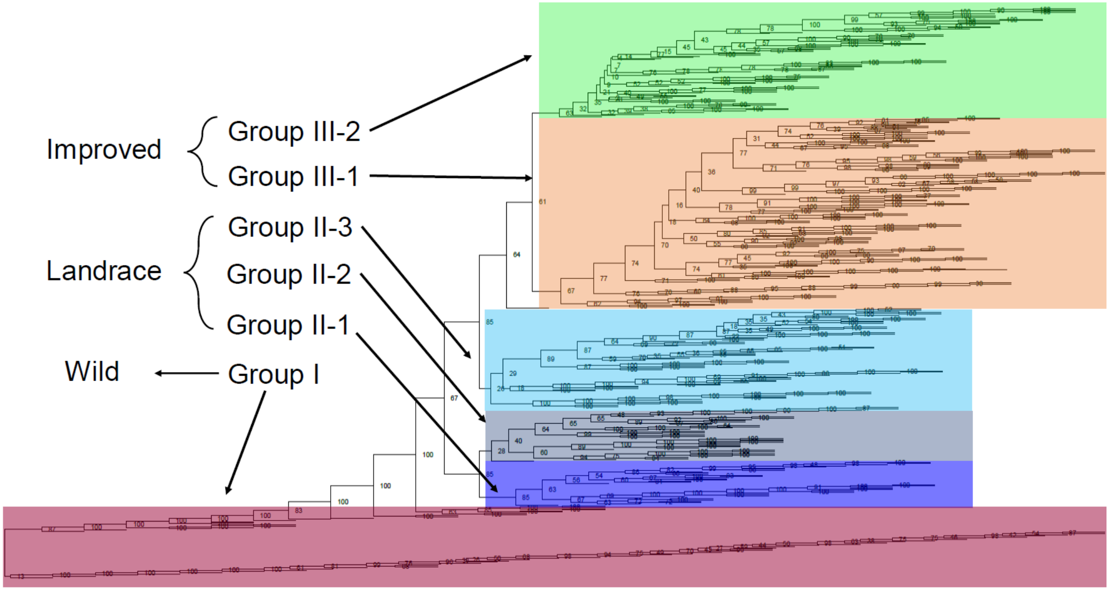

Figure S4 Phenetic neighbor-joining tree of 480 peach accessions constructed using PHYLIP with 100 bootstrap replicates. The six group was highlighted using different colors. The tree in the SVG format is available in the figshare database: https://figshare.com/articles/Phenetic_NJ-tree_of_480_peach_accessions/7241120

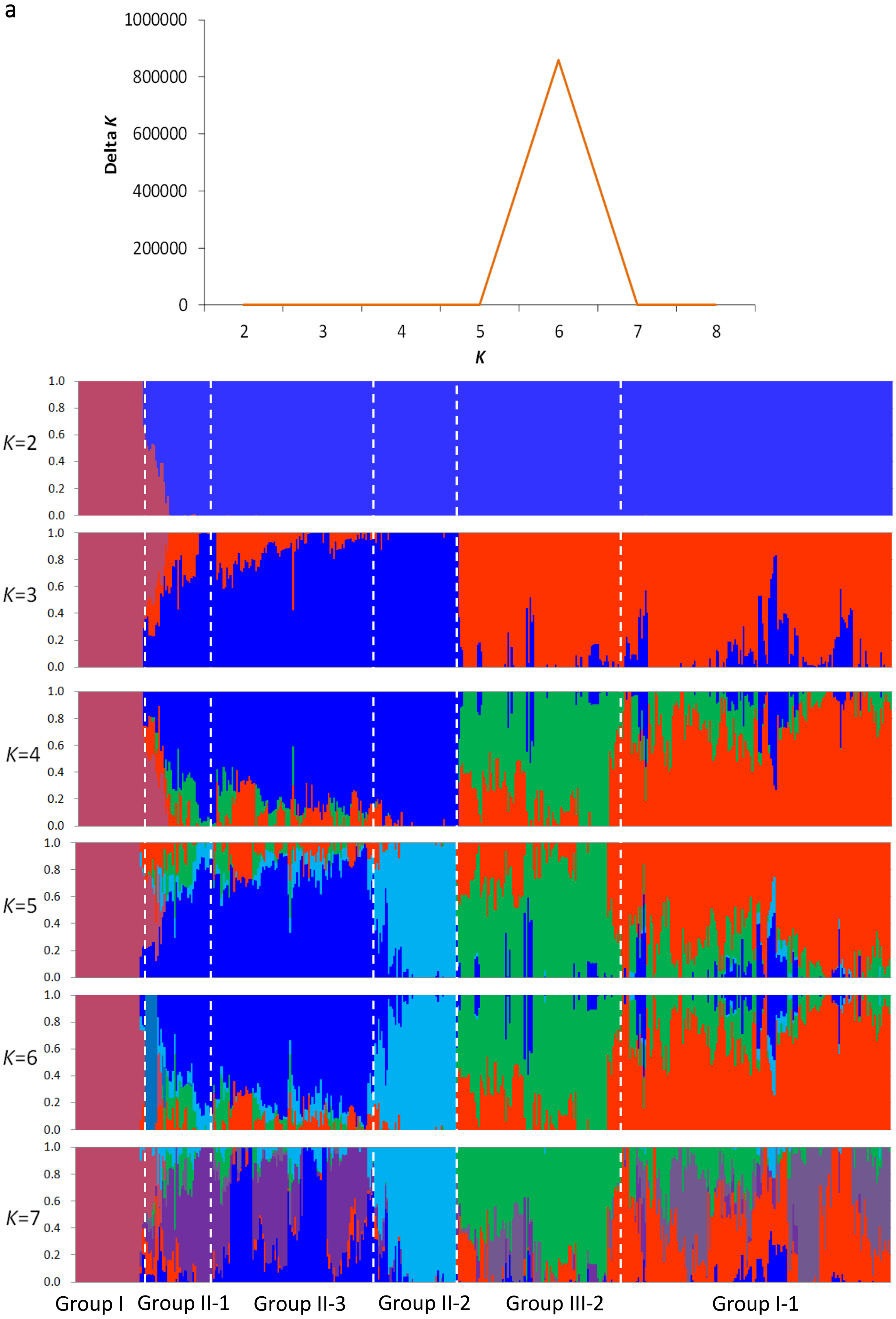

Figure S5 Population structure of the 480 peach accessions. (**a**) Estimation of delta *K*. *K*=6 was the best K value. (**b**) Population structure for 480 accessions by increasing *K* from 2 to 7.

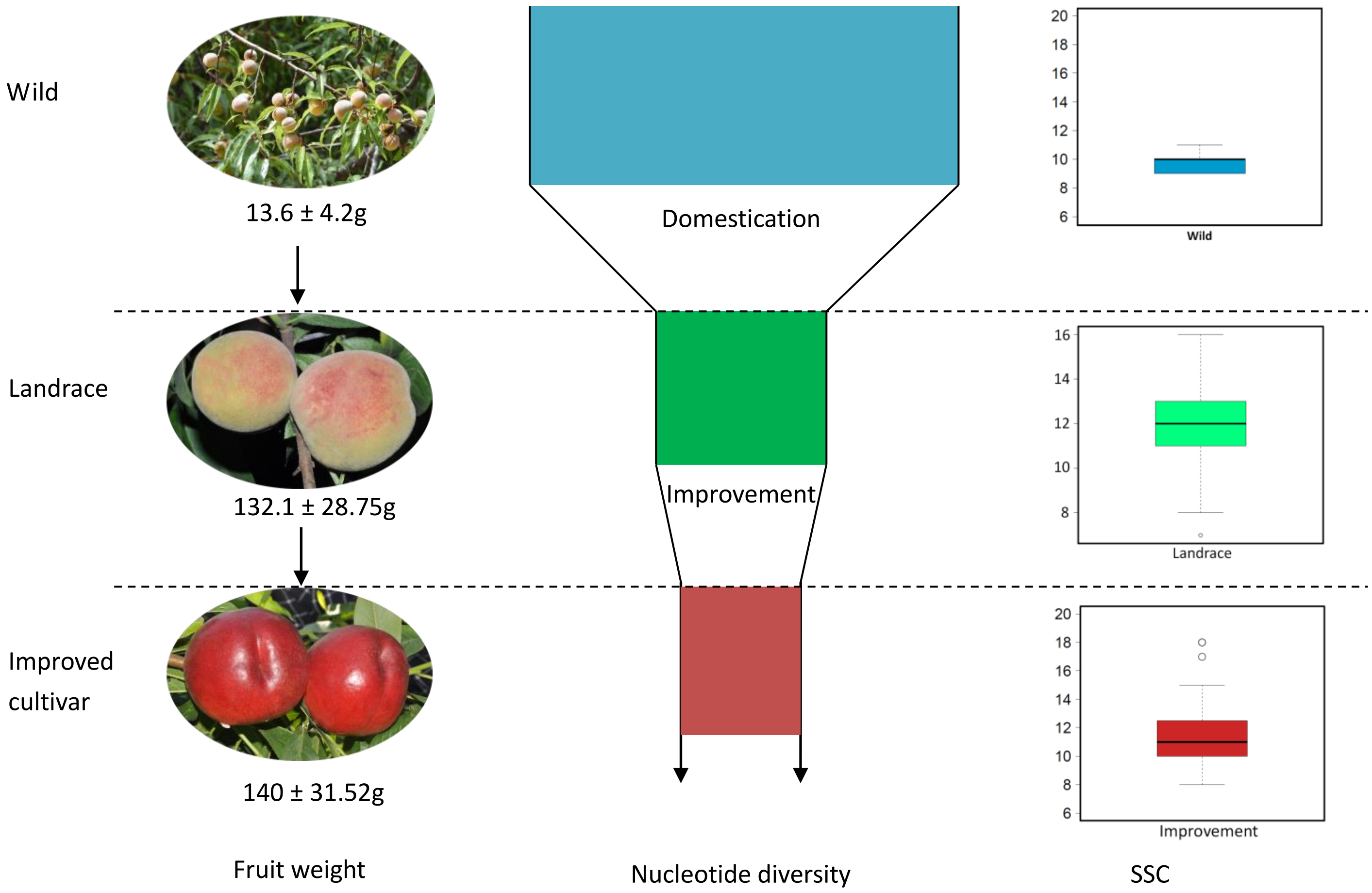

Figure S6 Phenotypes and nucleotide diversity changes during peach improvement and domestication. Morphology of wild, landrace and improved peaches is shown in the left with fruit weight below each photo. Nucleotide diversity represented by the width of each box was shown in the middle column. Box plots of SSC are shown in the right column.

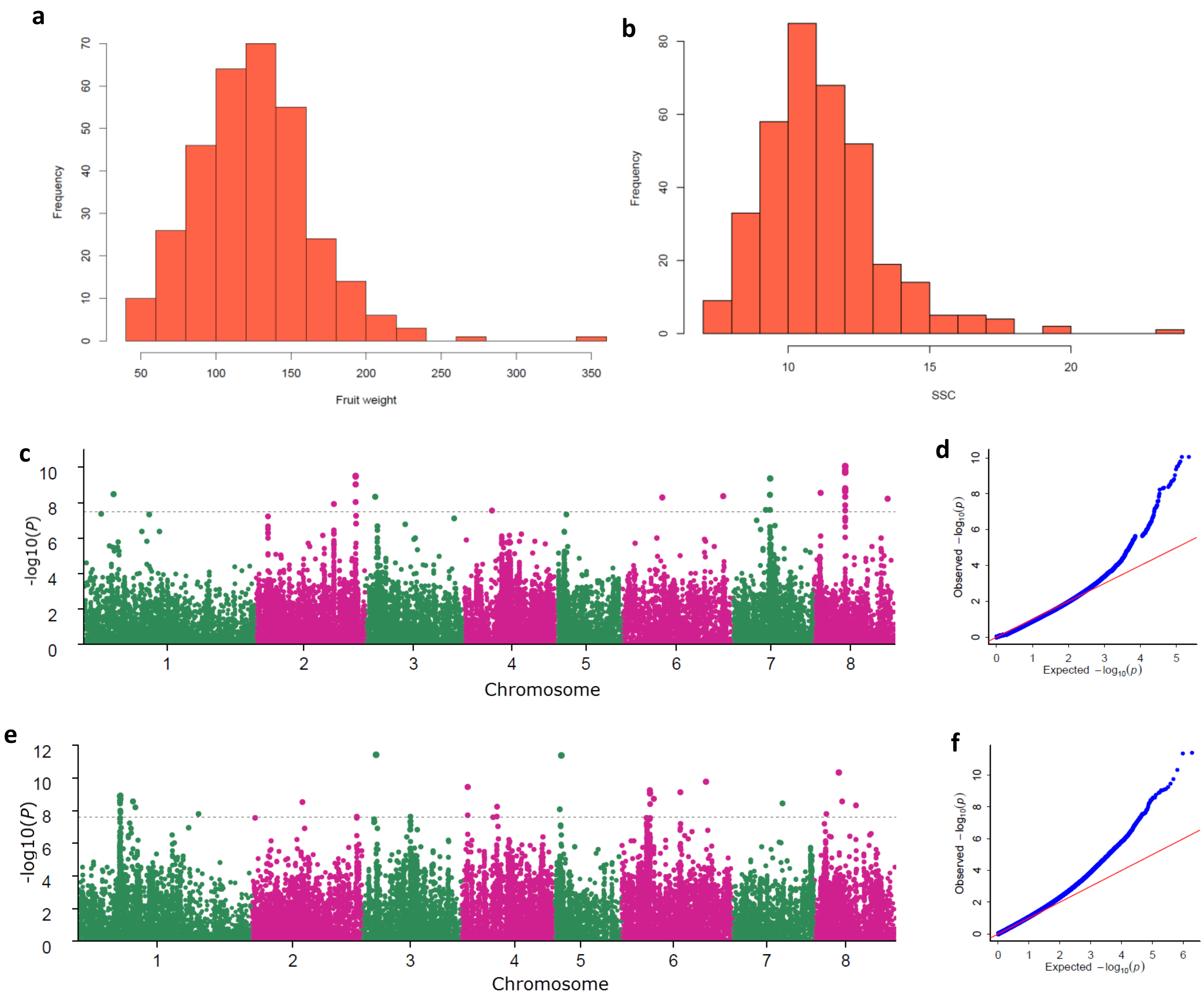

Figure S7 Genome-wide association studies (GWAS) of SSC and fruit weight. (a) Phenotype distribution of fruit weight. (**b**) Phenotype distribution of SSC. (**c**, **d**) Manhattan plot (**c**) and QQ plot (**d**) of GWAS on fruit weight. The horizontal dashed line in (**c**) indicates the Bonferroni-corrected significance threshold of GWAS ($P < 3.2 \times 10^{-8}$). (**e**, **f**) Manhattan plot (**e**) and QQ plot (**f**) of GWAS on SSC. The horizontal dashed line in (**e**) indicates the Bonferroni-corrected significance threshold of GWAS ($P < 2.6 \times 10^{-8}$).

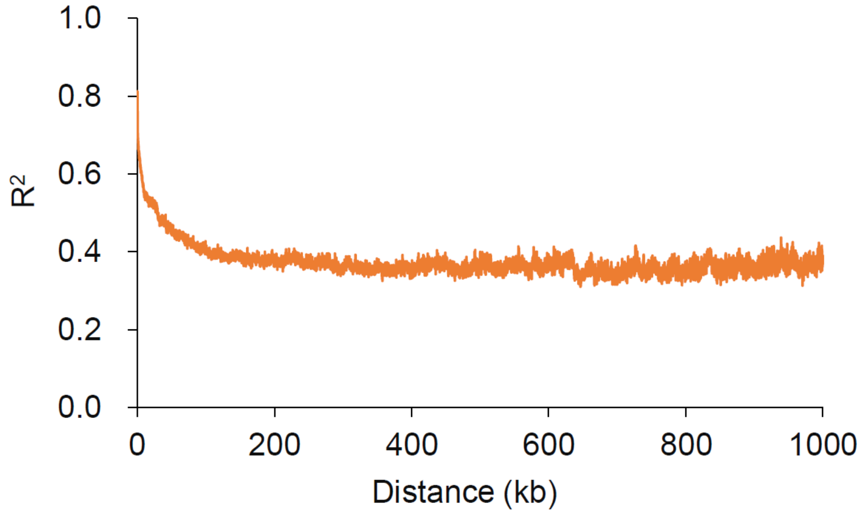

Figure S8 Regional LD decay measured by R^2 at the 20.0-30.0 Mb region of chromosome 2. The half LD decay distance was ~90 kb.

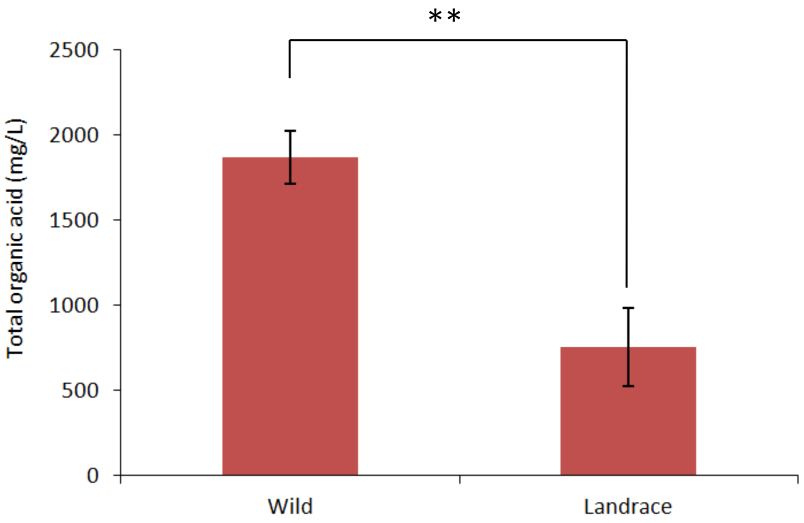

Figure S9 Total organic acids in domesticated peaches and wild relatives. ** indicates *P* < 0.01.

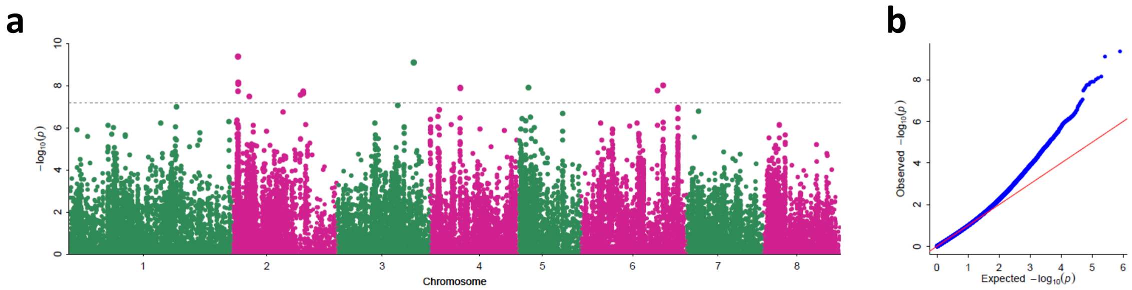

Figure S10 Genome-wide association study of total phenolic content for peach. (**a**) Manhattan plot. (**b**) QQ-plot. The horizontal dashed line in (**a**) indicates the Bonferroni-corrected significance threshold of GWAS ($P < 6.4 \times 10^{-8}$).

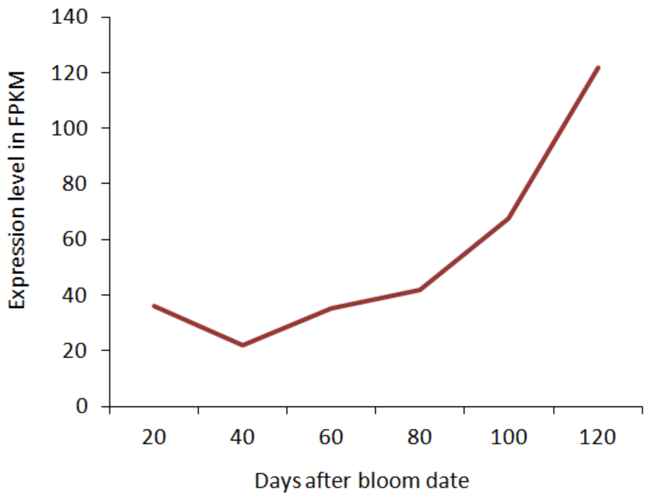

Figure S11 Expression profile of a sugar transport gene, *Prupe.4G037800*, during fruit development in peach.

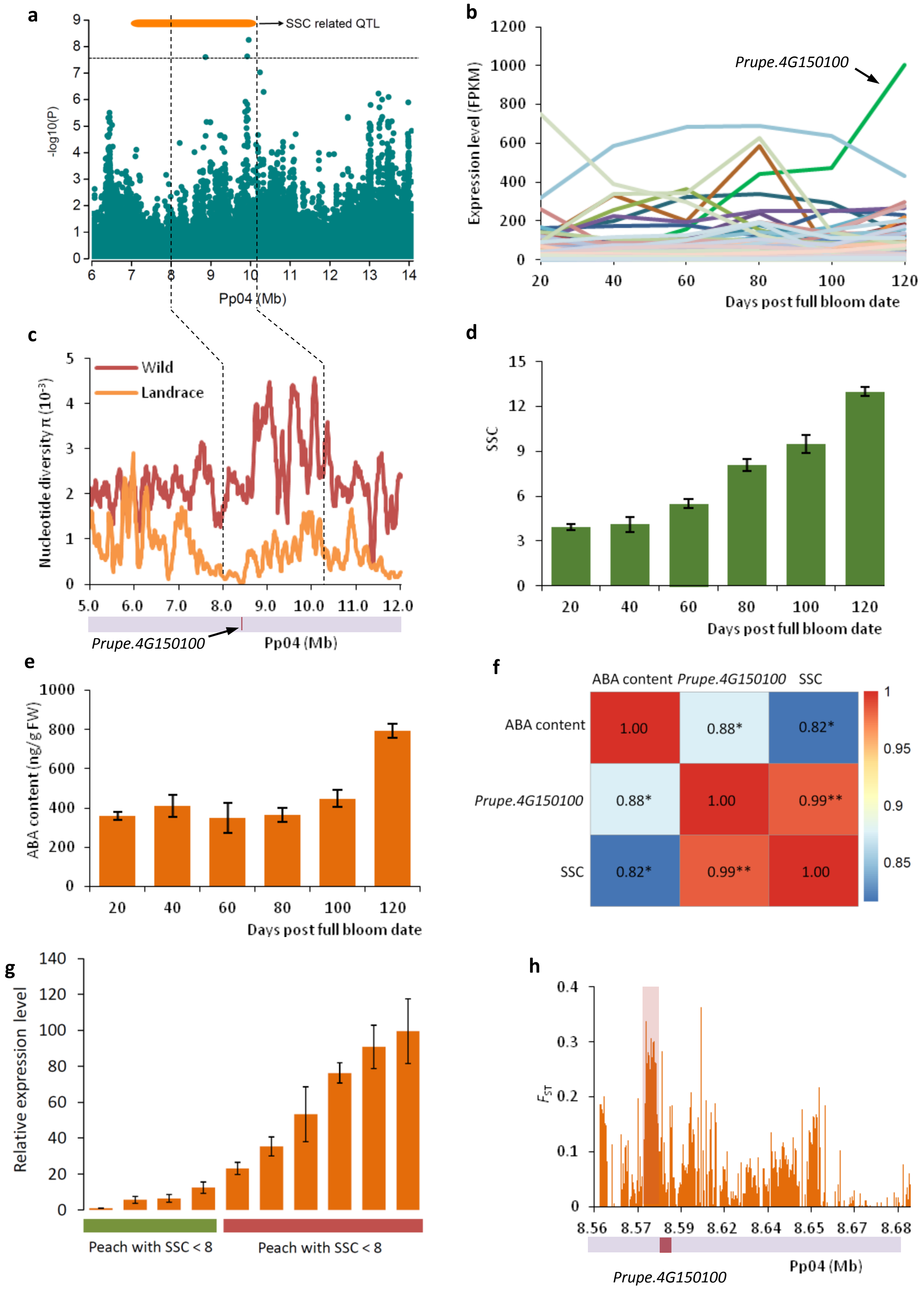
a
SSC related QTL
-log10(P)
Pp04 (Mb)
b
Prupe.4G150100
Expression level (FPKM)
Days post full bloom date
c
Wild
Landrace
Nucleotide diversity π (10⁻³)
Prupe.4G150100
Pp04 (Mb)
d
SSC
Days post full bloom date
e
ABA content (ng/g FW)
Days post full bloom date
f
ABA content
Prupe.4G150100
SSC
1.00
0.88*
0.82*
0.99**
g
Relative expression level
Peach with SSC < 8
Peach with SSC < 8
h
F_ST
Prupe.4G150100
Pp04 (Mb)

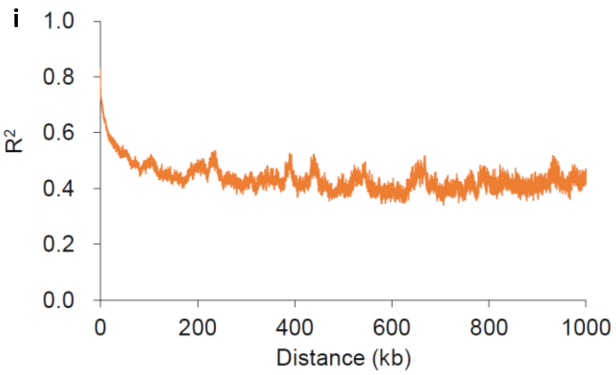

Figure S12 A candidate gene for SSC associated with increase of fruit taste during domestication. (**a**) Manhattan plot of an association peak for SSC on chromosome 4. Previously reported QTL related to SSC is shown as an orange bar at the top. The gray horizontal dashed line indicates the Bonferroni-correcred significance threshold of GWAS ($P < 2.6 \times 10^{-8}$). (**b**) Expression profiles of genes within the overlapping interval between GWAS associations and previously reported QTL. The candidate gene, *Prupe.4G150100*, is pointed by the arrow. (**c**) Nucleotide diversity of overlapping interval between GWAS associations and previously reported QTL. The position of the candidate gene is pointed by the arrow. (**d**) SSC at different stages of peach fruit development. Error bars represent standard deviation of five biological replicates. (**e**) ABA content of peach flesh at different stages of fruit development. Error bars represent standard deviation of three biological replicates. (**f**) Correlation matrix of ABA content, expression level of *Prupe4G150100*, and SSC. Pearson correlation coefficients are displayed. * denotes $P < 0.05$ and ** denotes $P < 0.01$. (**g**) Relative expression level of *Prupe.4G150100* in flesh of peach accessions with different SSC at maturity. (**h**) Genetic differentiation of genomic regions harbored *Prupe.4G150100*. The upstream region of *Prupe.4G150100* with high genetic differentiation is highlighted in shadow. (**i**) Regional LD decay of the ~5 Mb genomic interval around the *NCED3* locus on chromosome 4. Half of LD decay distance was ~150kb.

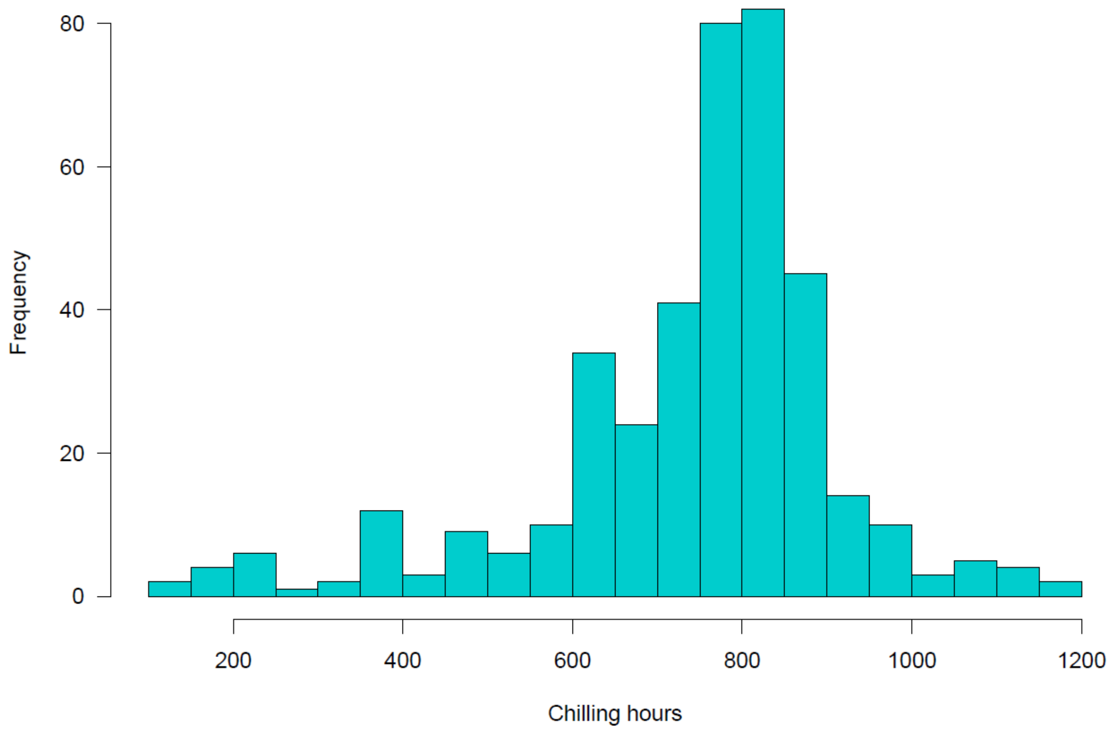

Figure S13 Distribution of chilling requirement in 371 peach accessions.

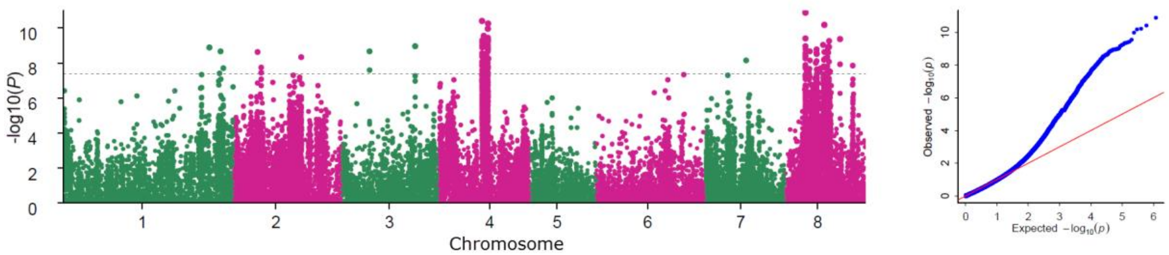

Figure S14 Manhattan plot (left) and QQ plot (right) of GWAS for chilling requirement (CR) by classifying accessions into low-CR and non-low-CR. The horizontal dashed line in the Manhattan plot indicates the Bonferroni-corrected significance threshold of GWAS ($P < 4.2 \times 10^{-8}$).

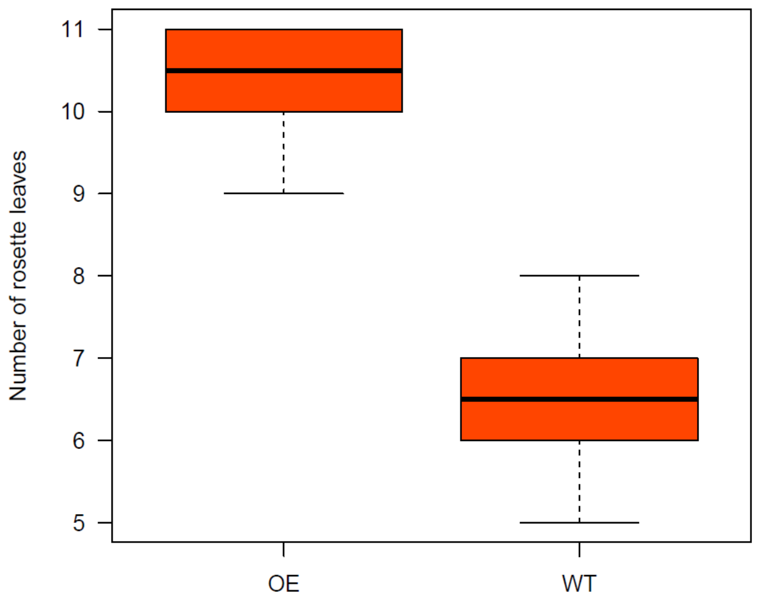

Figure S15 Comparison of flowering times between *Arabidopsis* over-expressing of peach *SVP* gene (OE) and the wild type (WT) *Arabidopsis*.

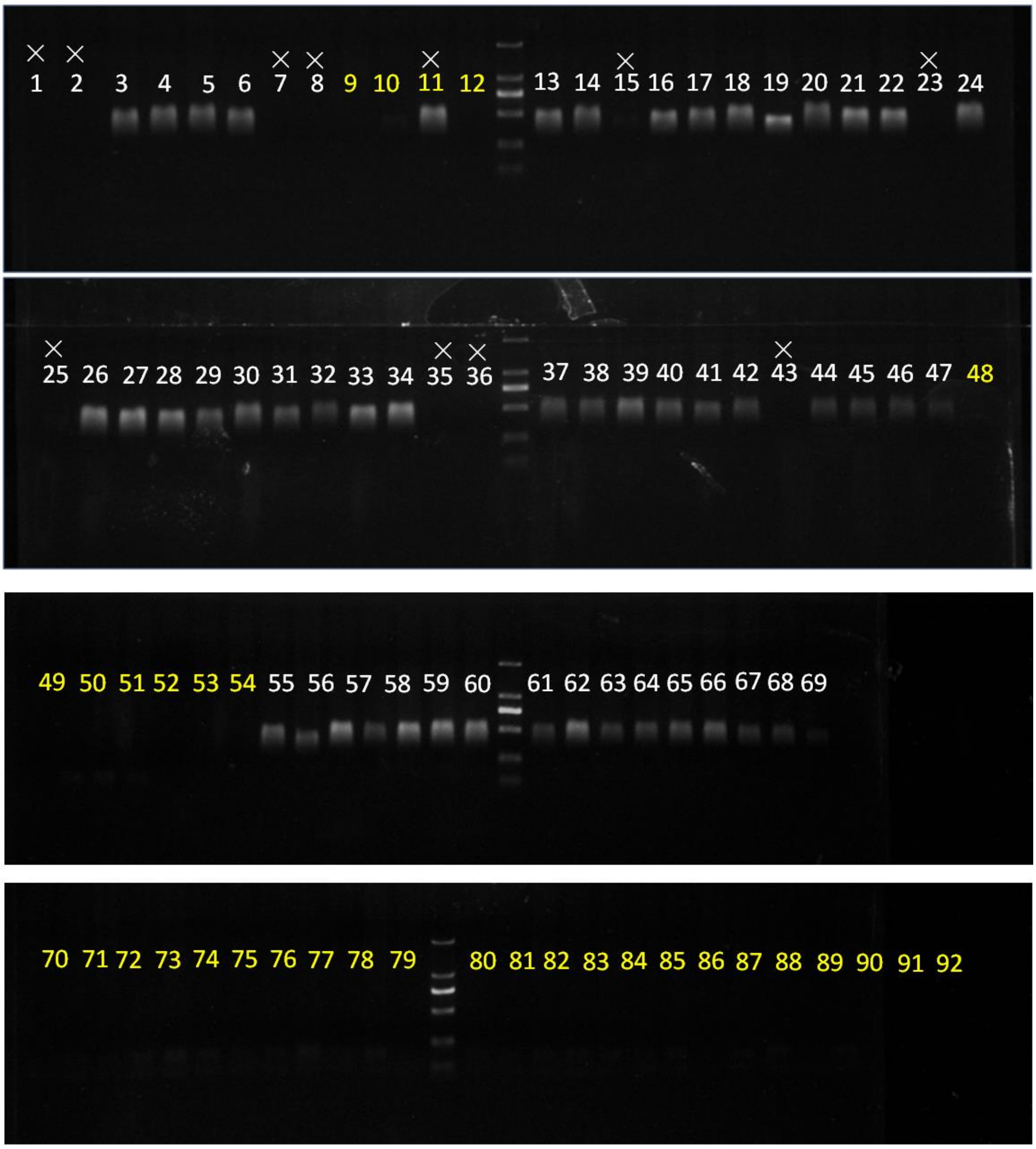

Figure S16 A PCR-based marker for low-CR peach identification based on the results of CR GWAS. F: AGTCAAATTTCACATTAAACACATT; R: CGGGGTTCAACATTAGGTGA. White numbers indicate the peach accessions with high CR (> 500 CHs). Yellow numbers indicate the peach accessions with low CR (< 500 CHs). White '×' indicate the inconsistent results.

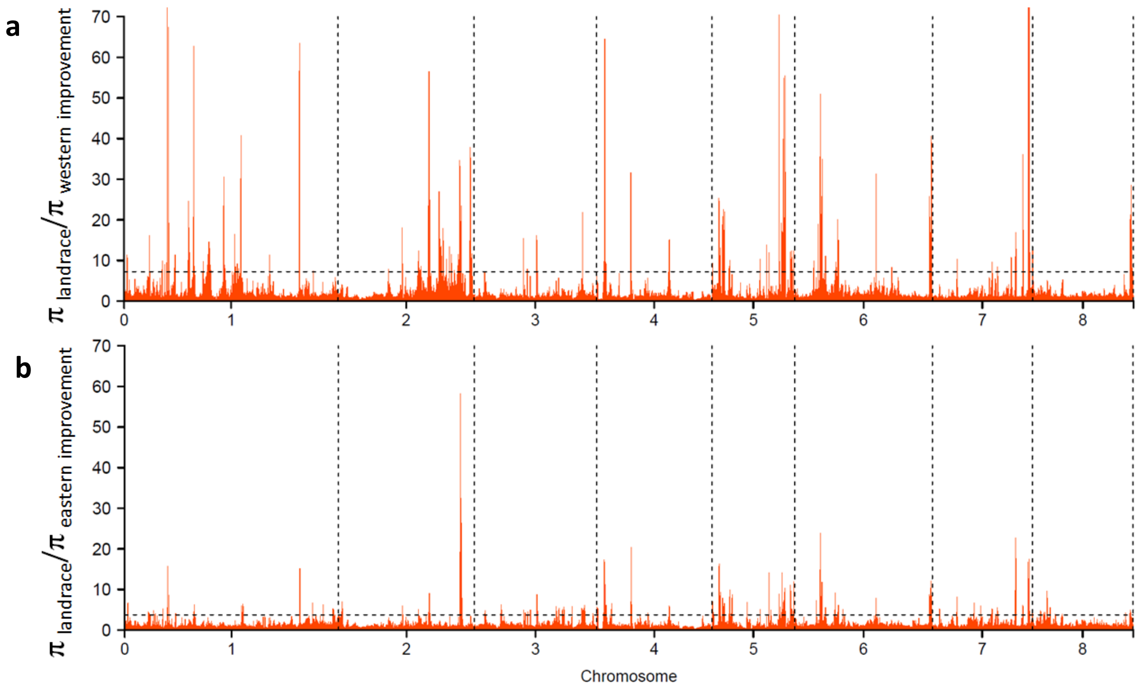

Figure S17 Comparison of improvement sweeps between eastern and western improved groups. (**a**) Improvement sweeps in western improved group. The horizontal dashed line indicates 5% cutoff ($\pi_{landrace}/\pi_{western\ improved} \geq 7.25$). (**b**) Improvement sweeps in eastern improved group. The horizontal dashed line indicates 5% cutoff ($\pi_{landrace}/\pi_{eastern\ improved} \geq 3.76$).

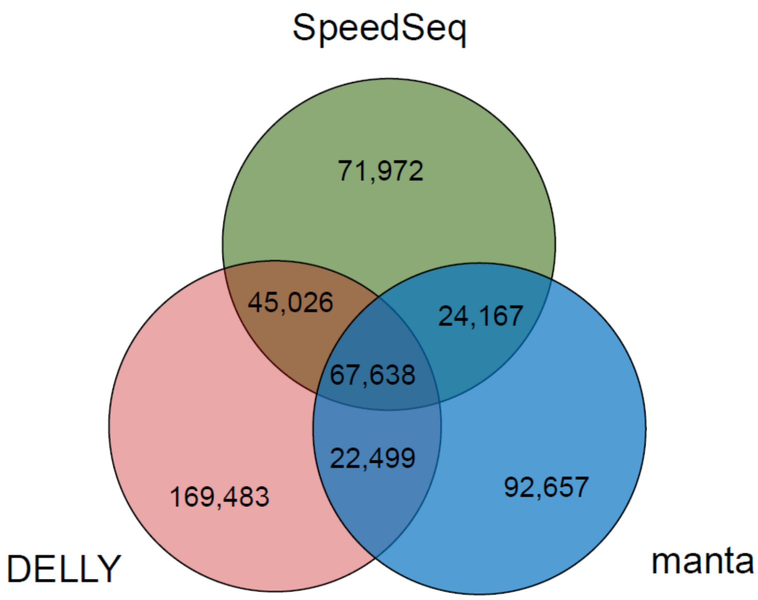

Figure S18 Shared and private SVs among three callers.

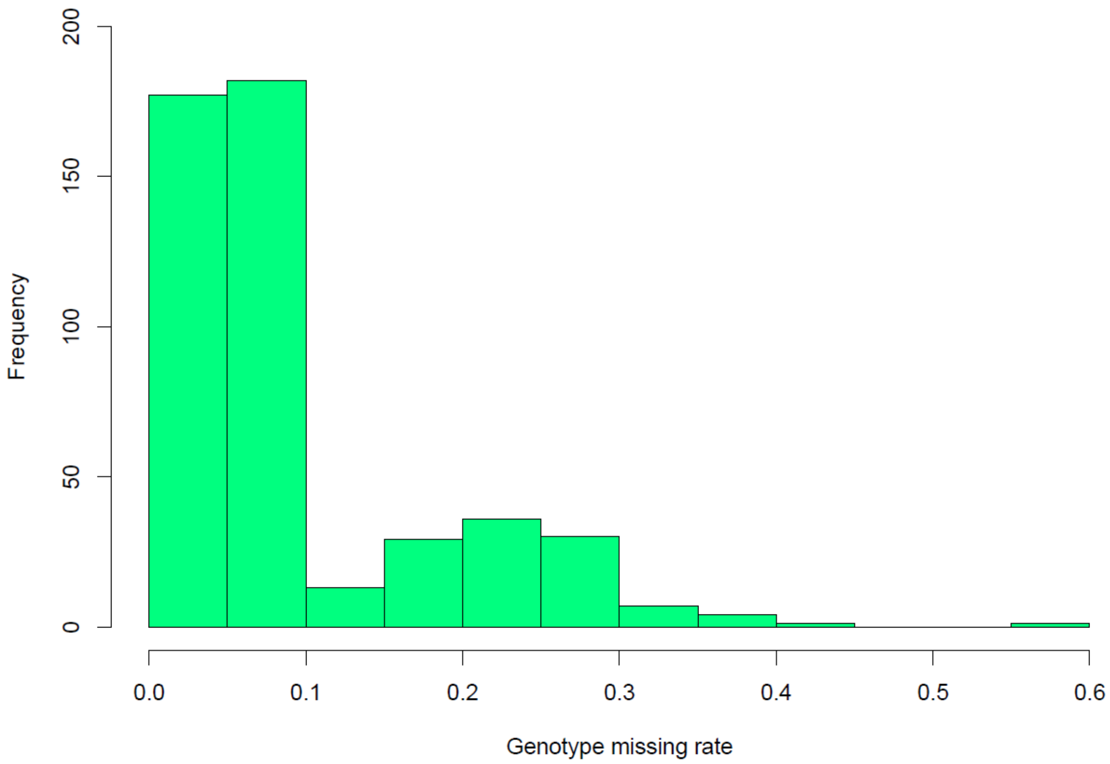

Figure S19 Distribution of genotype missing rate for 480 peach accessions.

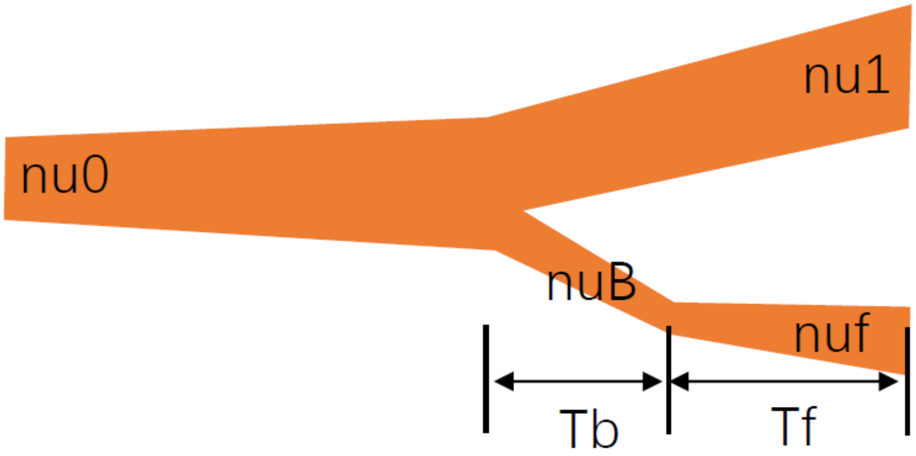

Figure S20 Demographic model for peach domestication and spread. nu0 indicates the effective population size of peach ancestors. A part of ancestor individuals was domesticated at 'Tb+Tf' years ago. nuB represents the effective population size of individuals at peach domestication. Tb indicates the duration times of bottleneck. Tf represents the times since domestication. nuf indicates the effective population size experiencing expansion since domestication. nu1 represent current population size of wild peach.

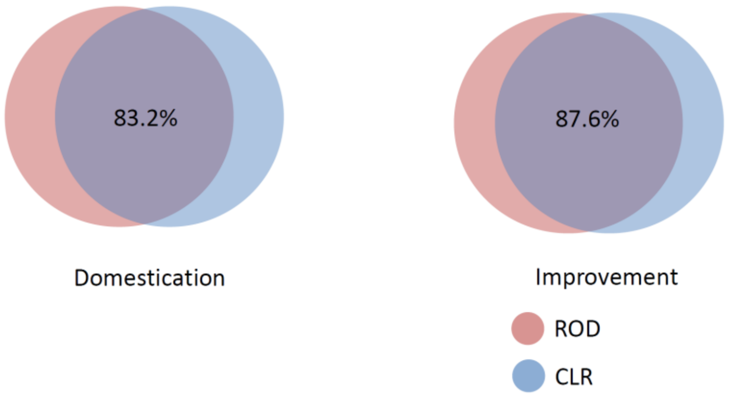

Figure S21 Shared domestication and improvement sweeps identified by ROD and XP-CLR methods.
