# Supplementary material for: Genomic analyses of an extensive collection of wild and cultivated accessions provide new insights into peach breeding history
Source: Genome Biol. 2019 Feb 21;20:36. doi: 10.1186/s13059-019-1648-9 (PMC6383288; doi:10.1186/s13059-019-1648-9)
Supplement: Supplementary file 4 — Review history. (DOCX 197 kb) [file 13059_2019_1648_MOESM4_ESM.docx]

**First round of review**

**Reviewer 1**

**Are the methods appropriate to the aims of the study, are they well described, and are necessary controls included?**
Yes the methods are appropriate.

**Are the conclusions adequately supported by the data shown?**

Yes

**Are sufficient details provided to allow replication and comparison with related analyses that may have been performed?**

Yes

**Does the work perform represent a significant advance over previously published studies? Please explain.**

This work is more like a complement of previously published work by the same team. However the present manuscript describes original data.

**Is the paper of broad interest to others in the field, or of outstanding interest to a broad audience of biologists?**

Yes: This work is a significant piece of work describing the use of large SNP dataset to unravel the recent history of a crop, especially the two phases of selection that are domestication and modern breeding.

**Comments to authors**

This manuscript describes an extensive study of the genomic variation of 480 wild and cultivated accessions of peach. The authors exploited this data to unravel the history of cultivated gene pool using genome scans of selection signatures. They unravelled the genomic regions that were under selection both during early domestication and subsequently during modern breeding phases. In addition, this work allowed ot identify some candidate genes (QTLs) involved in some important agronomic traits, such as fruit texture, organoleptic characteristics, fruit size, flseh color etc... The manuscript is well written and I have no major objection for its publication in GB. Even if the same authors have already published some GWAS results on peach, the present work includes some novel analyses on the history of peach domestication that deserve publication in a high profile journal. 
I have only very few comments listed below :
- In figure 1, the tree which his a phenetic tree (and not a phylogenetic tree) should be presented differently since one can hardly see its topology. Bootstrap values should also be included on the branches. This is necessary to conclude on the relationships between the groups that are discussed in the manuscript (even if the clustering looks quite clear).
- Maybe should the authors briefly comment on the fact that more QTLs are found in GWAS than in classical QTL mapping based on F2 (or BC) populations (eg p8 l54-57). This in fact illustrates one of the advantages of GWAS, as it allows to "grab" all the diversity of a pool of germplasm at once, as opposed of bi-parental populations where only differences between two genotypes can be unraveled. 
- Could the authors mention whether the SNPs associated with agronomic traits in modern varieties are new or whether they were already present in traditional germplasm ? This would provide some interesting discussions on whether favourable alleles can be found in traditional germplasm and subsequently selected for as opposed to being the result of recent mutations. 
- p11l20. the authors claimed that a region nof low diversity of 5 mb in the vicinity of the NCED3 locus could result from hitchiking. Hwever, never do they provide some information regarding the average LD size in the species. This may be useful to mention at this point of the manuscript.

**Reviewer 2**

**Are the methods appropriate to the aims of the study, are they well described, and are necessary controls included?**
The methods are appropriate to the aims of the study, and are well described.

**Are the conclusions adequately supported by the data shown?**

Mostly, except for the domestication bottleneck analysis.

**Are sufficient details provided to allow replication and comparison with related analyses that may have been performed?**

Not always. For example, no details are given for running the dadi software to infer population size history. The authors should provide a table listing model parameter values to show the strength, timing, and duration of the population bottleneck.

**Does the work perform represent a significant advance over previously published studies? Please explain.**

Compared to the last peach diversity and domestication study by the same authors (Cao et al, 2014, Genome Biol) that analysed 10 wild and 74 cultivated peach varieties, the current study represents a significant advance in two aspects. 1) a much larger collection (52 wild, 213 landrace, 215 improvement accessions, total 480) covering a world wide geographical area. 2) GWAS analysis of 11 agronomic traits.

**Is the paper of broad interest to others in the field, or of outstanding interest to a broad audience of biologists?**

Yes: The genomic analysis of peach evolution and breeding history is of broad interest. The accompanying sequence and variation data are valuable resources to botanists and geneticists alike.

**Comments to authors**

In the study "Genomic analyses of an extensive collection of wild and cultivated accessions provide new insights into peach breeding history" by Li et al, the authors performed a comprehensive analysis of peach evolution based on genome sequences of 480 wild and cultivated accessions and provided genetic evidence supporting distinct phases of domestication and improvement. The fruit size was found to be predominantly selected during domestication. The genome-wide association studies of 11 agronomic traits confirmed the results of previous studies and provided potential markers for molecular breeding. This work represents a significant advance over previous studies.

However, there are some aspects of the analysis that require major revisions to the manuscript before its acceptance for publication by Genome Biology. These are listed as follows:

Part 1: the analysis of domestication bottleneck

The authors found a strong domestication bottleneck unlike other fruit trees (apple, grape), and in disagreement with a previous study that the authors seem to be unaware of (see below). To rule out this strong bottleneck as an artifact of the analysis, the authors should address the following points:

1) Address a previous study showing "no evidence of a bottleneck during domestication" [Velasco et al 2016, G3, "Evolutionary genomics of peach and almond domestication"]

2) Page 18 lines 39-41, Page 6 lines 24-26 and Table S6. nucleotide diversity (pi) based estimate of bottleneck strength: 

There is a lack of test for sample duplicates and relatedness among the 480 accessions. As the nucleotide diversity will be underestimated due to potential sample relatedness, the authors should identify and exclude both duplicate and closely related samples (eg 1st degree relatives). The ratio of nucleotide diversities between wild and domesticate populations should be revised as a proper estimate of the bottleneck strength. 

Similarly, Fst estimate (Fig. 1f) should be revised as both nucleotide diversity and allele frequency are affected by the presence of duplicate and closely related samples.

Fig. 1f: the Fst between wild and "eastern" populations is missing

3) Page 18 lines 20-28: Dadi simulation: 
No sufficient details are given for the simulation to support the strong domestication bottleneck. The authors should provide the important details including:

a) specification and comparison of models, and the input data for the simulation
b) parameter estimates for the best model, inferred bottleneck strength, timing and duration
c) For readability, a figure to illustrate the model(s) and table to list the parameter values will be useful.

Page 6 line 56: are the estimated ~200 founders from dadi simulation?

4) Page 6 line 35 and table S7: BOTTLENECK simulation: abbreviations and result in table S7 should be explained. How strong is the bottleneck?

5) How different methods compare with each other?

Part 2: Structural variation (SV) analysis

It's not clear what the false positive rate for SV is. Since there is only one method used for calling SV, the authors should provide some validation as was done for SNPs in Table S4, or compare to another SV calling method to see how much the two methods overlap. Otherwise, the list of SVs will be of less value to other researches. 

Table S5: GWAS results. The SV type and size should also be provided.

The link to the SV vcf file is not working. Please make sure it is active.

**Response to reviewers**

**Reviewer #1:**

This manuscript describes an extensive study of the genomic variation of 480 wild and cultivated accessions of peach. The authors exploited this data to unravel the history of cultivated gene pool using genome scans of selection signatures. They unravelled the genomic regions that were under selection both during early domestication and subsequently during modern breeding phases. In addition, this work allowed to identify some candidate genes (QTLs) involved in some important agronomic traits, such as fruit texture, organoleptic characteristics, fruit size, flesh color etc... The manuscript is well written and I have no major objection for its publication in GB. Even if the same authors have already published some GWAS results on peach, the present work includes some novel analyses on the history of peach domestication that deserve publication in a high profile journal.

I have only very few comments listed below:

1. In figure 1, the tree which is a phenetic tree (and not a phylogenetic tree) should be presented differently since one can hardly see its topology. Bootstrap values should also be included on the branches. This is necessary to conclude on the relationships between the groups that are discussed in the manuscript (even if the clustering looks quite clear).

**Response**: We have added a phenetic tree with bootstrap values in the revised manuscript (Additional file 3: Figure S4). However, it is still hard to clearly show the bootstrap values for all branches; therefore, we have uploaded the phenetic tree in SVG format in the figshare database: https://figshare.com/articles/Phenetic_NJ-tree_of_480_peach_accessions/7241120

**2**. Maybe should the authors briefly comment on the fact that more QTLs are found in GWAS than in classical QTL mapping based on F2 (or BC) populations (eg p8 line54-57). This in fact illustrates one of the advantages of GWAS, as it allows to "grab" all the diversity of a pool of germplasm at once, as opposed of bi-parental populations where only differences between two genotypes can be unraveled.

**Response**: We thank the reviewer for the suggestion and have added a brief statement on this result in revised manuscript (Line 102-104 of Page 5).

**3.** Could the authors mention whether the SNPs associated with agronomic traits in modern varieties are new or whether they were already present in traditional germplasm? This would provide some interesting discussions on whether favourable alleles can be found in traditional germplasm and subsequently selected for as opposed to being the result of recent mutations.

**Response**: We extracted SNPs associated with agronomic traits from wild, landrace, and improved groups, and the allele frequencies in these groups were derived. As shown in the figure below, alleles of SNPs associated with the 11 agronomic traits in modern varieties are also present in landrace accessions, suggesting these SNPs are already present in landrace accessions, rather than from the recent mutations. Artificial selection during modern breeding (improvement) has changed the allele frequencies, leading to the increase of beneficial alleles. In addition, the favorable phenotypes in modern varieties can also be found in some landrace accessions, further supporting the genotypes for these favorable phenotypes are present in both modern varieties and landrace accessions.

We think this is not unexpected and does not provide much novel information so we did not include this result in the revised manuscript.

Allele frequency

|  | Wild allele 1 | Landrace allele 1 | Improved allele 1 | Wild allele 2 | Landrace allele 2 | Improved allele 2 |
| --- | --- | --- | --- | --- | --- | --- |
| Chilling requirement | 0.83 | 0.98 | 0.97 | 0.17 | 0.02 | 0.03 |
| Fruit weight | 0.192176 | 0.991385 | 0.990329 | 0.788593 | 0.00861507 | 0.00967096 |
| Soluble solid content | 0.83754 | 0.948958 | 0.991048 | 0.16246 | 0.0510421 | 0.00895166 |
| Total phenolics | 0.120093 | 0.933325 | 0.93655 | 0.879907 | 0.0666746 | 0.0634502 |
| Flesh adhesion | 1 | 0.970874 | 0.742424 | 0 | 0.0291262 | 0.257576 |
| Flesh color | 0.989362 | 0.731884 | 0.839572 | 0.0106383 | 0.268116 | 0.160428 |
| Fruit hairiness | 1 | 0.751724 | 0.657068 | 0 | 0.248276 | 0.342932 |
| Fruit shape | 0.0909091 | 0.933566 | 0.961735 | 0.909091 | 0.0664336 | 0.0382653 |
| Fruit skin color | 0.9375 | 0.891608 | 0.970745 | 0.0625 | 0.108392 | 0.0292553 |
| Fruit texture | 0.977778 | 0.748092 | 0.747475 | 0.0222222 | 0.251908 | 0.252525 |
| Pollen fertility | 1 | 0.996454 | 0.697861 | 0 | 0.0035461 | 0.302139 |

**Figure** Distributions of alleles frequency of associated SNPs in three peach groups.

**4**. p11l20. the authors claimed that a region of low diversity of 5 mb in the vicinity of the *NCED3* locus could result from hitchiking. However, never do they provide some information regarding the average LD size in the species. This may be useful to mention at this point of the manuscript.
**Response**: We thank the reviewer for pointing this out. We calculated the regional LD size of the 5-Mb genomic region around *NCED3* locus. The results showed that the regional LD decay of this 5-Mb region was 150kb (decaying to *R*^2^ of 0.41), which was significantly higher than the average of the genome wide level (~35kb). In addition, this locus is located at a strong domestication sweep supported by sharp ROD, high XP-CLR score, and high *F*_ST_ (Fig. 4f-h). Therefore, the selection on *NCED3* locus for high SSC is likely resulted from the hitchhiking of this 5-Mb genomic region. We have added the LD decay plot (Additional file 3: Figure S12i) and revised the text accordingly (Line 300-302 of Page 11).

**Reviewer #2:**
In the study "Genomic analyses of an extensive collection of wild and cultivated accessions provide new insights into peach breeding history" by Li et al, the authors performed a comprehensive analysis of peach evolution based on genome sequences of 480 wild and cultivated accessions and provided genetic evidence supporting distinct phases of domestication and improvement. The fruit size was found to be predominantly selected during domestication. The genome-wide association studies of 11 agronomic traits confirmed the results of previous studies and provided potential markers for molecular breeding. This work represents a significant advance over previous studies. However, there are some aspects of the analysis that require major revisions to the manuscript before its acceptance for publication by Genome Biology. These are listed as follows:

Part 1: the analysis of domestication bottleneck
The authors found a strong domestication bottleneck unlike other fruit trees (apple, grape), and in disagreement with a previous study that the authors seem to be unaware of (see below). To rule out this strong bottleneck as an artifact of the analysis, the authors should address the following points:
**1.** Address a previous study showing "no evidence of a bottleneck during domestication" [Velasco et al 2016, G3, "Evolutionary genomics of peach and almond domestication"]
**Response**: We thank the reviewer for bringing this up. In the paper of Velasco et al 2016, a total 14 cultivated peach and 14 almond accessions were sequenced. Through PSMC and MSMC analyses, the authors constructed the demographic history for peach, and found no bottleneck during its domestication. However, that study lacks the comparison of genetic diversity between cultivated and wild peach accessions as no wild peach accessions were included in that study. The different results between our study and the study by Velasco et al could be due to the different samples used, and we used a much larger population (480) of samples from different groups (wild, landrace and cultivated), while Velasco et al only used 14 cultivated peach accessions, in additional to 14 almond accessions. Nonetheless, we have provided multiple lines of evidence to support the strong domestication bottleneck (See below).

2. Page 18 lines 39-41, Page 6 lines 24-26 and Table S6. nucleotide diversity (pi) based estimate of bottleneck strength:
2.1 There is a lack of test for sample duplicates and relatedness among the 480 accessions. As the nucleotide diversity will be underestimated due to potential sample relatedness, the authors should identify and exclude both duplicate and closely related samples (eg 1st degree relatives). The ratio of nucleotide diversities between wild and domesticate populations should be revised as a proper estimate of the bottleneck strength.

**Response**: We thank the reviewer for pointing this out. To test the relationship among 480 accessions, we calculated the kinship matrix using TASSEL (version 5.0). We found more than 85.3% of the pairwise kinship values were close to or less than zero (please check figure below), indicating most peach accessions had no, or a weak relationship with other accessions in this panel. Although most of accessions had weak relationship, to estimate the nucleotide diversities and *F*_ST_ values more accurately, we removed six accessions with close pairwise relationships using the plink software (version 1.9). After removing these closely related samples, the nucleotide diversities and *F*_ST_ were re-calculated, which were largely identical to what we obtained before, and thus did not affect the related conclusions. We have updated the nucleotide diversities and *F*_ST_ values in the revised manuscript.

**Figure** Distribution of pairwise kinship among 480 peach accessions.

2.2 Similarly, F_ST_ estimate (Fig. 1f) should be revised as both nucleotide diversity and allele frequency are affected by the presence of duplicate and closely related samples.

**Response**: Please see our response above.

2.3 Fig. 1f: the Fst between wild and "eastern" populations is missing
**Response**: Thanks. The *F*_ST_ between wild and "eastern" populations has been added.

3. Page 18 lines 20-28: Dadi simulation:
No sufficient details are given for the simulation to support the strong domestication bottleneck. The authors should provide the important details including:
a) specification and comparison of models, and the input data for the simulation

**Response**: Thank you for pointing this out. We have added the details of the ∂a∂i analysis in the revised manuscript (Line 528-538 of Page 18-19).

b) parameter estimates for the best model, inferred bottleneck strength, timing and duration.
**Response**: The best fitting parameters has been listed in Additional file 2: Table S22.

c) For readability, a figure to illustrate the model(s) and table to list the parameter values will be useful.

**Response**: The figure (Additional file 3: Figure S20) and the table (Additional file 2: Table S22) for the model and best fitting parameters, respectively, have been added in the revised manuscript.

d) Page 6 line 56: are the estimated ~200 founders from dadi simulation?
**Response**: Yes, the number of founders was estimated from ∂a∂i simulation. We have made this clear in the revised manuscript (Line 163-164 of Page 6-7).

4. Page 6 line 35 and table S7: BOTTLENECK simulation: abbreviations and result in table S7 should be explained. How strong is the bottleneck?
**Response**: Thanks. Additional file 2: Table S7 have been updated. The BOTTLENECK program can only infer whether there exists a historical bottleneck or not, and can’t determine how strong the bottleneck is.

5. How different methods compare with each other?
**Response**: In this study, we used three different methods to detect the bottleneck during peach domestication. First, we compared nucleotide diversity between wild and landrace accessions, and the sharp reduction of nucleotide diversity (π_wild_/π_landrace_= 2.92) indicated a narrow bottleneck during domestication.

We then used the BOTTLENECK program (Version 1.2.02) to further verify the domestication bottleneck. The BOTTLENECK program infers historical bottleneck by comparing the expected heterozygosity estimated from allele frequencies with that estimated from the number of alleles and the sample size, which should be identical for a neutral locus in a population at mutation-drift equilibrium. The historical changes in population size are based on the principle that the expected heterozygosity estimated from allele frequencies decreases faster than that estimated under a given mutation model at mutation-drift equilibrium in populations that have experienced a recent reduction in size. Three models were included in the BOTTLENECK program, including Infinite Allele Model (IAM), Stepwise Mutation Model (SMM), two-phase model (TPM). To determine whether a population exhibits a significant number of loci with heterozygosity excess, three tests, namely a "sign test", a "standardized differences test", and a "Wilcoxon sign-rank test" were performed.

Finally, we used ∂a∂i (Diffusion Approximation for Demographic Inference) to infer the bottleneck. ∂a∂i infers population demographic history using diffusion approximation method based on multi-population allele frequency spectrum (AFS). The historical population size change, population splits and joins, and migration can be inferred using ∂a∂i. ∂a∂i approximates the joint multi-population AFS by numerical solution of a diffusion equation, and supports up to three simultaneous populations. The best fitting parameters for a specific demographic model were determined based on likelihoods through multiple round simulations and comparisons of the expected AFS using diffusion approach and observed AFS.

Although both BOTTLENECK and ∂a∂i are able to infer historical population size changes, the algorithms and strategies are different, BOTTLENECK is based on heterozygosity excess estimation, while the latter is diffusion approximation method.

Part 2: Structural variation (SV) analysis
**1**. It's not clear what the false positive rate for SV is. Since there is only one method used for calling SV, the authors should provide some validation as was done for SNPs in Table S4, or compare to another SV calling method to see how much the two methods overlap. Otherwise, the list of SVs will be of less value to other researches.
**Response**: We thank the reviewer for the valuable suggestion. Current SV calling programs tend to have a large number of false positives. To increase the robustness of our SV dataset, we used two additional programs, DELLY (version 0.7.8) and manta (version 1.4.0), to identify SVs. Both DELLY and manta discover SVs by integrating paired-end and split-read analyses. The SV calling described in our original manuscript was performed using SpeedSeq (version 0.1.2), whose core algorithm for SV calling is LUMPY that detect SVs by integrating read-pair, split-read and read-depth information. Only a total of 67,638 SVs were identified by all three programs and a total of 106,388 SVs were identified by at least two programs. Comparison of the results from the three SV calling programs is summarized in a Venn diagram (Additional file 3: Figure S18) in the revised manuscript. In the revised manuscript, we used the 159,330 SVs identified by at least two programs for downstream analyses.

**2**. Table S5: GWAS results. The SV type and size should also be provided.
**Response**: Table S5 has been updated accordingly.

**3**. The link to the SV vcf file is not working. Please make sure it is active.
**Response**: The link is active now. The compressed vcf file cannot be previewed, but can be downloaded through following link: https://ndownloader.figshare.com/files/13325288. In addition, we have also uploaded the vcf files of SVs called by manta and DELLY to the figshare: <https://figshare.com/articles/Peach_SVs/7238186>

**Second round of review**

**Reviewer 2**

The response and the revised manuscript looks good except for the estimation of sample relatedness, which the authors computed the kinship matrix (with negative values) which is not easy to interpret regarding the degree of relatedness. I suggest the authors use programs like vcftools —relatedness or KING et al, which explicitly estimates the coefficient of relatedness (thus the degree of relatedness) so related samples (incl. duplicates, 1st degree, maybe also 2nd degree) can be identified and excluded.

**Response**: Thanks for the suggestion. We inferred relationships among peach accessions using the latest version of the KING program (version 2.1.6). We did not identify any duplicated accessions or 1^st^-degree relatives in our peach collection, and only identified four 2^nd^-degree relative pairs involving nine samples (please check the figure below). We did recalculate nucleotide diversities and *F*_ST_ by excluding five samples from the four 2^nd^-degree relative pairs, and obtained nearly identical results as before. Therefore, we did not make any changes on our manuscript.
